# *Akkermansia muciniphila* alleviates antibiotic- and LPS-induced oxidative stress via the p38α MAPK–Nrf2 signaling axis

**DOI:** 10.3389/fmicb.2026.1753421

**Published:** 2026-02-10

**Authors:** Wen-Yu Ye, Cai-Xia Feng, Shi-Qi Su, Su-Mei Wei, Ying Mao, Zhen-Yu Chen, Ying Su, Qing-Wen Shan

**Affiliations:** 1 Department of Pediatrics, The First Affiliated Hospital of Guangxi Medical University, Nanning, China; 2 Difficult and Critical Illness Center, Pediatric Clinical Medical Research Center of Guangxi, Nanning, China

**Keywords:** *Akkermansia muciniphila*, antibiotic-associated enteropathy, intestinal barrier function, Nrf2 signaling, p38α MAPK

## Abstract

**Background:**

Antibiotic abuse and subsequent infection induce dysregulation of the intestinal epithelial kinome, characterized by p38α hyperphosphorylation (encoded by MAPK14), a common molecular trigger for barrier failure. Readily druggable nodes to repair this dysregulation remain elusive.

**Methods:**

Using an antibiotic-LPS co-exposure enteropathy model, we investigated whether *Akkermansia muciniphila* (AKK) exerts protective effects via modulation of specific host signaling pathways.

**Results:**

We found that AKK reactivates the “p38α MAPK–Nrf2” signaling pathway. Mechanistically, AKK specifically alleviates p38α subtype-mediated suppression of Nrf2, thereby synergistically enhancing the expression of antioxidant enzymes such as HO-1 and NQO1, reducing excessive reactive oxygen species (ROS) production, and restoring the integrity of epithelial tight junctions and mucus layers.

**Conclusion:**

Our work is the first to establish the “AKK–p38α MAPK–Nrf2” axis as a druggable kinase module for antibiotic-associated intestinal disease, providing an immediately translatable molecular foundation for developing oral, mechanism-defined, and precise microecological therapies

## 1 Introduction

According to the World Health Organization (WHO), 30–50% of all antibiotic prescriptions lack proper indication. Beyond fueling the pandemic of antimicrobial resistance (AMR), this misuse inflicts profound “collateral damage” by indiscriminately disrupting the gut microbiota, thereby initiating a detrimental cascade of intestinal mucosal oxidative stress (Reese et al., 2018). This oxidative cascade not only predisposes the host to secondary infections by opportunistic pathogens such as *Clostridioides difficile* and *Clostridium perfringens* but also, via the gut–liver and gut–brain axes, potentially drives the pathogenesis of systemic disorders including neurodegenerative diseases and metabolic syndrome (Sui et al., 2024; Morais et al., 2021). Consequently, elucidating the molecular mechanisms governing antibiotic-associated gut oxidative injury and developing effective countermeasures are of paramount importance for mitigating the adverse effects of antibiotics and improving patient outcomes.

Although different classes of antibiotics exhibit distinct antibacterial targets (e.g., bacterial cell wall, protein synthesis, or DNA replication), they commonly trigger dysregulation of the tricarboxylic acid (TCA) cycle and hyperactivation of the electron transport chain in bacterial cells. This metabolic disruption promotes the disintegration of iron–sulfur clusters and the release of free Fe^2+^, which subsequently drives a surge in intracellular reactive oxygen species (ROS) via the Fenton reaction, ultimately contributing to bacterial killing (Van Acker and Coenye, 2017). However, this ROS-dependent bactericidal mechanism concurrently inflicts substantial impact on the host intestinal microecology (Wang et al., 2024; Palleja et al., 2018), constituting a “second hit” to the enterocyte kinome. Antibiotic-driven microbiota disruption markedly reduces the production of short-chain fatty acids (SCFAs), forcing intestinal epithelial cells to shift toward aerobic glycolysis. This metabolic reprogramming elevates luminal oxygen pressure, compromises the physiological anaerobic environment, and ultimately leads to excessive ROS accumulation in mucosal tissue, thereby disrupting the intestinal barrier (Zhang et al., 2022).

Notably, antibiotic-induced gut dysbiosis promotes the production of substantial amounts of lipopolysaccharide (LPS) by Gram-negative bacteria. Upon breaching the intestinal barrier, LPS binds to Toll-like receptor 4 (TLR4), which not only activates the NF-κB pathway to drive the release of pro-inflammatory cytokines but also directly suppresses the transcription factor Nrf2-mediated antioxidant pathway, thereby exacerbating both inflammation and ROS accumulation (Zhang et al., 2022; Tang et al., 2021; Huang et al., 2024). More critically, excessive ROS buildup leads to redox-mediated modification of key cysteine residues in p38α MAPK, locking it into a hyperphosphorylated state. This sustained p38α MAPK activation consequently impairs the Nrf2-driven antioxidant program, creating a self-reinforcing cycle that perpetuates both inflammatory and oxidative stress responses (Mao et al., 2024). The uncontrolled generation of ROS non-selectively attacks vulnerable phospholipids in the membranes of intestinal epithelial cells—affecting both bacterial and host cells—resulting in epithelial injury, increased mucosal permeability, and ultimately barrier dysfunction (Laforgia et al., 2018). Furthermore, endogenously sustained oxidative stress serves as a key driver for the development of antibiotic resistance (Carvajal-Garcia et al., 2023). Based on this mechanistic cascade, we propose that resetting the p38α MAPK–Nrf2 kinase module may represent a promising strategy to disrupt this vicious cycle and alleviate intestinal oxidative stress. However, to date, no orally available, mechanism-defined modulators of the p38α MAPK–Nrf2 module have been developed for clinical use.

In recent years, *Akkermansia muciniphila* (AKK) has emerged as a key target in the development of next-generation probiotics due to its distinctive antioxidant properties as a gut commensal bacterium, including high oxygen reductase activity (2.26 ± 0.99 mU/mg) and broad-spectrum tolerance to antibiotics (Luo et al., 2022; Ouwerkerk et al., 2016). However, although its clinical potential is emerging, the precise molecular mechanism through which AKK antagonizes intestinal oxidative stress in antibiotic-associated infection models remains elusive, significantly impeding its translation into a mechanism-targeted therapeutic agent. This study aims to transcend the conventional “microbiota replacement” paradigm by addressing a more fundamental question: Can AKK mitigate intestinal oxidative stress induced by the synergistic effects of antibiotics and LPS through precise regulation of the host cellular p38α MAPK-Nrf2 kinase module? We will systematically evaluate the integral efficacy of AKK in restoring intestinal barrier function, mitigating oxidative damage, and reshaping microbial architecture. Our findings are expected to provide a solid theoretical foundation for establishing AKK as a next-generation microecological therapeutic with a well-defined mechanism of action.

## 2 Materials and methods

### 2.1 Main reagents and drugs

LPS (L2880, *Escherichia coli* O55:B5) was purchased from Sigma (St. Louis, MO, USA). Ampicillin (A9518-25G-9), Metronidazole (M1547-25G), Neomycin sulfate (N6386-5G), Gentamicin sulfate (E003632-1 g) and Vancomycin (1404-93-9) were all purchased from Sigma-Aldrich (United States), with all compounds having a purity of >95%. p38α MAPK inhibitor (SB203580) was purchased from MCE (Shanghai, China). Antibodies against p-p38α MAPK (#4511), HO-1(#43966), NQO-1(#3187) were purchased from Cell Signaling Technology (Danvers, MA, United States). Antibodies against p38α MAPK (#R25239), Occludin(#502601), was purchased from zenbio (Chengdu, China). Antibodies against Claudin 1(#AF0127) was purchased from Affinity Biosciences (Liyang, China). Antibodies against ZO-1(#21773-1-AP), Nrf2(#16396-1-AP), *β*-actin(#20536-1-AP) was purchased from Proteintech (Rosemont, IL, United States).

### 2.2 Preparation and activity characterization of AKK

*Akkermansia muciniphila* strain (ATCC BAA-835) was obtained from the Guangdong Microbial Culture Center (GDMCC, Guangdong, China) and cultured following the GDMCC’s standard cultivation protocols. AKK was cultured in Brain Heart Infusion (BHI, Solarbio, China) liquid medium, supplemented with 0.5% (wt/vol) mucin at 37 °C under anaerobic conditions. Following incubation under anaerobic conditions at 37 °C, the bacterial culture was centrifuged at 4 °C for 15 min at 7,000 g and washed twice with sterile phosphate-buffered saline (PBS) (Xia et al., 2022). The resulting bacterial pellet was resuspended in PBS to prepare bacterial suspensions at concentrations of 10^8^ CFU/mL for subsequent experimental use.

### 2.3 *In vitro* cell culture methods

#### 2.3.1 Cell culture and treatment

Ncm460 (Baidi Biotech Ltd., Zhejiang, China) were cultured in RPMI 1640 containing 1% penicillin–streptomycin solution (Solarbio, Beijing, China) and 10% fetal bovine serum (Sigma, St. Louis, MO, USA). The cells were cultured in a humidified incubator with a constant temperature (Heal Force, Shanghai, China) at 37 °C under an atmosphere of 5% CO_2_. When cell confluence reached 80–90% (after approximately 2–3 days), subculturing was performed. Cells were treated with LPS (10 μg/mL) for 24 h in culture medium. Following LPS stimulation, the cells were co-cultured with AKK for 12 h.

To elucidate the role of p38α MAPK, we utilized a siRNA-mediated knockdown strategy in Ncm460 cells. After 48 h of transfection and validation of p38α MAPK downregulation by western blot analysis, the cells were exposed to LPS challenge and co-cultured with *A. muciniphila* for 12 h prior to assessment.

#### 2.3.2 Cell viability assay

We measured cell viability with a Cell Counting Kit-8 (CCK-8, Beyotime). Ncm460 cells (5,000 cells/well in 96-well plates) underwent various treatments, were incubated with CCK-8 reagent for 2 h, and then analyzed at 450 nm.

#### 2.3.3 Flow cytometry analysis

Ncm460 cells were subjected to flow cytometric analysis for ROS following experimental treatments. For ROS detection, cells were incubated with 10 μM 2′,7′-dichlorodihydrofluorescein diacetate (DCFH-DA, Sigma-Aldrich) at 37 °C for 20 min in the dark, then washed twice with PBS before immediate analysis using the FITC channel (Ex/Em = 488/525 nm). All samples were analyzed on a BD FACS Canto II flow cytometer (BD Biosciences) with ≥10,000 events recorded per condition. Data processing was performed using FlowJo v10.8 software (Tree Star), with gating strategies established using untreated controls and fluorescence-minus-one (FMO) controls for compensation.

#### 2.3.4 Cell scratch wound healing assay

To evaluate cellular migratory capacity, a cell scratch wound healing assay was performed. Briefly, Ncm460 cells were seeded into 6-well plates and cultured in RPMI 1640 medium supplemented with 10% fetal bovine serum (FBS) under standard conditions (37 °C, 5% CO_2_) until reaching 100% confluence (approximately 24–48 h). A straight wound was created in the confluent monolayer using a sterile 200 μL pipette tip. The cells were then gently washed twice with pre-warmed PBS to remove detached cells. Subsequently, the medium was replaced with serum-free RPMI 1640 to eliminate the influence of serum on cell migration. Cells were further incubated for 24 h under the same culture conditions. Images of the wound area were captured at the same location immediately after scratching (0 h) and at 24 h post-scratch using an inverted microscope. The wound area was measured using ImageJ software (National Institutes of Health, USA), and the cell migration rate was calculated according to the following formula:


$$\text{Migration rate}\;(\%)=[(A_{0}–A_{24})/A_{0}]\times 100\%$$

Where A_0_ and A_24_ represent the wound area at 0 h and 24 h, respectively.

#### 2.3.5 Evaluation of cellular oxidative stress markers

Commercial assay kits (Jiancheng Bioengineering Institute, Nanjing, China) were used to determine the levels of reduced glutathione (GSH) and malondialdehyde (MDA), as well as the activity of superoxide dismutase (SOD), in colonic tissue homogenate supernatants and Ncm460 cell lysates, following the manufacturer’s instructions. Briefly, collected cells or homogenized colon tissues were centrifuged, and the resulting supernatants were used for analysis. For GSH assay, the supernatant was reacted with the assay reagents at room temperature for 5 min, and absorbance was measured at 405 nm. For MDA determination, the supernatant was mixed with reagents and incubated at 95 °C for 40 min. After cooling, absorbance was measured at 530 nm. For SOD activity, the supernatant was incubated with working solution at 37 °C for 20 min, and absorbance was measured at 450 nm. The protein concentration of each supernatant was quantified using a BCA protein assay kit for normalization of enzymatic activities.

#### 2.3.6 RNA extraction and quantitative real-time PCR (Cell-based)

Total RNA was extracted from Ncm460 cells using NucleoZol reagent (740404.200, Gene Co. Ltd), with RNA concentration and purity determined by UV–Vis Spectrophotometer (NanoDrop) at 260 nm. Reverse transcription was performed using HiScript III RT SuperMix for qPCR (+gDNA wiper) (R323-01, Vazyme, Nanjing, China) to obtain complementary DNA (cDNA). Quantitative PCR was subsequently carried out on a QuantStudio™ 5 system (Applied Biosystems, CA, USA) using ChamQ Universal SYBR qPCR Master Mix (Q711-03, Vazyme, Nanjing, China), with all primers synthesized by Genesys Biotech Co. Ltd. (Beijing, China) (Supplementary Table S1). Relative gene expression levels were calculated using the 2^−ΔΔCT^ method with GAPDH serving as the endogenous control.

#### 2.3.7 Western blotting (cell-based)

Western blotting was performed using both Ncm460 cells at logarithmic growth phase samples. The samples were lysed in RIPA buffer containing 1% PMSF protease inhibitor (with additional 1% phosphatase inhibitor for phosphoprotein extraction) on ice for 30 min, followed by centrifugation at 12,000 ×*g* for 15 min at 4 °C to collect supernatants. Protein concentrations were determined by BCA assay (Beyotime, Beijing, China), and samples were diluted with 5 × loading buffer followed by denaturation at 100 °C for 5–10 min. Equal amounts of protein (20 μg per sample) were separated by 8–12% SDS-PAGE and subsequently transferred to PVDF membranes. After blocking with 5% skim milk or 5% Bull Serum Albumin (BSA) for 1.5 h at room temperature, the membranes were incubated overnight at 4 °C with primary antibodies, washed three times with TBST, and then incubated with corresponding HRP-conjugated secondary antibodies (1:20,000 dilution) for 1 h at room temperature. Protein bands were visualized using ECL chemiluminescent substrate (Biosharp, Hefei, China). For sequential detection of multiple targets from the same membrane, bound antibodies were stripped using a mild stripping buffer before reprobing. Band intensities were quantified by densitometric analysis using ImageJ software, with *β*-actin serving as the loading control for normalization.

In all *in vitro* cell experiments, Western blot and quantitative PCR analyses were carried out with a minimum of 6 independent biological replicates (*n* ≥ 6). Each replicate utilized cells from different passages and was conducted independently on different dates. Cell viability assessment and ROS flow cytometry analysis were performed with a minimum of 3 independent biological replicates (*n* ≥ 3).

### 2.4 *In vivo* animal experiment methods

#### 2.4.1 Experimental animals and grouping

Healthy male C57BL/6 J mice (Specific Pathogen-Free, SPF grade, aged 5–6 weeks; average body weight 20–25 g) were obtained from the Experimental Animal Center of Guangxi Medical University (License No. SCXK Gui 2020-0003). All animals experimental procedures were conducted in accordance with the guidelines approved by the Ethics Committee of the First Affiliated Hospital of Guangxi Medical University. The animals were acclimatized for 1 week under standardized SPF conditions in ventilated cages within a controlled environment (temperature: 22–25 °C; humidity: 50–70%; 12:12 h light–dark cycle). Throughout the study, animals had ad libitum access to autoclaved water and a standard SPF-compliant rodent diet.

After a one-week acclimatization period, the animals were randomly allocated into 6 groups using a random number table method (*N* = 6): Normal Control (NC), model control (NS + ABX/LPS), low-dose AKK group (AKK(L) + ABX/LPS), medium-dose AKK group (AKK(M) + ABX/LPS), high-dose AKK group (AKK(H) + ABX/LPS), and p38α MAPK inhibitor group (SB203580 + ABX/LPS). The NC group remained untreated throughout the experiment and was maintained on a standard diet with ad libitum access to water. The remaining 5 groups were first administered a continuous antibiotic cocktail (ABX, The total volume is 200 μL per animal per time) of ampicillin (1 mg/mL), metronidazole (1 mg/mL), gentamicin (1 mg/mL), neomycin sulfate (1 mg/mL), and vancomycin hydrochloride (0.5 mg/mL) via gavage twice daily (at approximately 12-h intervals) for 7 days to induce intestinal oxidative stress. On day 8, LPS (5 mg/kg) was intraperitoneally injected to exacerbate intestinal barrier damage. Subsequently, the NS + ABX/LPS group received 0.9% Normal Saline (NS), while the AKK(L) + ABX/LPS, AKK(M) + ABX/LPS, and AKK(H) + ABX/LPS groups were treated with low (10^6^ CFU/mouse, via 200 μL of a 5 × 10^5^ CFU/mL suspension, twice daily), medium (10^7^ CFU/mouse, via 200 μL of a 5 × 10^6^ CFU/mL suspension, twice daily), and high (10^8^ CFU/mouse, via 200 μL of a 5 × 10^7^ CFU/mL suspension, twice daily) doses of AKK, respectively. The SB203580 + ABX/LPS group received the p38α MAPK inhibitor. The total experimental duration was 15 days, comprising 7 days of antibiotic cocktail administration, a single LPS challenge on day 8, and subsequent 7-day intervention with AKK or p38α MAPK inhibitor.

**Figure 1:**
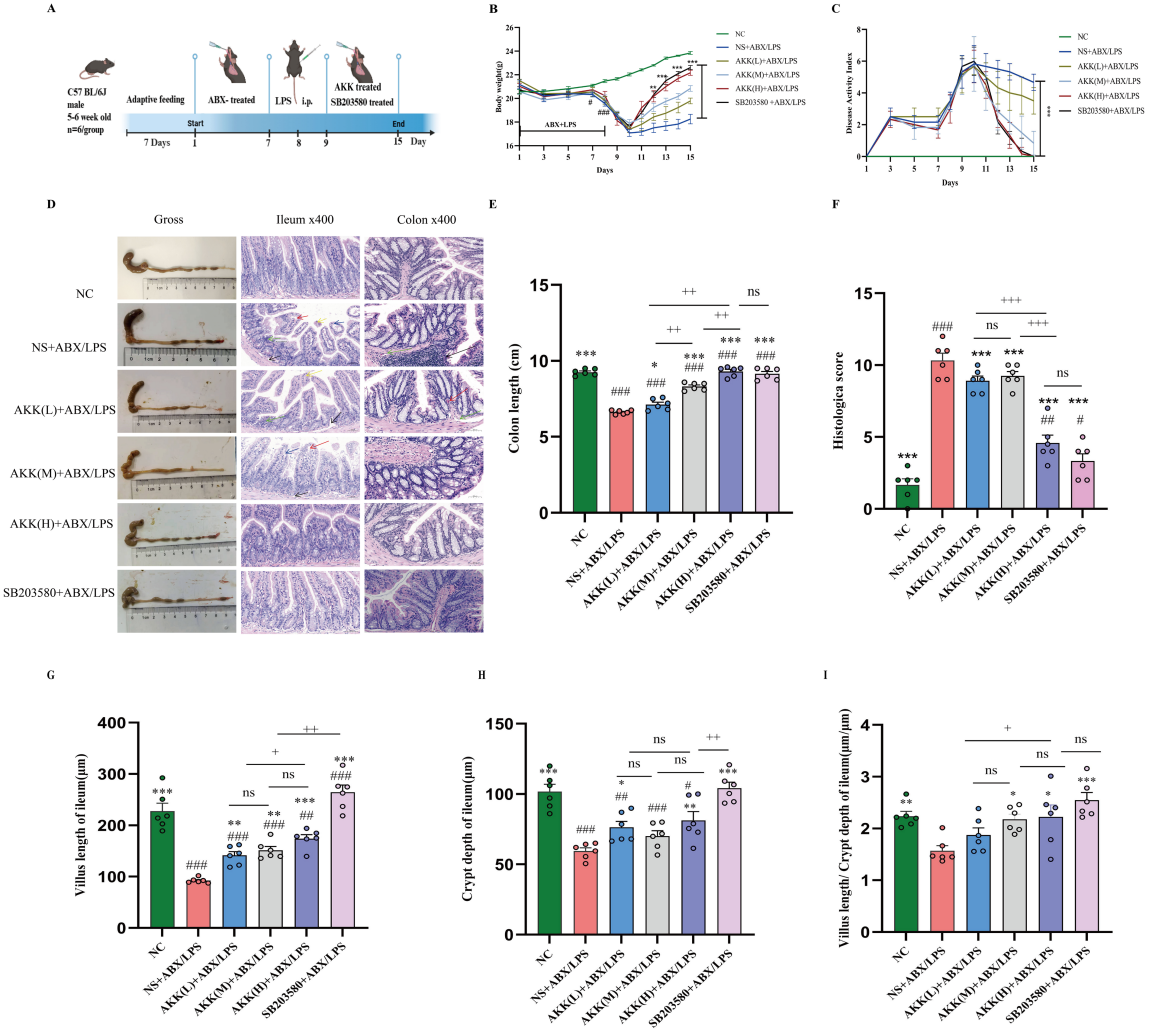
AKK alleviates ABX/LPS-induced pathological remodeling and inflammation in murine intestine via inhibition of the p38α MAPK pathway. **(A)** Schematic of the experimental design, illustrating the timeline of ABX/LPS challenge and the interventions with AKK or the p38α MAPK inhibitor (SB203580). **(B)** Body weight changes of mice in different treatment groups. **(C)** Disease activity index. The data points represent the daily measurements. To make the graph clear, the data at key time points are shown in the figure. **(D)** Representative images showing: (i) The gross appearance of the colon and cecum; (ii) H&E-stained sections of the ileum; and (iii) H&E-stained sections of the colon. **(E)** Quantitative analysis of colon length. **(F)** Histopathological colitis scores. **(G–I)** Morphometric analysis of the ileum, including villus height, crypt depth, and the villus height-to-crypt depth ratio (VCR). *n* = 6 independent biological replicates. All data are presented as mean ± SEM. ^#^*p* < 0.05, ###*p* < 0.001, ####*p* < 0.0001 versus NC group; *p < 0.05, **p > *p* < 0.01, ***p < 0.001, ****p < 0.0001 versus NS + ABX/LPS group, +*p* < 0.05, ++*p* < 0.01, +++*p* < 0.01, +++*p* < 0.001, *+++p* < 0.0001 versus the AKK-treated group.

Throughout the study, body weights were measured and recorded daily. Mice were monitored daily for general health status, including mental state, food and water intake, physical activity, and the presence of diarrhea. Figure 1A shows the study program and design. To eliminate assessment bias, all outcome evaluations (including disease activity index scores and histopathological scores) were conducted under conditions where the researchers were unaware of the grouping information.

#### 2.4.2 DAI score

The disease activity index (DAI) was calculated by integrating scores for weight loss, stool consistency, and fecal bleeding, as previously described (Table 1; Yu et al., 2022).

**Table 1:** Disease activity index.

Score	Weight loss (%)	Stool consistency	Bleeding
0	0	Normal	Negative
1	1–5%	Loose but formed	Weakly positive
2	5–10%	Loose unformed	Positive
3	10–20%	Very loose	Markedly positive
4	20%	Severe diarrhea	Rectal bleeding

#### 2.4.3 Sample collection

On day 14, the animals were anesthetized using 1.5% isoflurane and subsequently euthanized via cervical dislocation. The abdominal cavity was opened via midline incision, and fecal samples (10 g) were collected from the ileocecal region and stored at −20 °C for 16S rRNA sequencing. The entire colon was dissected from the ileocecal junction to the rectum, and its length was measured. A 1 cm segment of distal colon was excised, rinsed with ice-cold PBS to remove luminal contents, and divided for fixation in 4% paraformaldehyde (for H&E staining and IHC) or snap-frozen in liquid nitrogen for storage at −80 °C (for WB and RT-PCR et al.). For *in vitro* experiments, Ncm460 in the logarithmic growth phase were treated with LPS, followed by incubation with AKK or p38α MAPK inhibitor. Cells were then harvested for RNA/protein extraction and subsequent analysis by RT-PCR, WB, and flow cytometry.

#### 2.4.4 Histology and immunohistochemistry

Colon specimens were processed through standard dehydration and paraffin embedding, then sectioned at 5 μm thickness. Following xylene deparaffinization and gradient alcohol rehydration, tissue sections were stained with hematoxylin and eosin (H&E) for histological examination. Morphological evaluation was performed under light microscopy with systematic scoring of: (1) mucosal damage (grade 0–3), (2) inflammatory cell infiltration (grade 0–3), (3) crypt destruction (grade 0–3), (4) mucosal hemorrhage (grade 0–3), and (5) interstitial edema (grade 0–3), with six randomly selected fields analyzed per section. For immunohistochemical analysis, colon sections were incubated with anti-MUC2 antibody (Abcam) followed by appropriate secondary antibodies. Six independent fields per specimen were examined at 40 × magnification, and MUC2 expression was quantified using ImageJ software by calculating the percentage of positively stained area (%Area) with intensity normalization.

#### 2.4.5 Evaluation of tissue oxidative stress markers

The levels of GSH, MDA, and SOD activity in colonic tissue homogenates were determined using the same commercial assay kits and procedures as described for cell lysates in section 2.3.5, with tissue homogenates replacing cell lysates as the sample input. Data were normalized to total protein content.

#### 2.4.6 Enzyme-linked immunosorbent assay

The concentrations of Serum diamine oxidase (DAO) and D-lactate (D-LA) in serum were measured using ELISA kits (Fine Test, Wuhan, China; Wuhan Fine Biotech Co., Ltd., China) according to the manufacture’ instructions.

#### 2.4.7 RNA extraction and quantitative real-time PCR

Total RNA was extracted from colon tissues using the protocol described in Section 2.3.6. qPCR analysis and data normalization to GAPDH (2^−ΔΔCT^ method) were performed as above.

#### 2.4.8 Western blotting (tissue-based)

Western blot analysis of colon tissue proteins was performed following the identical protocol used for Ncm460 cells (Section 2.3.7).

#### 2.4.9 Intestinal flora 16S rRNA gene sequencing

Genomic DNA was extracted from the collected samples using the CTAB method (Wu et al., 2017), followed by quality assessment of DNA concentration and purity using a Nanodrop 2000 UV–Vis spectrophotometer (Thermo Fisher Scientific, USA) and 1% agarose gel electrophoresis. The V3-V4 hypervariable regions of bacterial 16S rDNA genes were amplified by PCR using specific primers 341F (5′-CCTAYGGGRBGCASCAG-3′) and 806R (5′-GGACTACNNGG GTATCTAAT-3′), with the resulting PCR products verified through 2% agarose gel electrophoresis and subsequently purified using the AxyPrep DNA Gel Extraction Kit (Axygen Biosciences, USA). After quantification and normalization, the DNA libraries were subjected to paired-end sequencing on an Illumina NovaSeq 6,000 platform (Illumina, USA). Functional potential of the microbial communities was predicted using PICRUSt2 (v2.5.2) based on the Greengenes reference database, using 16S rRNA gene sequence data. The predicted outcomes were mapped to the KEGG Pathway database (Level 3) to compare differences in microbial metabolic pathways across groups. All sequencing data were analyzed using R software (version 2.15.3), with 16S rRNA high-throughput sequencing of mouse colonic contents being performed by Wekemo Technology Group Co., Ltd. (Shenzhen, China).

### 2.5 Protein–protein docking analysis

The p38α MAPK crystal structure (PDB ID: 6TCA) was retrieved from the PDB database and preprocessed using PDBfixer to remove water molecules, original ligands, and irrelevant ions, with missing side-chain atoms subsequently added. The three-dimensional structure of the primary active component from AKK was obtained from PubChem, and its conformation was generated via AlphaFold2 prediction, yielding a model with high confidence (pLDDT > 90). Protein–protein molecular docking was performed using the HDOCK server.^1^ The top 10 models ranked by HDOCK confidence score were selected and further refined using FireDock. Finally, the optimized complex structures were visualized and analyzed with PyMOL 2.5.0 and LIGPLOT+ to characterize key intermolecular interactions, including hydrogen bonds, hydrophobic contacts, and the overall binding interface.

### 2.6 Statistical analysis

Prior to intergroup comparisons, normality of all data was assessed using the Shapiro–Wilk test, and homogeneity of variances was evaluated using Levene’s test. For *in vitro* experiments, *n* represents the number of independent biological replicates, where each replicate denotes cells from a different passage or an independent culture. For *in vivo* studies, *n* refers to the number of animals per group. Data are expressed as mean ± standard error of the mean (SEM). All data visualization were performed using GraphPad Prism 9.5.0 (GraphPad Software Inc., San Diego, CA, USA). For multiple group comparisons, one-way ANOVA followed by Tukey’s *post hoc* test was uesed for normally distributed data with equal variance, while the Kruskal-Wallis test with Dunn’s multiple comparisons correction was applied for non-normally distributed data. All analyses were conducted using SPSS 27.0 (IBM Corp., Armonk, NY, USA). A two-tailed *p*-value < 0.05 was considered statistically significant.

## 3 Results

### 3.1 AKK inhibits the phosphorylation of p38α MAPK *in vitro*

**Figure 2:**
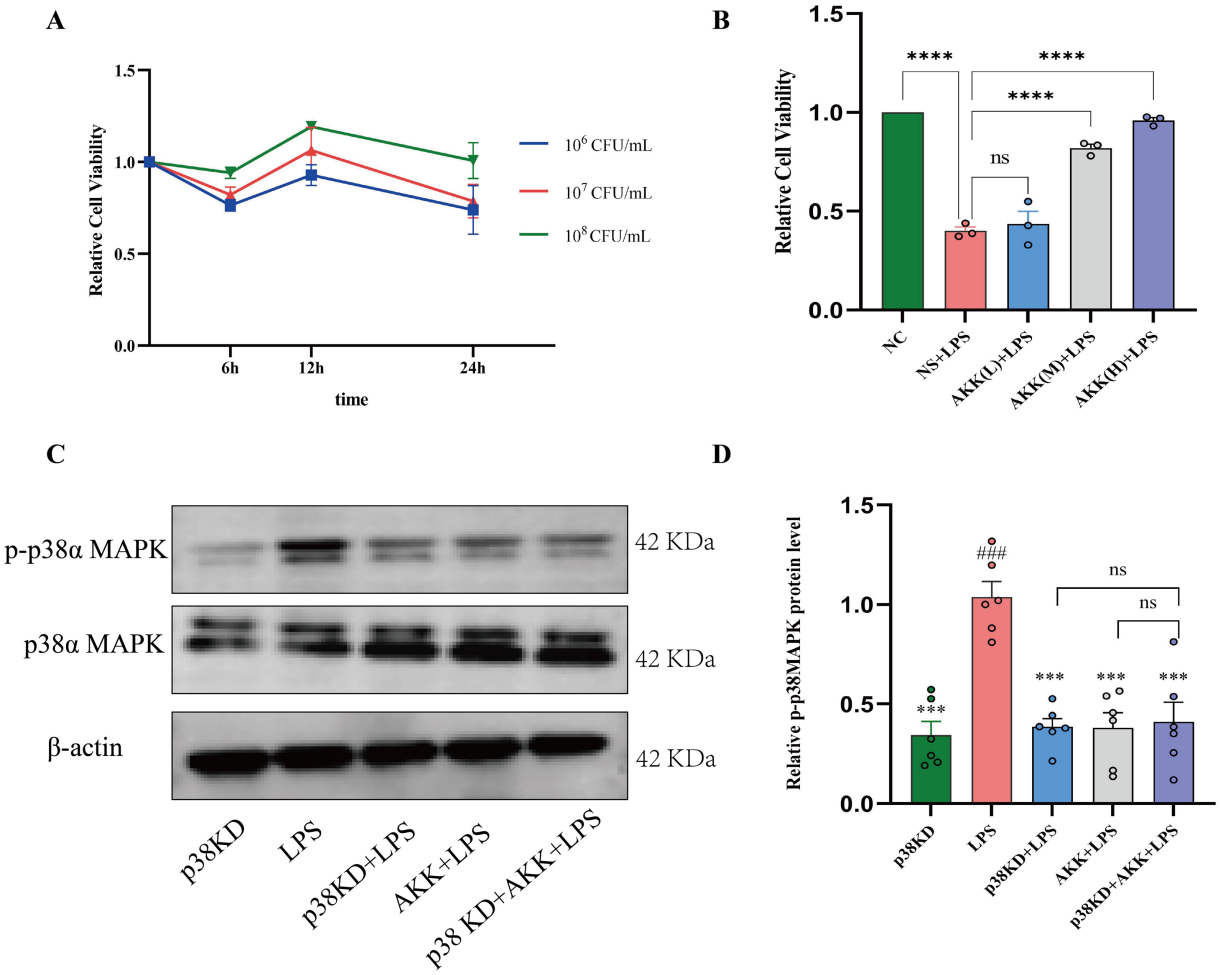
AKK directly inhibits phosphorylation and activation of p38α MAPK in human colonic epithelial cells. In the LPS-induced injury model of Ncm460 cells: **(A)** Identification of the optimal concentration and treatment time for AKK via CCK-8 assay. **(B)** AKK reverses the LPS-induced suppression of cell viability. Data are normalized to the control group (set as 1.0). **(C)** Representative western blot images of p38α MAPK and phosphorylated p38α MAPK (p-p38α MAPK). Blots for p38α MAPK and p-p38α MAPK were obtained from the same membrane. **(D)** Quantitative analysis of total p-p38α MAPK protein expression levels. *n* = 6 independent biological replicates. All data are presented as mean ± SEM. #*p* < 0.05, ###*p* < 0.001, ####*p* < 0.0001 versus p38KD group. *p < 0.05, **p > *p* < 0.01, ***p < 0.001, ****p < 0.0001 versus LPS group.

To investigate whether AKK exerts a protective effect through the regulation of the p38α MAPK signaling pathway, we initially established a LPS-induced injury model in human colon epithelial cells (Ncm460). Subsequent CCK-8 assay results indicated that a concentration of 10^8^ CFU/mL represented the optimal dose for AKK intervention in vitro (Figures 2A,B).

Having confirmed LPS-induced activation of p38α MAPK signaling, we further employed a genetic knockdown model to validate the necessity of this pathway in AKK-mediated protection. Western blot analysis revealed the following: (1) p38α knockdown (KD) + LPS cells showed significantly reduced p-p38α MAPK levels compared to the LPS group, confirming effective suppression of pathway activation; (2) AKK + LPS treatment markedly decreased p-p38α MAPK relative to the LPS model, indicating that AKK inhibits p38α MAPK phosphorylation; (3) most importantly, in p38α KD + LPS + AKK cells, p-p38α MAPK levels did not differ significantly from those in p38α KD + LPS cells, demonstrating that AKK fails to further suppress phosphorylation when p38α is knocked down; (4) p38α KD alone did not affect basal cell status, confirming specific knockdown efficiency. Together, these results provide genetic evidence that p38α MAPK is necessary for AKK-mediated suppression of p38 phosphorylation and subsequent protective effects (Figures 2C,D).

### 3.2 AKK alleviates oxidative stress via inhibition of p38α MAPK

**Figure 3:**
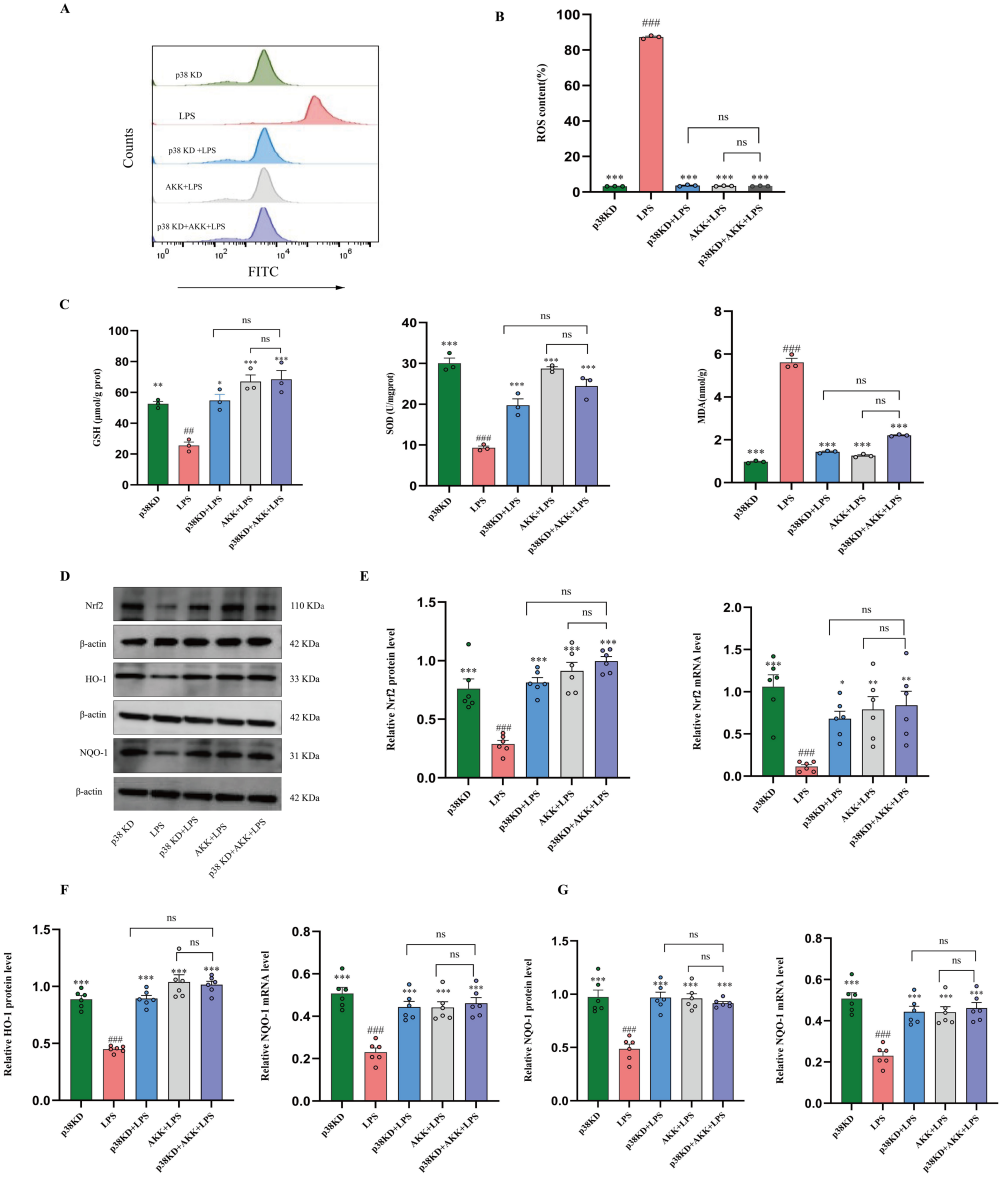
Inhibition of p38α MAPK alleviates oxidative stress in human colonic epithelial cells by activating the Nrf2 pathway. In the LPS-induced injury model of Ncm460 cells: **(A,B)** Representative flow cytometry scatter plots and quantitative analysis of intracellular ROS levels. **(C)** Levels of cellular oxidative stress markers (GSH, SOD, MDA). **(D)** Representative western blot images of Nrf2 and its downstream targets HO-1 and NQO1. The representative western blot images presented here are accompanied by the corresponding internal control detected on the same membrane. **(E–G)** Quantitative analysis of protein and mRNA expression levels of Nrf2 **(E)**, HO-1 **(F)**, and NQO1 **(G)**. *n* = 6 independent biological replicates. All data are presented as mean ± SEM. #*p* < 0.05, ###*p* < 0.001, ####*p* < 0.0001 versus p38KD group. *p < 0.05, **p < 0.01, ***p < 0.001, ****p < 0.0001 versus LPS group.

Having established that AKK functions through p38α MAPK inhibition, we assessed its downstream functional consequence on cellular oxidative stress. Flow cytometry revealed that LPS (10 μg/mL, 24 h) significantly increased ROS levels compared to the NC group (*p* < 0.001). This elevation was effectively rescued by AKK co-treatment, which restored ROS to near-baseline levels (*p* < 0.001). Crucially, in p38α KD cells, the LPS-induced ROS burst was substantially attenuated. Under this condition of p38α knockdown, AKK intervention failed to further reduce ROS. These results genetically demonstrate that the antioxidant effect of AKK is entirely dependent on its suppression of the p38α MAPK pathway (Figures 3A,B).

Having demonstrated AKK’s capacity to suppress intracellular ROS, we further investigated its effects on key markers of oxidative damage and the cellular antioxidant defense system. Colorimetric assays revealed that, compared with the p38 KD group, the LPS group exhibited significantly lower levels of GSH and SOD, along with a marked increase in MDA content (*p* < 0.01 or *p* < 0.001). In wild-type cells, AKK treatment effectively reversed these changes, leading to significant improvements in all three oxidative parameters (AKK + LPS vs. LPS, *p* < 0.001). Notably, the p38α KD + LPS group displayed substantially reduced oxidative stress relative to the LPS group. Crucially, under p38 knockdown conditions, AKK administration failed to produce any additional improvement in these oxidative markers (p38α KD + AKK + LPS vs. p38α KD + LPS, *p* > 0.05). Collectively, these findings demonstrate that AKK regulates systemic cellular redox homeostasis in a manner dependent on p38α MAPK signaling (Figure 3C).

Having established that AKK regulates oxidative stress via the p38α MAPK pathway, we further investigated its influence on the core antioxidant transcriptional program mediated by the Nrf2 signaling axis. Western blot and qPCR analyses revealed that, compared to the p38α KD group, LPS stimulation markedly suppressed nuclear translocation of Nrf2 and downregulated the expression of its downstream targets—heme oxygenase-1 (HO-1) and NAD(P)H quinone oxidoreductase 1 (NQO1). In wild-type cells, AKK treatment effectively reversed these inhibitory effects. However, in the p38α KD + LPS group, although there was partial restoration of Nrf2 pathway activity relative to the LPS group, AKK failed to further enhance the expression levels of Nrf2, HO-1, or NQO1 under p38-deficient conditions. These results demonstrate that AKK activates the Nrf2 signaling pathway in a p38α MAPK-dependent manner, thereby modulating cellular antioxidant defense mechanisms (Figures 3D–G).

### 3.3 AKK promotes epithelial migration and tight junction expression via p38α MAPK inhibition

**Figure 4:**
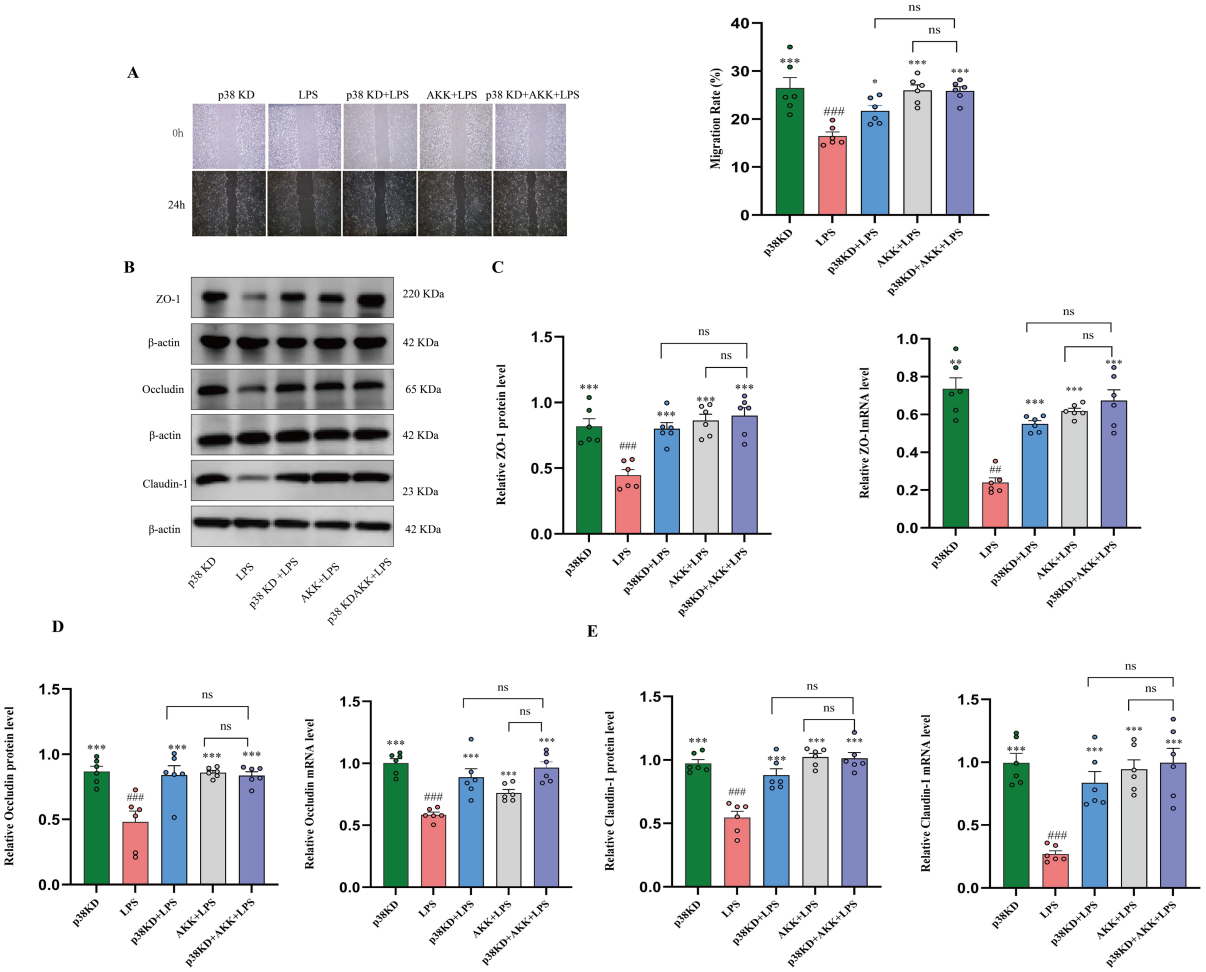
AKK enhances migration and tight junction integrity in human colonic epithelial cells in a p38α MAPK-dependent manner. In the LPS-induced injury model of Ncm460 cells: **(A)** Representative images and quantitative analysis of wound closure rate in the scratch assay. **(B)** Representative western blot images of tight junction proteins. The representative western blot images presented here are accompanied by the corresponding internal control detected on the same membrane. **(C–E)** Quantitative analysis of protein and mRNA expression levels of ZO-1 **(C)**, Occludin **(D)**, and Claudin-1 **(E)**. *n* = 6 independent biological replicates. #*p* < 0.05, ###*p* < 0.001, ####*p* < 0.0001 versus p38KD group. *p < 0.05, **p < 0.01, ***p < 0.001, ****p < 0.0001 versus LPS group.

Having established the critical roles of both AKK and the p38α MAPK pathway in alleviating oxidative stress, we further examined their effects on the functional integrity of the intestinal barrier. A cell scratch assay revealed that, compared with the p38 KD group, LPS stimulation significantly impaired cell migration. In wild-type cells, AKK treatment markedly promoted wound closure. Notably, p38α MAPK knockdown alone effectively mitigated the LPS-induced suppression of cell migration (Figure 4A).

At the molecular level, Western blot and qPCR analyses of tight junction proteins revealed that LPS stimulation significantly downregulated the expression of ZO-1, occludin, and claudin. This downregulation was effectively reversed by AKK intervention in control cells. Notably, in p38α-knockdown cells, TJs protein expression was already stabilized due to genetic ablation, and no statistically significant additional improvement was observed with AKK treatment. Collectively, our findings—from cellular functional assays to molecular expression analyses—demonstrate that inhibition of the p38α MAPK signaling pathway is the primary and essential mechanism underlying AKK-mediated epithelial repair and barrier restoration (Figures 4B–E).

### 3.4 AKK attenuates ABX/LPS-induced gut epithelial damage in a concentration-dependent manner

Based on the aforementioned *in vitro* findings that AKK exerts its protective effects through inhibition of the p38α MAPK pathway, we further sought to validate its therapeutic potential and dose–response effects at the whole-animal level. Therefore, we established a murine model of gut barrier dysfunction induced by a combination of antibiotics and LPS, simulating clinical conditions in which antibiotic-induced dysbiosis predisposes to secondary Gram-negative bacterial infections and subsequent LPS translocation. In the model group, mice exhibited transient weight loss as early as day 3 post-induction, accompanied by reduced food and water intake. Following LPS challenge on days 9–10, body weight declined most markedly, with severe diarrhea, hematochezia, and systemic symptoms including lethargy, piloerection, and decreased mobility. DAI scores remained significantly elevated throughout the experimental period. In contrast, AKK-treated mice demonstrated substantial attenuation of weight loss, restoration of normal feeding and hydration behaviors, and dose-dependent improvements in diarrhea, hematochezia, and DAI scores relative to the model group (Figures 1B,C).

Colon length, a macroscopic indicator of intestinal inflammation, was markedly reduced in ABX/LPS-challenged mice, reflecting severe colitis. Histological evaluation revealed extensive inflammatory cell infiltration and significant architectural disruption of crypts and villi in both the colon and ileum. AKK intervention exerted a protective effect, which was dose-dependent, with the high-dose AKK group demonstrating the most pronounced protection, significantly reducing colonic inflammation scores and promoting mucosal repair. Moreover, morphometric analysis of the ileum—measuring villus height, crypt depth, and the villus height-to-crypt depth ratio (V/C)—demonstrated that AKK effectively preserved intestinal absorptive architecture (Figures 1D–I).

**Figure 5:**
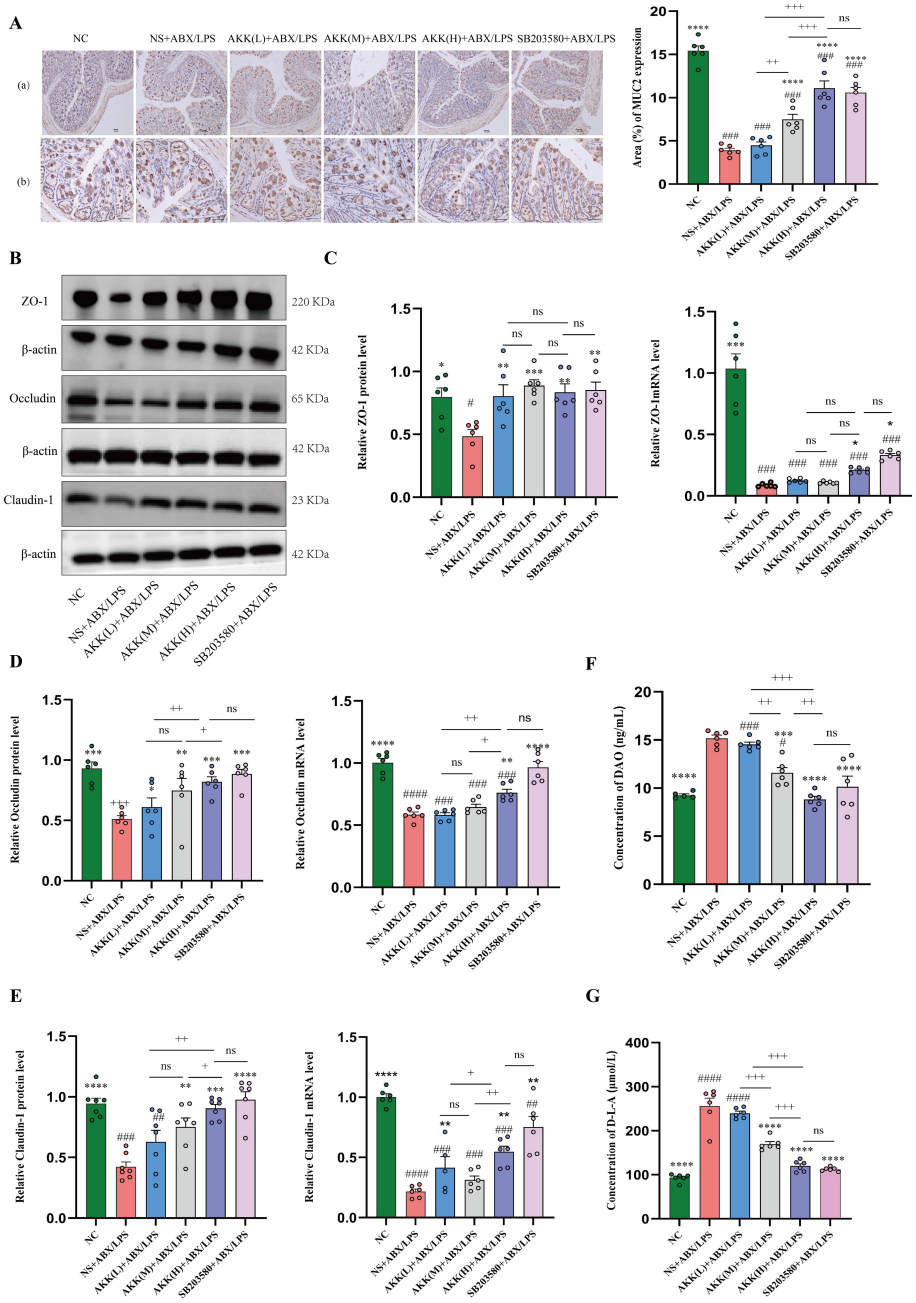
*Akk* restores the intestinal barrier by upregulating MUC2 and tight junction expression in a p38α MAPK-dependent manner. **(A)** Modulation of the mucus barrier by *Akk* and the p38α MAPK inhibitor (SB203580): representative IHC images of MUC2 and quantitative analysis. **(B)** Representative western blot images of tight junction proteins across groups. The representative western blot images presented here are accompanied by the corresponding internal control detected on the same membrane. **(C–E)** Quantitative analysis of protein and mRNA expression levels of ZO-1 **(C)**, occludin **(D)**, and claudin-1 **(E)**. **(F,G)** Serum levels of intestinal barrier damage markers, DAO activity, and D-LA concentration. *n* = 6 independent biological replicates. All data are presented as mean ± SEM. #*p* < 0.05, ###*p* < 0.001, ####*p* < 0.0001 versus NC group; *p < 0.05, **p < 0.01, ***p < 0.001, ****p < 0.0001 versus NS + ABX/LPS group, +*p* < 0.05, ++*p* < 0.01, +++*p* < 0.01, +++*p* < 0.001, +++*p* < 0.0001 versus the AKK-treated group.

To investigate whether AKK could restore the intestinal mucus barrier impaired by ABX/LPS treatment, we performed MUC2 immunohistochemical staining. Exposure to ABX/LPS markedly decreased MUC2 protein expression in the colonic mucosa (*p* < 0.001). AKK administration reversed this suppression in a concentration-dependent manner. Notably, the high-dose AKK group showed the most pronounced recovery of MUC2 expression, with the positive signal area nearly restored to control levels and a significant increase in goblet cell regeneration (*p* < 0.001) (Figure 5A).

Western blot analysis showed that the ABX/LPS challenge markedly decreased the protein levels of key tight junction proteins—ZO-1, Occludin, and Claudin-1—in colonic tissue. AKK intervention restored the levels of these proteins. Specifically, the restorative effects on Occludin and Claudin-1 were dose-dependent, with the high-dose AKK group demonstrating the most pronounced recovery. In contrast, the restoration of ZO-1 did not differ significantly among the AKK dose groups. These protein-level changes were paralleled at the transcriptional level by qPCR analysis of the corresponding genes. Collectively, these findings indicate that AKK effectively promotes the expression of tight junction proteins, with a dose-dependent enhancement specifically observed for Occludin and Claudin-1 (Figures 5B–E).

To assess intestinal barrier function, serum levels of DAO and D-LA were measured. The ABX/LPS challenge significantly elevated serum DAO activity and D-LA concentration, indicating markedly increased intestinal permeability. AKK intervention effectively reversed this effect in a dose-dependent manner, with the high-dose AKK group exhibiting the most pronounced improvement, restoring both markers to near-normal levels (Figures 5F,G).

### 3.5 Antibiotic and LPS administration induces intestinal oxidative stress

**Figure 6:**
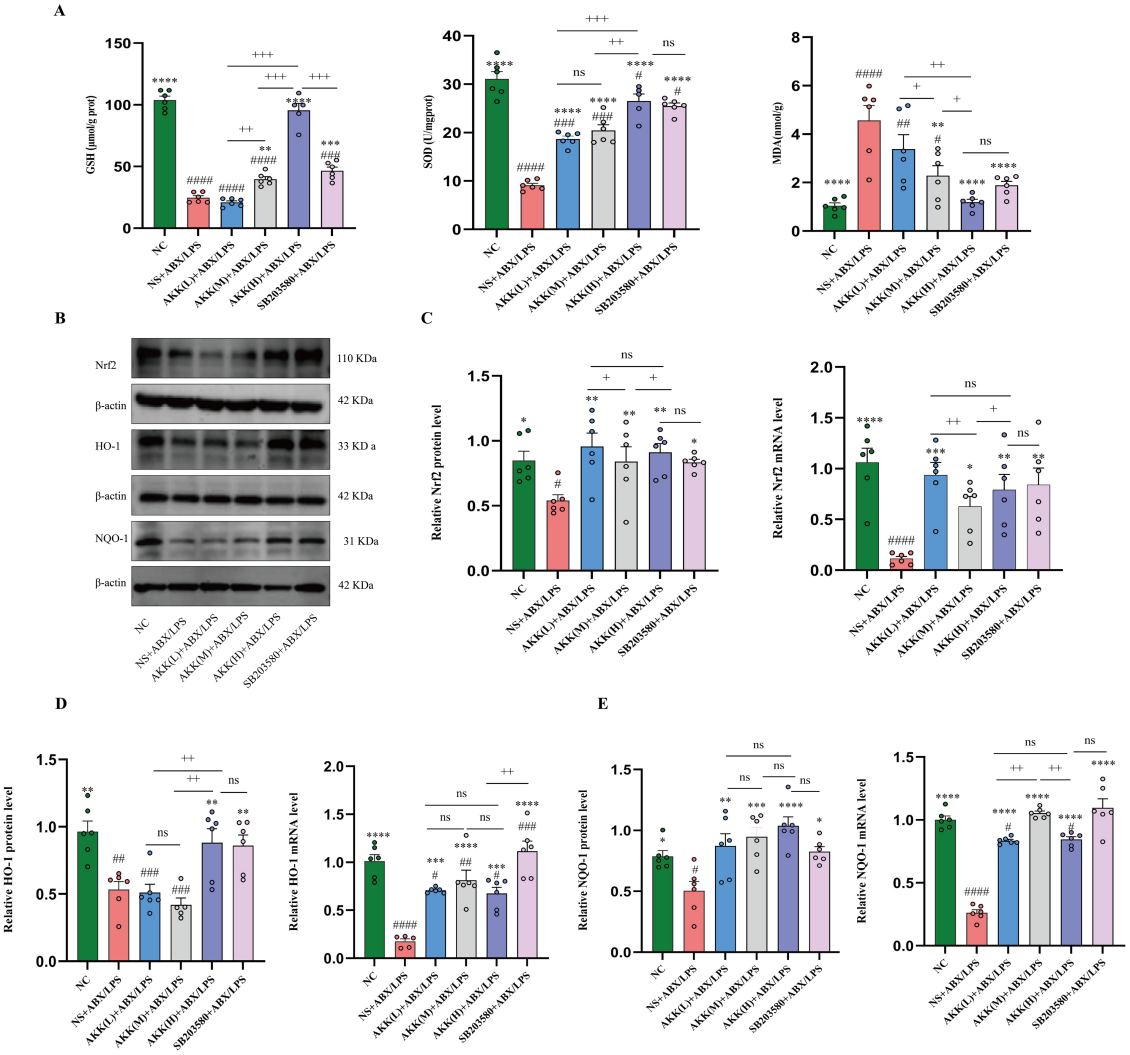
AKK alleviates intestinal oxidative stress by activating the Nrf2 signaling pathway through inhibition of p38α MAPK. **(A)** Levels of oxidative stress markers in colonic tissue: GSH, SOD, and MDA **(B)** Representative western blot images of Nrf2 and its downstream targets, HO-1 and NQO1. The representative western blot images presented here are accompanied by the corresponding internal control detected on the same membrane. *n* = 6 independent biological replicates. **(C–E)** Quantitative analysis of protein and mRNA expression levels of Nrf2 **(C)**, HO-1 **(D)**, and NQO1 **(E)**. *n* = 6 independent biological replicates. All data are presented as mean ± SEM. #*p* < 0.05, ###*p* < 0.001, ####*p* < 0.0001 versus NC group; *p < 0.05, **p < 0.01, ***p < 0.001, ****p < 0.0001 versus NS + ABX/LPS group, +*p* < 0.05, ++*p* < 0.01, +++*p* < 0.01, +++*p* < 0.001, +++*p* < 0.0001 versus the A-treated group.

Given the pivotal role of oxidative stress in driving intestinal pathology (Sui et al., 2024; Liu et al., 2020), we hypothesized that it serves as a central mechanism underlying ABX/LPS-induced gut injury. As shown in Figure 6A, the ABX/LPS challenge induced significant oxidative stress, as evidenced by markedly reduced SOD activity and GSH levels, along with a substantial increase in MDA, a lipid peroxidation product. AKK treatment dose-dependently reversed these alterations in oxidative stress markers. Notably, high-dose AKK administration restored SOD and GSH levels to near baseline and reduced MDA content to levels comparable to those in the NC group.

We subsequently hypothesized that the observed amelioration of oxidative stress might be mediated through the Nrf2 antioxidant pathway. To investigate whether AKK is related to the activation of the Nrf2-mediated antioxidant pathway, we measured the expression levels of key molecules in this pathway. Western blot analysis revealed that ABX/LPS stimulation significantly reduced the levels of nuclear Nrf2, HO-1, and NQO1 in colon tissues. AKK intervention differentially restored the expression of these proteins. Notably, nuclear Nrf2 was significantly restored across all AKK dose groups, with statistically significant differences in the extent of recovery observed between specific concentration pairs (low vs. medium, and medium vs. high). The protein level of HO-1 was most prominently restored in the high-dose group. Although no statistically significant differences were detected among the AKK groups for NQO1 protein, the most evident restorative trend was observed in the high-dose group. At the transcriptional level, qPCR analysis confirmed that ABX/LPS exposure markedly downregulated mRNA expression of Nrf2, HO-1, and NQO1, an effect that was effectively attenuated by AKK treatment across all tested doses (Figures 6B–E).

### 3.6 From structural prediction to experimental validation: evidence for AKK targeting p38α MAPK

**Figure 7:**
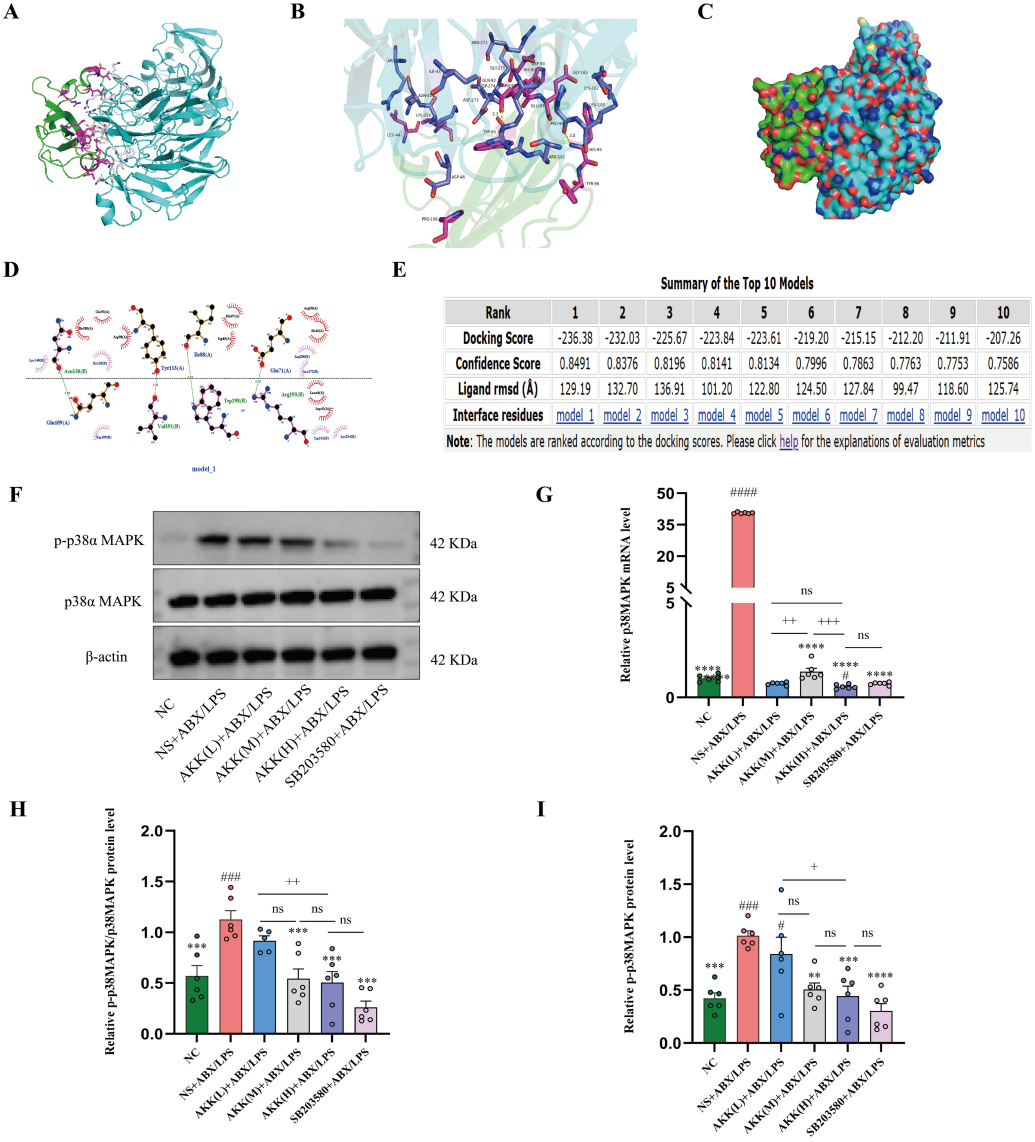
Molecular docking predicts direct binding of AKK to p38α MAPK and validation in a murine model. Exploratory molecular docking results demonstrating strong binding between AKK and p38α MAPK: **(A)** Detailed view of the binding mode, with a binding free energy of −7.0 kcal·mol^−1^; **(B)** 3D representation of binding site interactions (yellow dashed lines indicate hydrogen bonds); **(C)** Schematic 3D structure of the complex; **(D)** 2D diagram of binding site interactions. **(E)** Top ten ranked binding conformations from docking screening. **(F–I)** Experimental validation in an ABX/LPS-induced mouse intestinal injury model: **(F)** Representative western blot images of p38α MAPK and p-p38α MAPK in colonic tissue; Blots for p38α MAPK and p-p38α MAPK were obtained from the same membrane. **(G)** p38α MAPK mRNA expression levels in the colon; **(H)** p-p38α MAPK/p38α MAPK ratio; **(I)** Total p-p38α MAPK protein levels. *n* = 6 independent biological replicates. All data are presented as mean ± SEM. #*p* < 0.05, ###*p* < 0.001, ####*p* < 0.0001 versus NC group; *p < 0.05, **p < 0.01, ***p < 0.001, ****p < 0.0001 versus NS + ABX/LPS group, +*p* < 0.05, ++*p* < 0.01, +++ < 0.01, +++*p* < 0.001, +++*p* < 0.0001 versus the AKK-treated group.

To elucidate the upstream mechanism through which AKK activates Nrf2, we hypothesized that AKK modulates the Nrf2-mediated cellular defense response by targeting the p38α MAPK pathway—a key regulator of oxidative stress signaling. To investigate the potential interaction between key active components of AKK and p38α MAPK, an exploratory molecular docking analysis was conducted in this study. The crystal structure of the core p38α MAPK protein was retrieved from the PDB database, and the three-dimensional conformation of the primary bioactive component of AKK was generated in silico. Protein-ligand docking simulations were performed using HDOCK, revealing stable binding conformations with a binding energy < −7.0 kcal·mol^−1^; these interactions were further assessed using PDBePISA for thermodynamic and interface stability validation. The binding mode was visualized using PyMOL and LigPlot (Figures 7A–E), Computational simulation results providing structural evidence suggestive of direct interaction between AKK and p38α MAPK. To verify this calculation prediction, we obtained strong support through *in vivo* experiments: The p-p38α/p38α MAPK ratio was significantly elevated in the NS + ABX/LPS group. AKK treatment not only markedly reduced the phosphorylation level of p38α MAPK but also downregulated the expression of p38α mRNA. Notably, although mRNA levels decreased, total p38α MAPK protein levels did not differ significantly among the groups, suggesting possible compensatory regulation at the translational or protein stability level. Collectively, these integrated computational and experimental findings indicate that AKK may exert its effects through direct binding and inhibition of p38α MAPK phosphorylation, coupled with modulation of its transcriptional and post-transcriptional homeostasis, thereby multilaterally suppressing its pathological activation (Figures 7F–I).

### 3.7 Pharmacological inhibition of p38α MAPK restores intestinal barrier integrity impaired by antibiotics and LPS

To establish the necessity of the p38α MAPK pathway in AKK-conferred protection, we included a parallel intervention group treated with the p38α MAPK inhibitor SB203580. Notably, inhibition of p38α MAPK signaling completely recapitulated the protective effects of high-dose AKK: both the inhibitor and high-dose AKK groups significantly ameliorated body weight loss, lowered the DAI score, and reversed colon shortening compared to the model group (Figures 1B–E).

Histopathological evaluation demonstrated that the p38α MAPK inhibitor group achieved more substantial restorative outcomes compared to the high-dose AKK group, as evidenced by reduced colonic inflammation scores and better preservation and recovery of ileal villus structure (Figures 1D–I). Notably, while both treatments significantly enhanced MUC2 expression—an essential component of the intestinal mucus barrier—the AKK group exhibited a slightly greater upregulation (Figure 5A). Collectively, these results indicate that inhibition of p38α MAPK is sufficient to recapitulate the intestinal barrier protection conferred by AKK. The marginally superior outcomes with AKK treatment in certain parameters, such as MUC2 expression, are likely attributable to its dual capacity to directly modulate host signaling and indirectly reshape the gut microbial ecosystem, thereby fostering a microenvironment more conducive to mucosal healing.

Both the p38α MAPK inhibitor and high-dose AKK markedly enhanced intestinal barrier integrity, as evidenced by increased expression of tight junction proteins and reduced gut permeability. Western blot and qPCR analyses revealed that the p38α MAPK inhibitor upregulated the expression of Occludin, Claudin, and ZO-1 to levels comparable to, or slightly exceeding, those achieved with high-dose AKK (Figures 5B–E). Consistently, serum markers of intestinal permeability—DAO and D-LA—were significantly reduced following p38α MAPK inhibition, to an extent similar to that observed with high-dose AKK treatment (Figures 5F,G). Collectively, these findings demonstrate that inhibition of the p38α MAPK pathway alone is sufficient to reproduce the core barrier-restorative effects conferred by high-dose AKK.

### 3.8 Targeting p38α MAPK signaling alleviates gut oxidative stress triggered by antibiotic and LPS challenge

Having established the role of p38α MAPK in regulating intestinal barrier function, we further explored its critical involvement in oxidative stress. Administration of a p38α MAPK inhibitor markedly attenuated oxidative stress, as evidenced by the restoration of SOD activity and GSH levels, along with a significant reduction in MDA content. The magnitude of this protective effect was comparable to that observed with high-dose AKK treatment (Figure 6A). These findings demonstrate that activation of p38α MAPK is a key contributor to intestinal oxidative injury, and pharmacological inhibition of this pathway alone is sufficient to recapitulate the primary antioxidant effects of AKK.

To delineate the downstream mechanism by which p38α MAPK regulates oxidative stress, we examined the Nrf2 signaling pathway. Inhibition of p38α MAPK robustly activated Nrf2 signaling, demonstrated by enhanced nuclear translocation of Nrf2 and coordinated upregulation of its target genes, HO-1 and NQO1, at both the protein and mRNA levels (Figures 6B–E). Notably, this intervention induced a more potent transcriptional activation of these antioxidant genes than AKK treatment. These findings establish that p38α MAPK inhibition orchestrates endogenous antioxidant defense by activating the Nrf2 pathway.

### 3.9 AKK modulates gut microbiota composition and function in association with host barrier restoration

Having established that AKK directly modulates host barrier function and oxidative stress via the p38*α* MAPK–Nrf2 axis, we further investigated whether these effects were accompanied by alterations in the intestinal microbial ecosystem. As a constituent member of the gut microbiota, AKK may confer benefits not only through direct host interactions but also by remodeling the overall microbial community structure, thereby providing additional supportive evidence for its protective effects.

**Figure 8:**
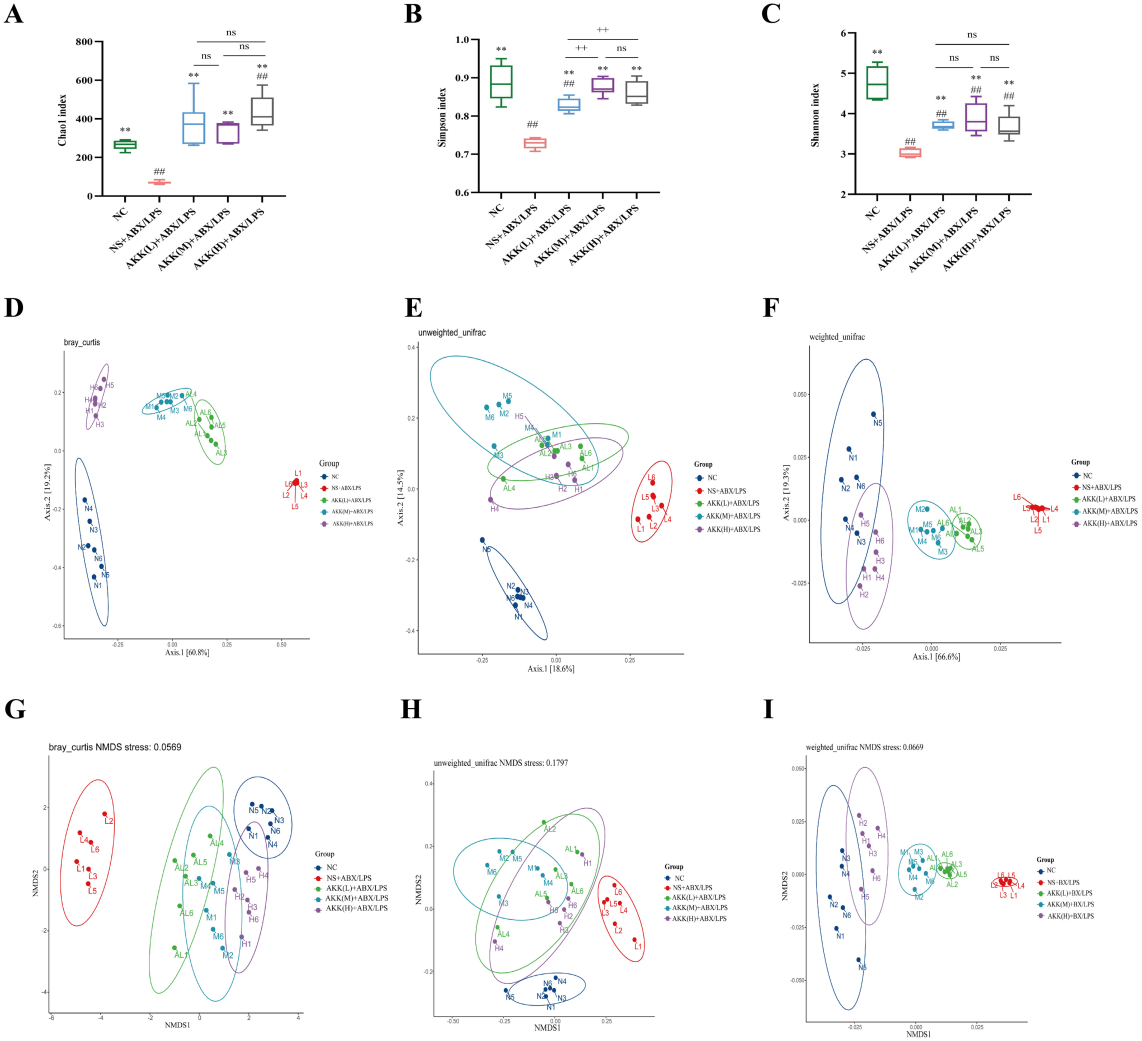
*Akk* reverses antibiotic and LPS-induced gut microbiota dysbiosis in a dose-dependent manner. **(A-C)** Alpha diversity analysis. The Chao1 richness index, Simpson index, and Shannon diversity index are shown for different experimental groups, representing community richness, dominance, and diversity, respectively. **(D-F)** Beta diversity analysis (Principal Coordinates Analysis, PCoA). Structural differences in microbial communities among groups are visualized using Principal Coordinates Analysis based on Bray-Curtis, weighted UniFrac, and unweighted UniFrac distance metrics. **(G-I)** Beta diversity analysis (Non-metric Multidimensional Scaling, NMDS). Group differences are further visualized using Non-metric Multidimensional Scaling ordination plots based on the same distance metrics (Bray-Curtis, weighted and unweighted UniFrac) as in **(D-F)**. The corresponding stress value is indicated for each plot.

To assess bacterial community composition in colonic fecal samples from each mouse group, Illumina NovaSeq 6000 sequencing was performed, and microbiota alpha diversity was evaluated based on the resulting data (Figures 8A–C). The NS + ABX/LPS group exhibited significantly lower Chao1, Simpson, and Shannon indices compared to the NC group and all AKK-treated groups (*p* < 0.01), indicating that combined antibiotic and LPS treatment led to a marked reduction in microbial diversity. Although both the Chao1 and Shannon indices exhibited dose-dependent increasing trends across the AKK treatment groups, no statistically significant differences were observed among the different dosage groups (*p* > 0.05). However, both indices in the high-dose AKK group were significantly higher than those in the NC group (*p* < 0.05). The pattern for the Simpson index was distinct: significant differences were detected between the low-dose AKK group and the NC group, as well as between the low-dose and both the medium- and high-dose AKK groups (*p* < 0.05), whereas no significant difference was found between the medium- and high-dose AKK groups. In summary, while α-diversity did not differ significantly among the various AKK intervention groups (*p* > 0.05), its modulation by AKK varied depending on the specific diversity index and the groups being compared.

We further evaluated the beta diversity of the gut microbiota across groups using Bray–Curtis, weighted UniFrac, and unweighted UniFrac distance metrics, visualized through Principal Coordinates Analysis (PCoA) and Non-Metric Multidimensional Scaling (NMDS), with statistical validation provided by ANOSIM and PERMANOVA. All dimensionality reduction approaches consistently demonstrated that samples from the NS + ABX/LPS group formed a distinct and tightly clustered profile, clearly separated from all other groups. In contrast, samples from the AKK treatment groups exhibited overlapping distributions arranged in a concentration-dependent gradient, progressively shifting toward the NC group. These findings indicate that AKK treatment ameliorates ABX/LPS-induced disruption of the microbial community in a dose-dependent manner, restoring a community structure closely resembling that of the NC group, with high structural similarity observed across the AKK-treated groups (Figures 8D–I).

Paired comparisons across all analytical methods yielded highly consistent results, revealing statistically significant differences in microbial communities between the NS + ABX/LPS group and all other groups (*p* < 0.01). The ANOSIM R-values approached or reached 1, while the PERMANOVA pseudo-F statistics were remarkably high (ranging from 4.60 to 140.40), indicating that combined antibiotic and LPS treatment induced a profound reorganization of the microbial community. Between-group differences far exceeded within-group variations, resulting in a distinct microbial ecotype. As the concentration of AKK treatment increased, the pseudo-*F* values relative to the NC group gradually declined, and the ANOSIM R-values progressively moved away from 1, suggesting a reduction in intergroup dissimilarity and a convergence of community composition. These results demonstrate that AKK facilitates a dose-dependent restoration of the microbial community toward a normative state.

**Figure 9:**
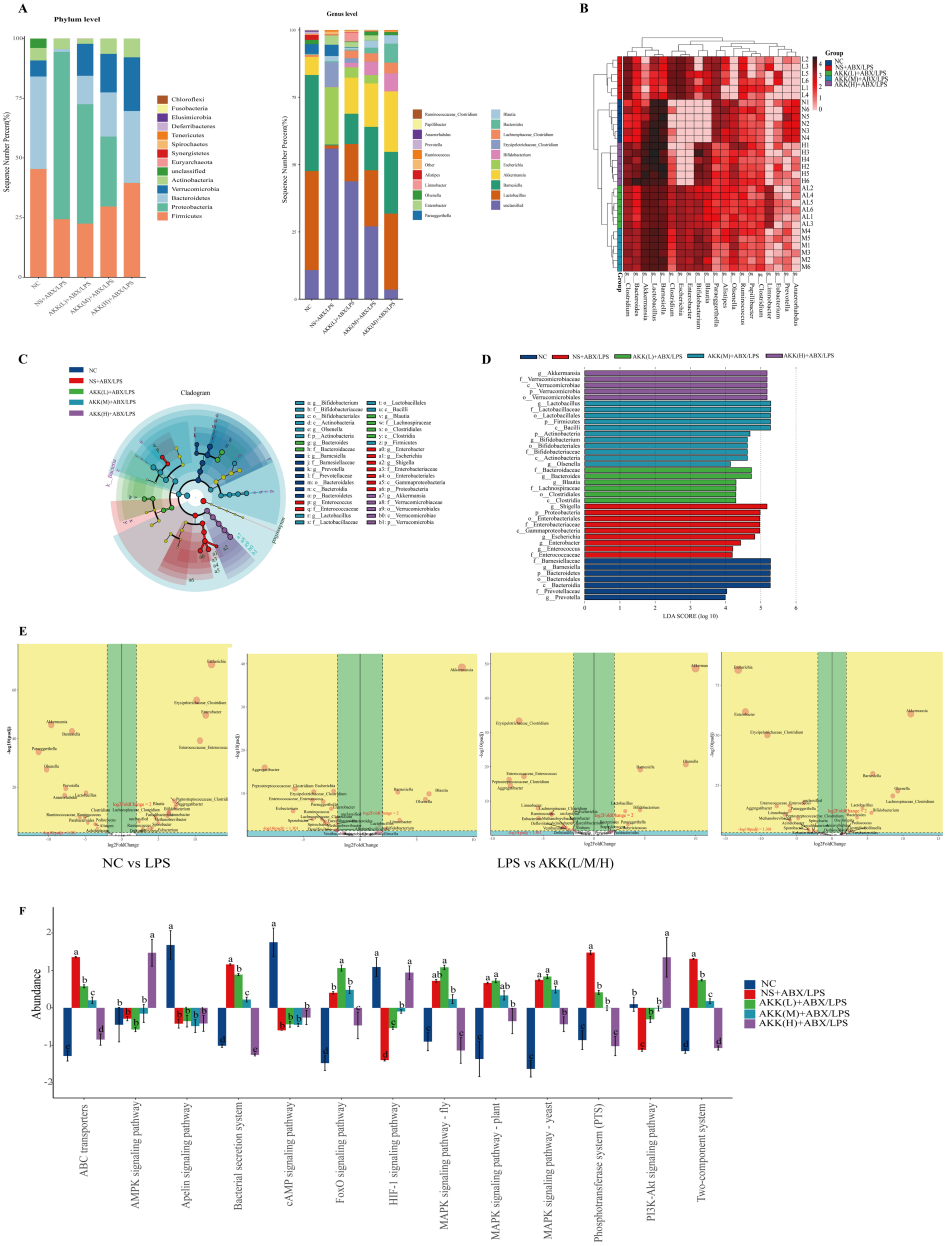
AKK restructures the gut microbiota and modulates its functional potential. **(A)** Microbial community composition at the phylum and genus levels, demonstrating the dose-dependent modulation by AKK. **(B)** Heatmap and hierarchical clustering at the genus level, revealing the co-abundance relationships between samples and species. **(C)** LEfSe cladogram illustrating the phylogenetic distribution of group-specific biomarkers. **(D)** LDA score histogram quantifying the most differentially abundant taxa in each group. **(E)** DESeq2 volcano plot identifying significantly enriched beneficial genera and conditional pathogens between the NC and NS + ABX/LPS groups. **(F)** Functional prediction heatmap based on PICRUSt2, showing the modulation of microbial functional profiles by AKK, particularly pathways related to material transport and signal transduction.

Following the confirmation of significant intergroup differences in beta diversity, we further examined the structural alterations in the microbial community at both the phylum and genus levels (Figures 9A,B). At the phylum level, the NS + ABX/LPS group exhibited a marked expansion of *Proteobacteria* (64.4%), along with significant reductions in *Bacteroidota* and *Verrucomicrobia* (*p* < 0.05). Notably, AKK treatment reversed these dysbiotic shifts in a dose-dependent manner, effectively suppressing the overgrowth of *Proteobacteria* and facilitating the restoration of beneficial bacterial phyla. At the genus level, further structural analysis revealed that the abundances of beneficial genera—including *Lactobacillus, Bifidobacterium, Blautia*, and *Akkermansia*—were significantly reduced in the NS + ABX/LPS group. In contrast, conditional pathogenic genera such as *Escherichia, Enterobacter, Paraeggerthella,* and *Erysipelatoclostridium* were markedly increased (*p* < 0.05). AKK treatment effectively suppressed the proliferation of these opportunistic pathogens and promoted a dose-dependent recovery of beneficial genera, thereby shifting the microbial community structure toward that observed in the NC group. Cluster heatmap analysis revealed that NS + ABX/LPS samples clustered distinctly from the NC and AKK treatment groups, which grouped together in a separate branch, highlighting marked intergroup divergence and strong intragroup homogeneity. Species-level clustering indicated a clear antagonistic pattern between pathogenic and beneficial bacterial genera. Collectively, AKK effectively ameliorates ABX/LPS-induced gut microbiota dysbiosis by suppressing pathogenic bacterial growth and enhancing the abundance of beneficial microbial taxa.

To comprehensively resolve intergroup differences at the genus level, we conducted an integrated analysis using LEfSe (LDA Effect Size) and DESeq2 for in-depth characterization and visualization (Figures 9C–E). The results revealed marked differences in microbial community composition between the NC and NS + ABX/LPS groups. Cladogram analysis showed that taxa enriched in the NS + ABX/LPS group were predominantly clustered within the Proteobacteria lineage, whereas increased AKK supplementation shifted the microbial profile toward a configuration more similar to that of the NC group, suggesting a restorative effect. LDA analysis further identified distinct biomarker taxa for each group: the NC group was characterized by *Bacteroidota, Barnesiella*, and *Prevotella*; the NS + ABX/LPS group was dominated by opportunistic pathogens from the Proteobacteria phylum (LDA > 4); and the low-, medium-, and high-dose AKK groups were associated with *Lachnospiraceae* and *Blauti*a, beneficial members of *Firmicutes* and *Actinobacteria* (e.g., *Lactobacillus, Bifidobacterium*), and *Verrucomicrobiota* and *Akkermansia*, respectively. Volcano plot analysis based on DESeq2 (threshold: |log_2_FC| > 2) revealed that the NC group was significantly enriched in beneficial genera such as *Lactobacillus, Bifidobacterium, Akkermansia, Olsenella*, and *Barnesiel*la, whereas the NS + ABX/LPS group exhibited marked enrichment in opportunistic pathogens including *Escherichia, Erysipelatoclostridium, Enterococcus*, and *Enterobacter*, indicating severe microbiota dysbiosis induced by the treatment. With increasing AKK concentration, beneficial genera were significantly upregulated (*p*adj < 0.05), while pathogenic genera were markedly suppressed (*p*adj < 0.05), suggesting that AKK treatment effectively alleviates antibiotic- and LPS-induced microbial dysbiosis through a bidirectional regulatory mechanism, thereby promoting the restoration of a healthy microbial community structure. Collectively, these results demonstrate that AKK intervention robustly reverses ABX/LPS-induced gut microbiota dysbiosis and facilitates ecological recovery toward a healthier compositional state.

### 3.10 Predictive functional analysis of gut microbiota by PICRUSt2

To elucidate the overall impact of different treatments on the functional potential of the gut microbiota, this study employed PICRUSt2 to analyze and visualize KEGG pathway profiles (Figure 9F). PICRUSt2 is a phylogeny-based functional prediction method that can reveal potential functional shifts in microbial communities. Although it does not rely on direct metagenomic sequencing, this study employed PICRUSt2 to generate exploratory hypotheses regarding microbial functions, which were subsequently correlated with host phenotypic data. The prediction results demonstrated significant enrichment of ABC transporters and the phosphotransferase system (PTS) pathways in the NS + ABX/LPS group (*p* < 0.05), indicating that the residual microbiota under antibiotic pressure may enhance substrate transport capabilities to sustain viability. Furthermore, the coordinated activation of the MAPK signaling pathway and bacterial secretion systems suggests that opportunistic pathogens could increase their adaptability and pathogenic potential by modulating virulence factors and stress response mechanisms. As the concentration of AKK intervention increased, the enrichment levels of these pathways showed a progressive decrease. High-concentration AKK treatment significantly suppressed the aberrant activation of these pathways (*p* < 0.05), thereby impairing the adaptive response mechanisms of pathogenic bacteria. These functional predictions align well with the previously described phenotypic and community structure data, suggesting that AKK may indirectly inhibit the activation of inflammation-related signaling pathways such as MAPK by modulating microbiota-host interactions, ultimately contributing to the alleviation of intestinal inflammation. This interpretation is strongly supported by our prior experimental findings.

## 4 Discussion

Intestinal barrier integrity is fundamental to systemic health, yet its vulnerability is exacerbated by the widespread misuse of antibiotics, posing a substantial long-term public health challenge (Zhang et al., 2018; Li et al., 2024). The underlying mechanism of antibiotic-induced barrier disruption was systematically elucidated by Shai Bel and colleagues (Sawaed et al., 2024), who demonstrated that commonly used antibiotics—ampicillin, metronidazole, neomycin, and vancomycin—compromise the mucus layer, enabling bacterial translocation into the colonic epithelium and triggering a pathological cascade that leads to mucosal injury and disease onset. These findings were further supported by a nationwide Danish cohort study, which revealed a clear dose- and time-dependent association between antibiotic exposure and increased risk of intestinal disorders (Gorman et al., 2025). Oxidative stress plays a pivotal role in this process, creating a self-perpetuating feedback loop with impaired barrier function (Sui et al., 2024; Liu et al., 2020). Although accumulating evidence indicates that AKK may help maintain gut homeostasis through its antioxidant properties (Xia et al., 2022; Shuoker et al., 2023; Huo et al., 2022), its protective effects against antibiotic-mediated barrier damage and the specific molecular mechanisms involved remain poorly understood. This study aims to address this knowledge gap by comprehensively investigating the protective effects of AKK and characterizing its downstream signaling pathways in an experimental model of intestinal injury induced by antibiotics and LPS.

The intestine is safeguarded by a highly organized mucosal barrier that prevents uncontrolled microbial and antigen translocation while enabling regulated communication between luminal contents and the host immune system. The integrity of this barrier is primarily maintained by tight junction proteins (e.g., ZO-1, claudin, occludin) and mucins (e.g., MUC2), which jointly regulate intestinal permeability and serve as a critical defense against pathogen invasion (Landy et al., 2016). Evidence indicates that antibiotic treatment disrupts the gut microbiota, suppresses mucin secretion from goblet cells (Sawaed et al., 2024), and downregulates the expression of tight junction proteins, leading to increased intestinal permeability (Lin et al., 2025). These disruptions collectively contribute to the development and progression of intestinal inflammation. In contrast, AKK has been widely recognized for its unique capacity to modulate the mucus layer and enhance epithelial barrier repair (Luo et al., 2022). Our findings demonstrate that AKK supplementation significantly alleviates intestinal inflammation and restores barrier function in ABX/LPS-challenged mice. At the macroscopic level, AKK intervention reversed weight loss, improved the disease activity index, and ameliorated systemic symptoms in a dose-dependent manner, with more pronounced protective effects observed at higher doses. Mechanistically, AKK effectively repaired the antibiotic-disrupted mucus barrier, as evidenced by a marked restoration of goblet cell-derived MUC2 mucin expression. Furthermore, AKK robustly upregulated key tight junction proteins—including ZO-1, occludin, and claudin—thereby reinforcing the epithelial physical barrier. These results, consistently validated across immunohistochemistry, Western blot, and qPCR analyses, systematically elucidate the multi-faceted protective effects of AKK during intestinal inflammation. Specifically, AKK enhances overall intestinal defense by coordinately restoring both the mucus layer and tight junction barriers, thereby extending and corroborating previous findings (Camilleri, 2019; Ulluwishewa et al., 2011).

Oxidative stress is a central pathological mechanism underlying the development of intestinal diseases, exerting widespread effects on both the intestinal mucosa and deeper tissue layers (Sui et al., 2024; Liu et al., 2020). It activates the key transcription factor NRF2, triggering its translocation into the nucleus and initiating the expression of downstream antioxidant genes such as HO-1 and NQO1, thereby enhancing cellular defense against oxidative damage (Wang et al., 2023). Accumulating evidence has established a strong association between oxidative stress and intestinal barrier dysfunction, with tight junction proteins being particularly vulnerable to oxidative injury (Yu et al., 2022; Hao et al., 2021; Fishbein et al., 2023). Our findings reveal that AKK not only directly scavenges ROS but also, more significantly, promotes Nrf2 nuclear translocation, leading to the upregulation of HO-1 and NQO1. This coordinated response effectively activates the endogenous antioxidant defense system, as demonstrated by markedly increased SOD activity and GSH levels, along with reduced MDA concentrations. These results indicate that AKK alleviates oxidative damage caused by combined antibiotic and LPS challenge through multi-target regulation of redox homeostasis. Integrating our observations with existing literature, we propose that this antioxidant activity likely contributes to the preservation of tight junction integrity, thereby supporting the restoration of intestinal barrier function. This insight offers a novel theoretical framework for understanding the probiotic mechanisms of AKK and provides experimental justification for its potential therapeutic application in oxidative stress-related intestinal disorders.

The p38α MAPK signaling pathway plays a pivotal role in regulating cellular stress responses and serves as a key convergence point linking intestinal inflammation and oxidative stress (Meynier et al., 2024; Zhao et al., 2023; Bourgonje et al., 2020). Its involvement in various gut pathological conditions is well established. For example, carbon monoxide attenuates intestinal inflammation by inhibiting p38α MAPK activation (Xu et al., 2025), while lactoferrin protects against heat stress-induced intestinal barrier dysfunction through suppression of the p38α MAPK signaling pathway (Gao et al., 2025). Although the role of the p38 MAPK family in intestinal disease is well recognized, the clinical development of pan-p38 inhibitors has repeatedly faltered due to a lack of isoform selectivity, underscoring the need for more precise mechanistic targeting. Individual p38 MAPK isoforms often exert distinct—and at times opposing—biological functions. Among them, p38α MAPK not only represents the most extensively characterized isoform but is also regarded as a highly promising therapeutic target due to its central role in inflammatory and stress responses. Therefore, this study focuses specifically on p38α MAPK—the most druggable isoform—to investigate whether AKK exerts its barrier-protective effects via selective resetting of the p38α MAPK–Nrf2 kinase module, rather than through broad suppression of the entire pathway.

Since its initial isolation at Wageningen University in 2004 (Qin et al., 2024), AKK has been increasingly associated with a reduced risk of oxidative stress-related disorders (Du et al., 2025; Li et al., 2023). Clinical studies show that AKK levels are markedly reduced in metabolic diseases such as obesity and non-alcoholic fatty liver disease, where its depletion correlates inversely with markers of oxidative damage (Xu et al., 2020). In neurological contexts, decreased AKK abundance is closely linked to the accumulation of neurotoxic substances driven by oxidative stress (Ma et al., 2023). Despite these robust associations, the precise role and underlying mechanisms by which AKK modulates antibiotic-associated mucosal oxidative stress remain unclear.

Based on a systematic experimental design, this study reveals for the first time that the gut-protective mechanism is mediated by specific modulation of the p38*α* MAPK–Nrf2 signaling module, rather than through broad inhibition of p38 MAPK. Pharmacologically, a p38α-specific inhibitor successfully recapitulated the core protective effects of AKK. Genetically, siRNA-mediated silencing of MAPK14 abolished LPS-induced hyperphosphorylation of p38α and concurrently abrogated the barrier-protective effect of AKK, thereby identifying p38α as the key isoform responsible for mediating AKK function. Mechanistically, AKK induces dephosphorylation of p38α MAPK, which alleviates its post-translational suppression of Nrf2 and subsequently activates a downstream antioxidant gene network. This mechanism is consistent with previous reports indicating that p38 MAPK can promote Nrf2 protein degradation or inhibit its transcriptional activity (Zhuge et al., 2025; Hegde et al., 2024; Krawczyk et al., 2024; Zhou et al., 2021). Our findings not only define a complete AKK–p38α MAPK–Nrf2 signaling axis but also establish a novel theoretical framework for understanding how gut microbiota regulate host antioxidant defenses, offering an experimental basis for developing microecological therapeutics to prevent antibiotic-associated intestinal injury.

In recent years, AKK has exhibited a range of probiotic properties that surpass those of conventional probiotics. Not only does it degrade mucin to produce SCFAs, conferring various health benefits (Huang et al., 2021), but it also promotes the growth of other commensal bacteria, thereby helping to preserve gut microbial diversity (Derrien et al., 2004; Depommier et al., 2019; Rodrigues et al., 2022). Leveraging this distinctive ecological function, we further explored AKK’s capacity to restore the gut microbiota in a mouse model following antibiotic-induced disruption. 16S rRNA gene sequencing demonstrated that AKK administration significantly enhanced its own intestinal colonization and triggered a comprehensive reconfiguration of the microbial community, leading to marked improvements in both α- and *β*-diversity. Notably, AKK facilitated the recovery of key SCFA-producing genera, including *Bacteroides, Bifidobacterium*, and *Blautia*, while concurrently inhibiting the overgrowth of potential pathogens such as *Escherichia, Enterobacter, Paraeggerthella,* and *Erysipelatoclostridium.* Notably, LEfSe analysis and PICRUSt2-based functional prediction revealed that these microbial alterations were significantly associated with the MAPK signaling pathway. Consistent with our earlier findings, in addition to directly modulating host signaling pathways, AKK significantly remodeled the gut microbiota disrupted by antibiotics and LPS. These structural and functional changes in the microbial community may occur through the restoration of beneficial symbionts that produce protective barrier metabolites and the suppression of pathogenic microbes capable of triggering microbiota-derived oxidative stress, thereby indirectly promoting the normalization of the p38α MAPK–Nrf2 axis and the repair of the intestinal barrier. Thus, microbial remodeling may serve as an important ecological context for the comprehensive protective effects of AKK (Li et al., 2023; Hirayama et al., 2023).

This study is the first to reveal a novel mechanism whereby AKK alleviates intestinal oxidative stress by modulating the p38α MAPK-Nrf2 signaling module. It should be noted, however, that our demonstration of Nrf2’s role relies primarily on observations of its activation pattern. Although we have shown a strong association between upstream p38α MAPK inhibition and downstream Nrf2 activation coupled with functional improvement—and this regulatory relationship is consistent with established mechanisms—direct experimental confirmation of Nrf2’s necessity in this pathway, such as through the use of Nrf2-specific inhibitors or genetic knockout approaches, will be required in future work. This represents a key direction for subsequent investigation.

## 5 Conclusion

**Figure 10:**
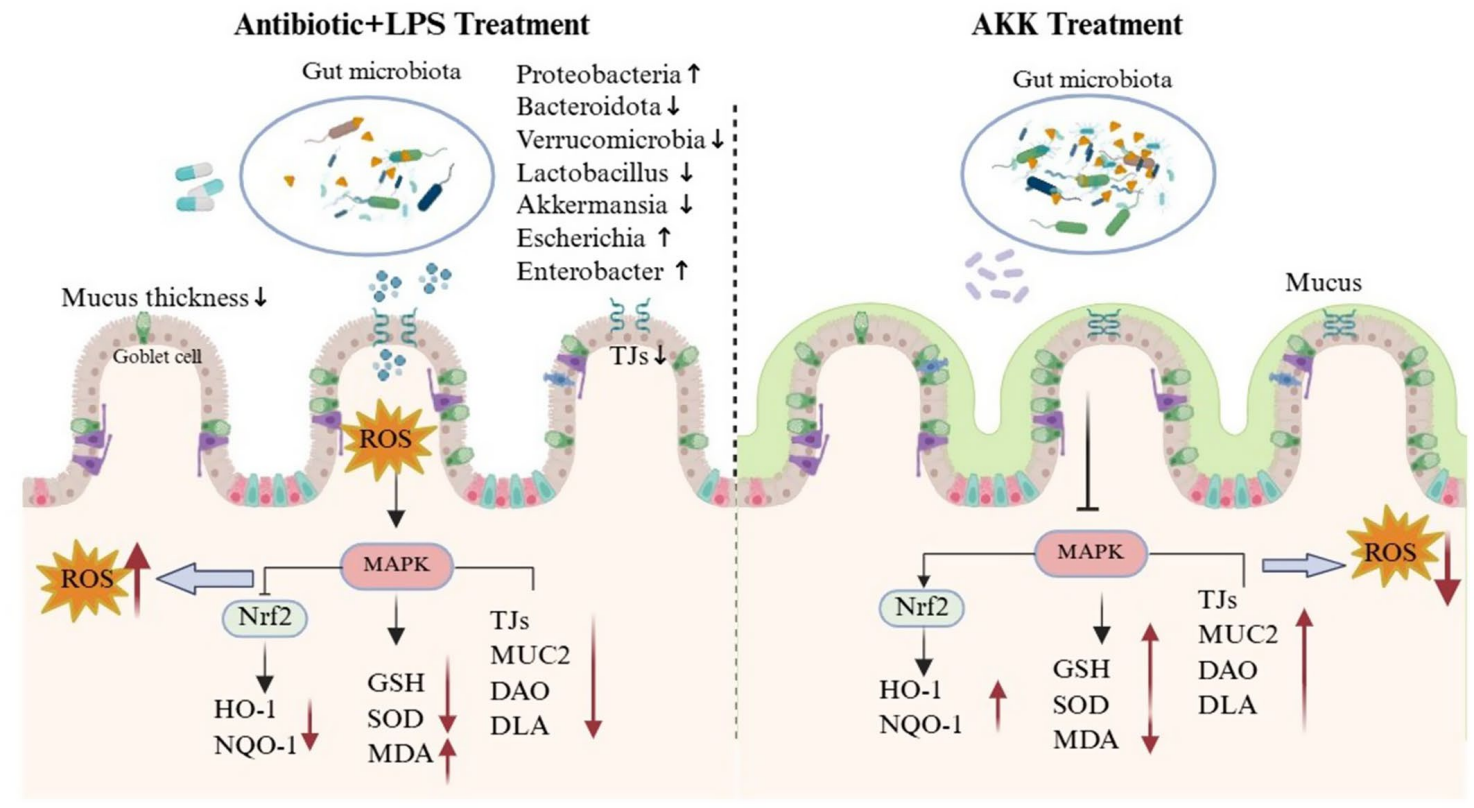
A schematic model illustrating the role of the AKK-p38α MAPK-Nrf2 axis and microbial regulation in restoring intestinal homeostasis.

Based on our findings, this study demonstrates for the first time that combined exposure to antibiotics and LPS induces intestinal barrier injury by triggering oxidative stress through hyperphosphorylation of p38α MAPK and subsequent disruption of the p38α MAPK–Nrf2 signaling module. In contrast, AKK exerts gut-protective effects via a “host-microbe dual regulation” mechanism (Figure 10). First, it directly targets the host by inhibiting p38α MAPK signaling, thereby activating the Nrf2-mediated antioxidant pathway. Second, it indirectly reshapes the gut microbial ecosystem. These two mechanisms act synergistically to alleviate oxidative stress and restore intestinal barrier integrity. Our results not only establish a theoretical basis for the clinical application of AKK but also enhance understanding of probiotic–host interaction networks, offering new perspectives for developing targeted microecological interventions against gut-related diseases.

## Data Availability

The 16S rRNA data presented in the study are deposited in the NGDC BioProject repository (https://ngdc.cncb.ac.cn/gsa), accession number CRA037978.

## Footnotes

1
http://hdock.phys.hust.edu.cn/
