# Supplementary material for: Akkermansia muciniphila alleviates antibiotic- and LPS-induced oxidative stress via the p38α MAPK–Nrf2 signaling axis
Source: Front Microbiol. 2026 Feb 10;17:1753421. doi: 10.3389/fmicb.2026.1753421 (PMC12931279; doi:10.3389/fmicb.2026.1753421)
Supplement: Supplementary file 1 [file Supplementary_file_1.docx]

Supplementary Material

# Supplementary Tables

**Supplementary Table 1.** List of quantitative RT-PCR primers.

| Gene | Primer | Sequence (5′to 3′) |
| --- | --- | --- |
| GAPDH（Mouse） | Forward primer | AGAAGGTGGTGAAGCAGGCATC |
|  | Reverse primer | CGAAGGTGGAAGAGTGGGAGTTG |
| GAPDH（Homo） | Forward primer | GGAGTCCACTGGCGTCTTCA |
|  | Reverse primer | GTCATGATCCTTCCACGATACC |
| ZO-1（Mouse） | Forward primer | ACCCGAAACTGATGCTGTGGATAG |
|  | Reverse primer | GCTGGCTGGCTGTACTGTGAG |
| ZO-1（Homo） | Forward primer | GTGGGTAACGCCATCCTCTG |
|  | Reverse primer | AGGGTTTTCCTTGGCTGACA |
| Occludin（Mouse） | Forward primer | CACACCTCGTCGCTAGTGC |
|  | Reverse primer | CTCCCAAGATAAGCGAACCTGC |
| Occludin（Homo） | Forward primer | TTGCGGCGAGCGGATTG |
|  | Reverse primer | GCCTGGATGACATGGCTGAT |
| Claudin-1（Mouse） | Forward primer | GTGTCCTACTTTCCTGCTCCTGTC |
|  | Reverse primer | AGAAGGTGTTGGCTTGGGATAAGG |
| Claudin-1（Homo） | Forward primer | TCCTCCAGTTAGGAGCCTTGA |
|  | Reverse primer | CTCTCGGCGACCCACTGTC |
| Nrf2（Mouse） | Forward primer | CTACAGTCCCAGCAGATGATG |
|  | Reverse primer | CCAAAACTTGTACCGCCTCG |
| Nrf2（Homo） | Forward primer | AGCAAGTTTGGGAGGAGCTATTA |
|  | Reverse primer | GAGAGGATGCTGCTGAAGGAATC |
| HO-1（Mouse） | Forward primer | ACCTTCCCGAACATCGACAG |
|  | Reverse primer | CAGCTCCTCAAACAGCTCAATG |
| HO-1（Homo） | Forward primer | AGTTCAAGCAGCTCTACCGC |
|  | Reverse primer | GCAACTCCTCAAAGAGCTGGAT |
| NQO-1（Mouse） | Forward primer | CATTCGTCTCTGTCCGTCTGT |
|  | Reverse primer | GAAGTAACACAATGGGCTTGG |
| NQO-1（Homo） | Forward primer | CCCTGCAGTGGTTTGGAGT |
|  | Reverse primer | CACTGCCTTCTTACTCCGGAAGG |
| p38αMAPK（Mouse） | Forward primer | TCATGGCTGAGCTGTTGACC |
|  | Reverse primer | TGTAGTTTCTTGCCTCATGGC |
| p38αMAPK（Homo） | Forward primer | GTGTTGCAGATCCAGACCATGAT |
|  | Reverse primer | TGCAGCCTACAGACCAAATATCA |

**
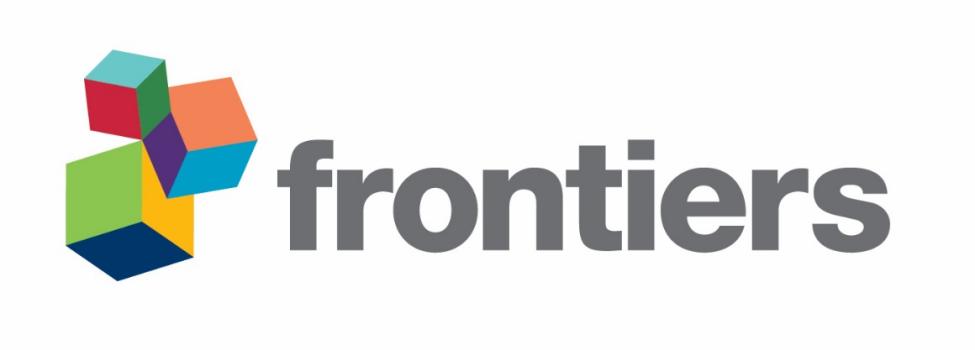
**
