# Supplementary material for: Akkermansia muciniphila alleviates antibiotic- and LPS-induced oxidative stress via the p38α MAPK–Nrf2 signaling axis
Source: Front Microbiol. 2026 Feb 10;17:1753421. doi: 10.3389/fmicb.2026.1753421 (PMC12931279; doi:10.3389/fmicb.2026.1753421)
Supplement: Supplementary file 2 [file Supplementary_file_2.docx]

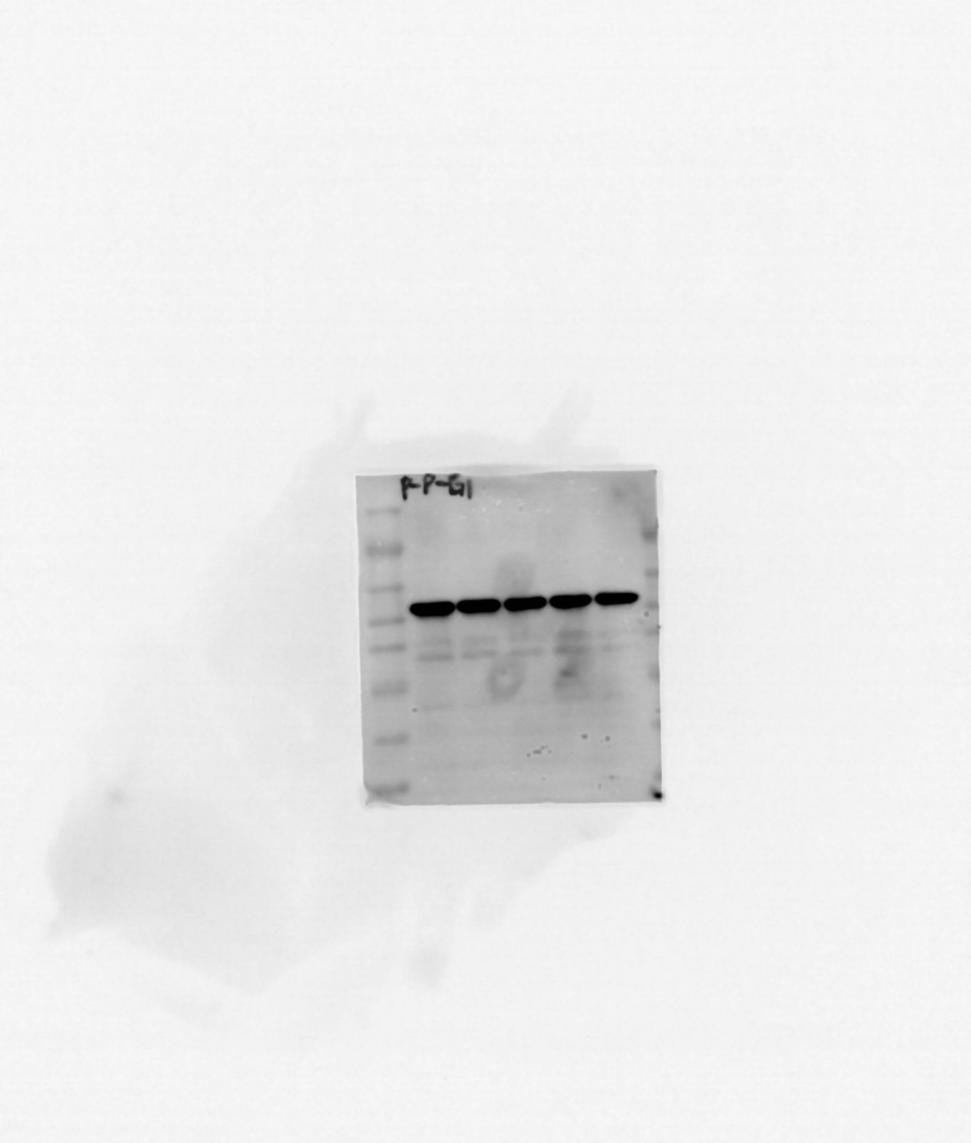

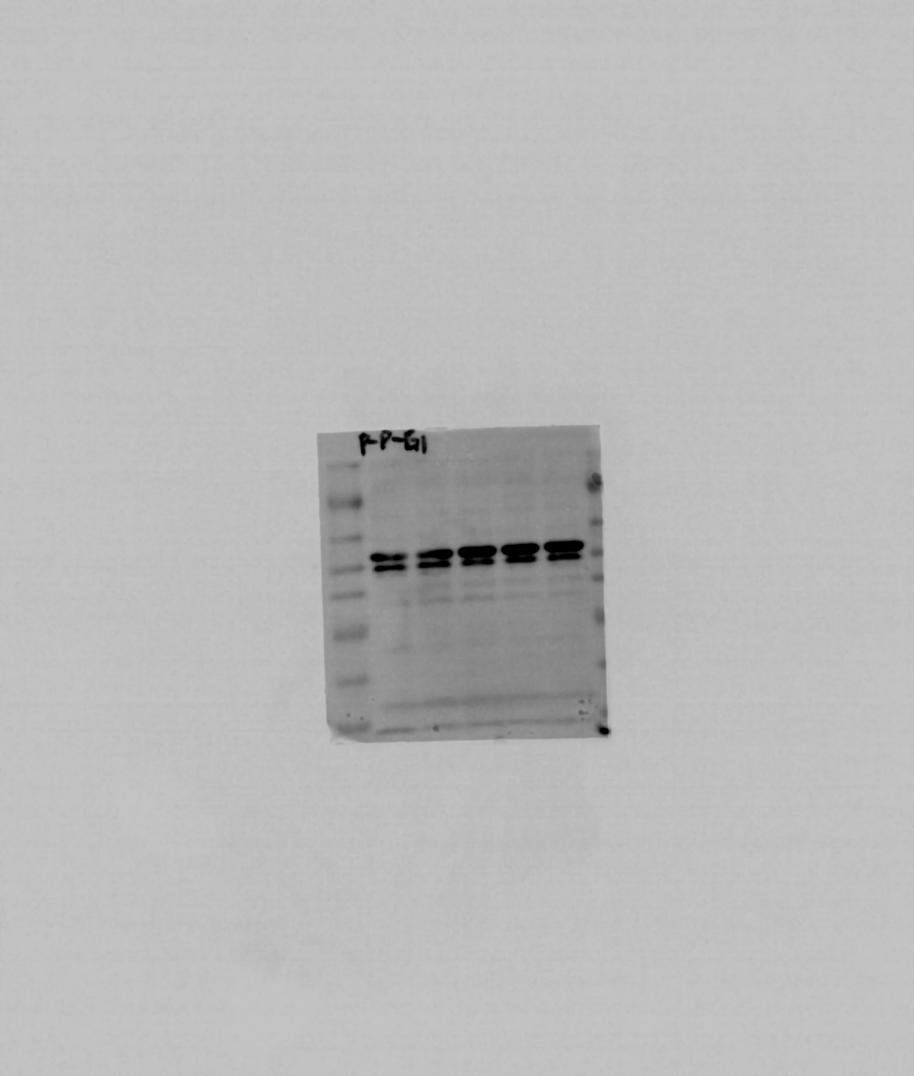

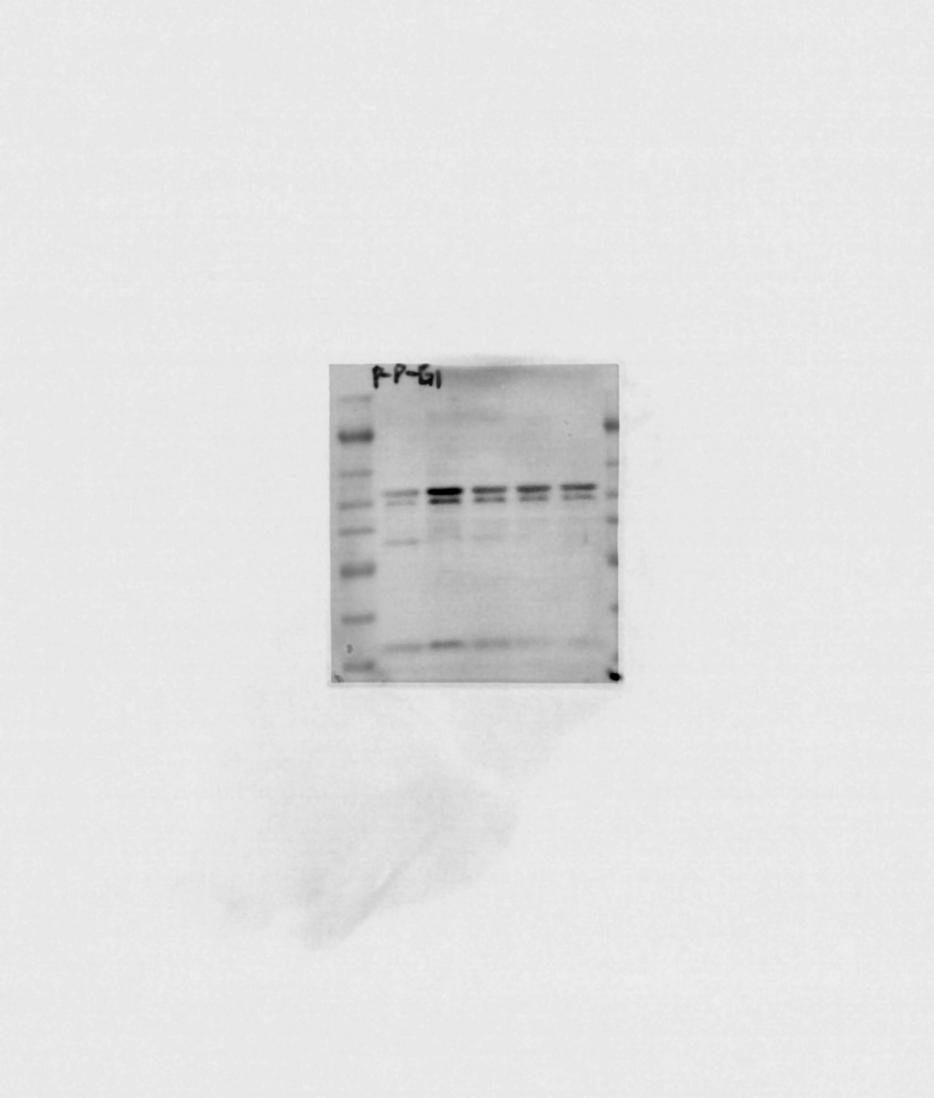


40KD

LPS

LPS

AKK+LPS

AKK+LPS

p38KD

LPS

P38KD+AKK+LPS

AKK+LPS

P38KD+LPS

p38KD

P38KD+AKK+LPS

P38KD+LPS

p38KD

P38KD+AKK+LPS

P38KD+LPS

p-p38αMAPK p38αMAPK β-actin

**Figure 1**


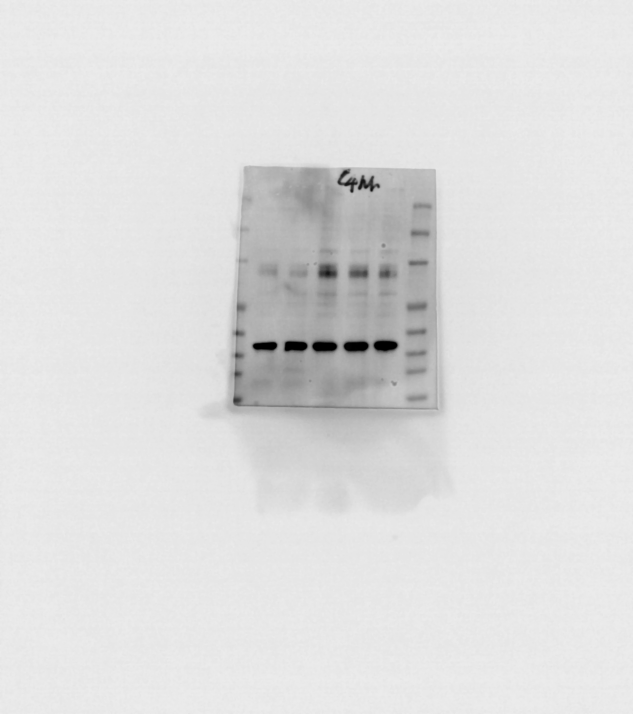


40KD


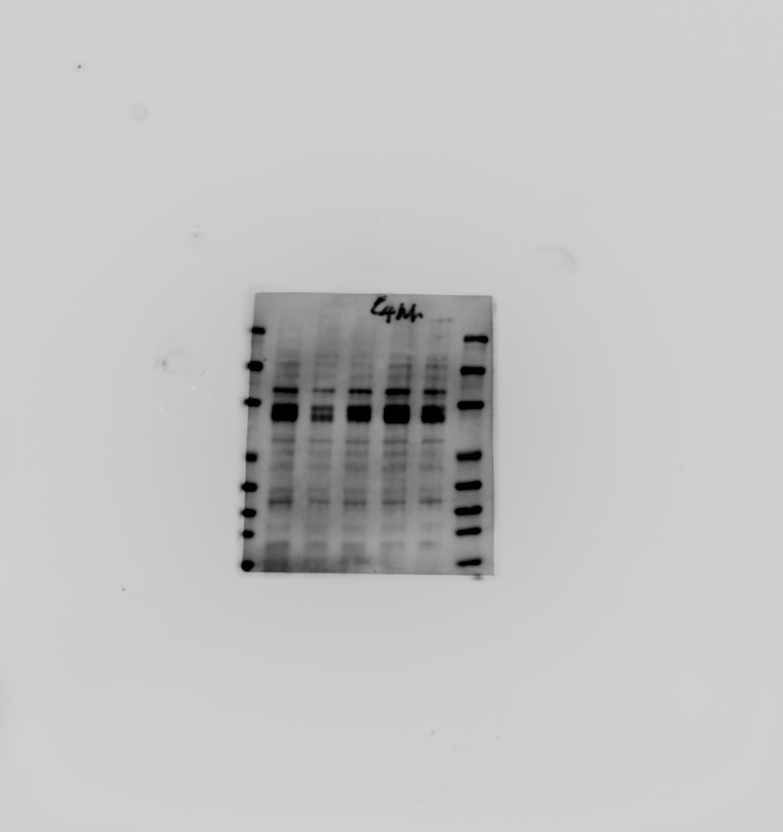


100KD

p38KD

LPS

P38KD+LPS

AKK+LPS

P38KD+AKK+LPS

P38KD+AKK+LPS

AKK+LPS

P38KD+LPS

LPS

p38KD

Nrf2 β-actin


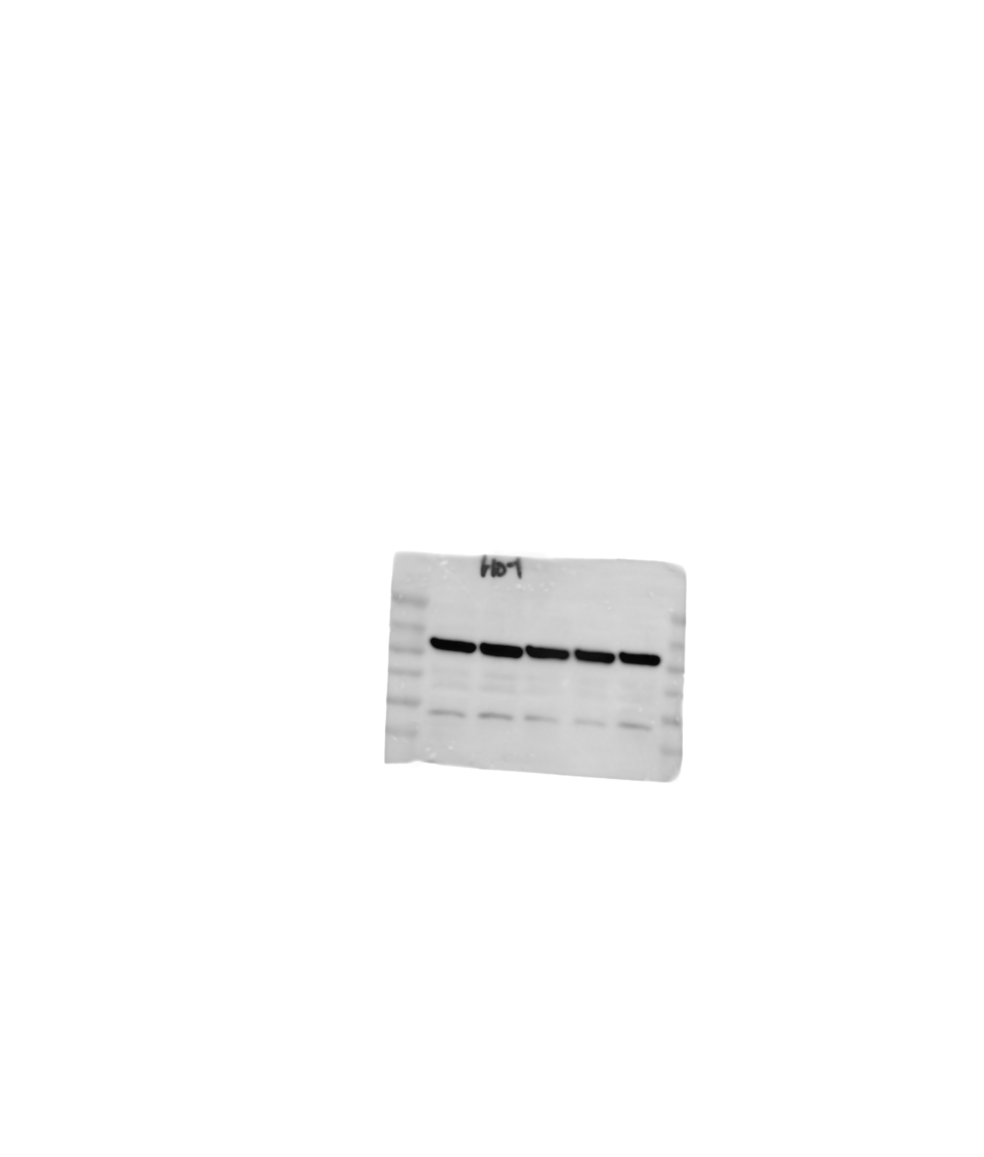


40KD


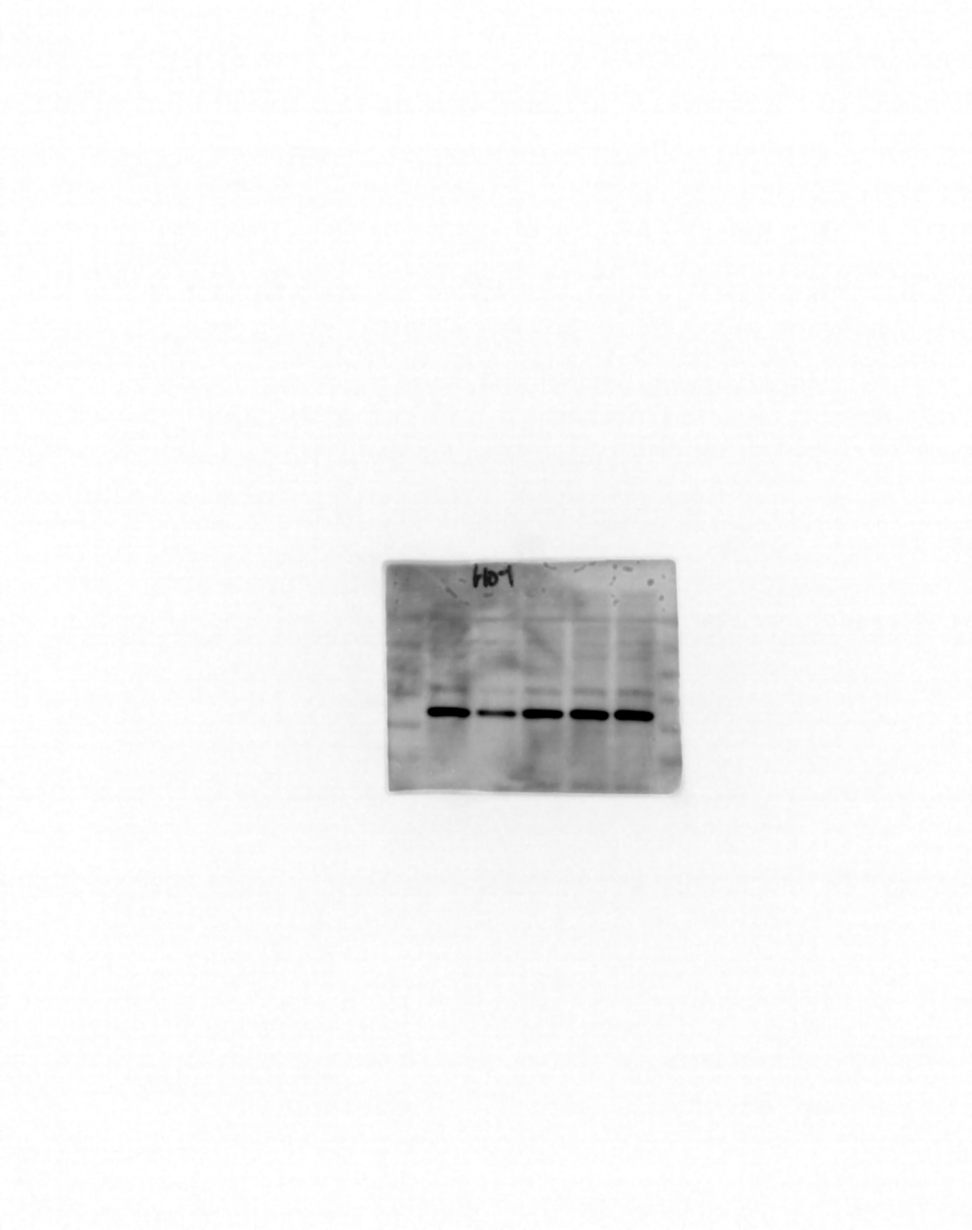


25KD

35KD

P38KD+AKK+LPS

AKK+LPS

P38KD+LPS

LPS

p38KD

AKK+LPS

P38KD+LPS

p38KD

LPS

P38KD+AKK+LPS

HO-1 β-actin


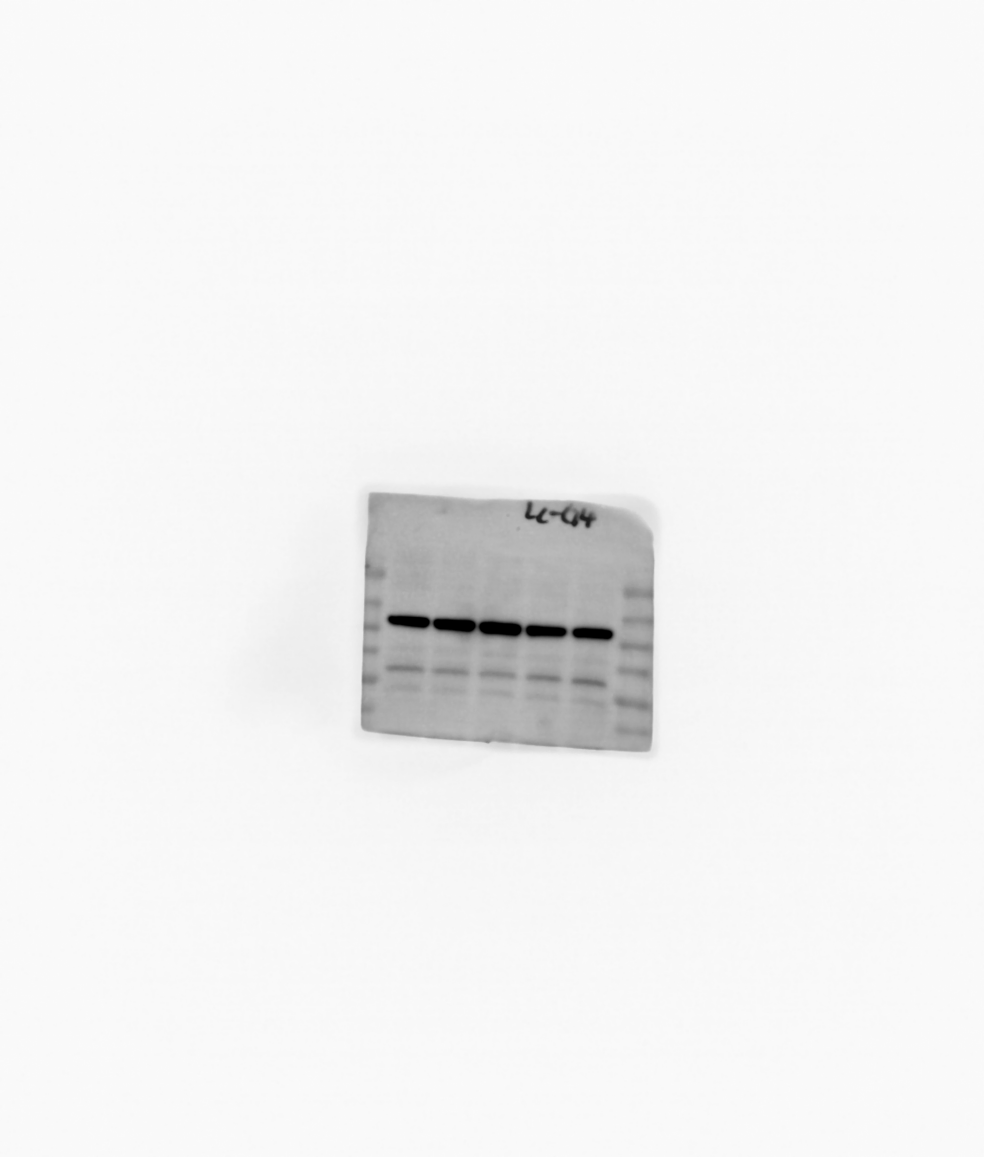


40KD


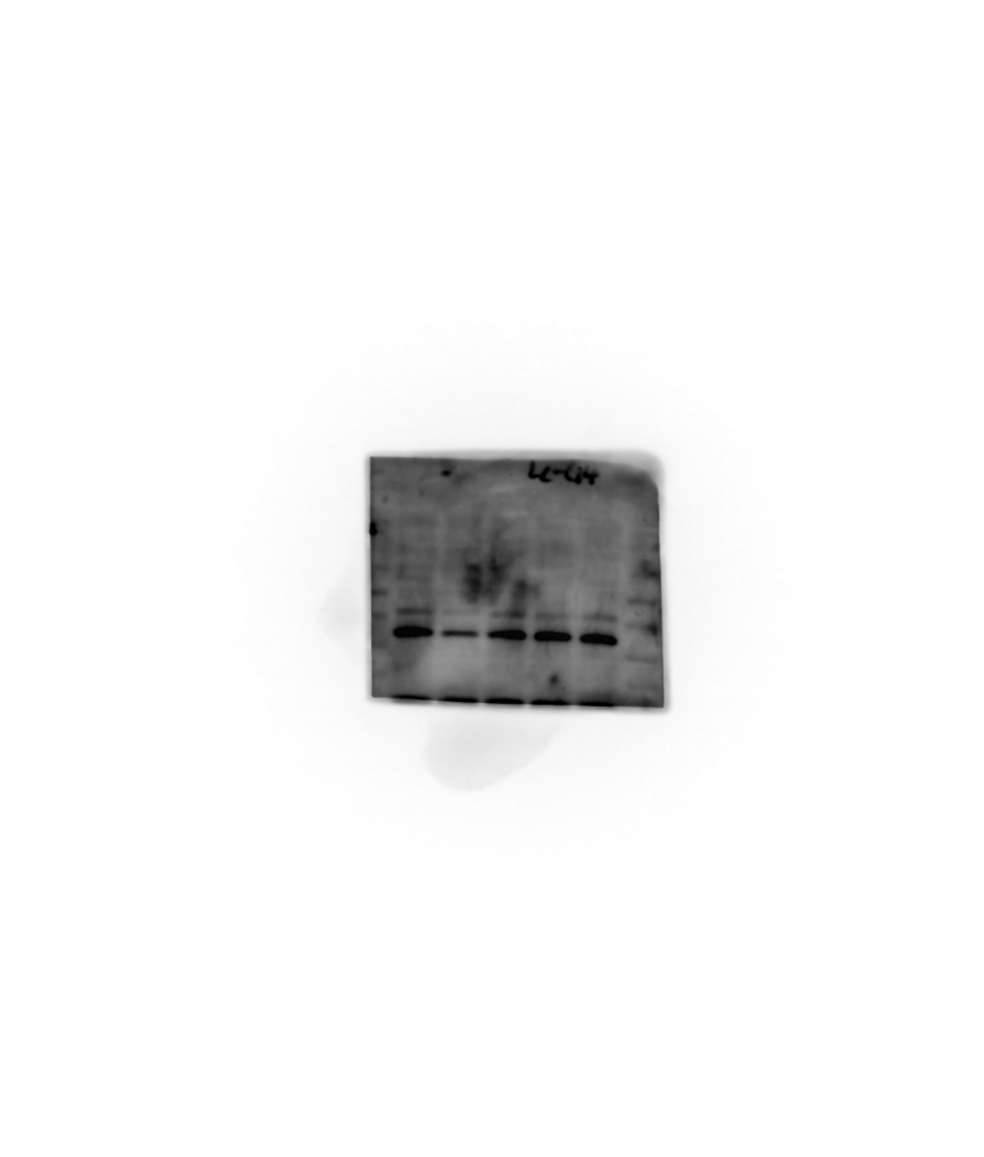


35KD

25KD

LPS

AKK+LPS

p38KD

LPS

AKK+LPS

P38KD+LPS

p38KD

P38KD+AKK+LPS

P38KD+LPS

P38KD+AKK+LPS

NQO-1 β-actin

**Figure 2**


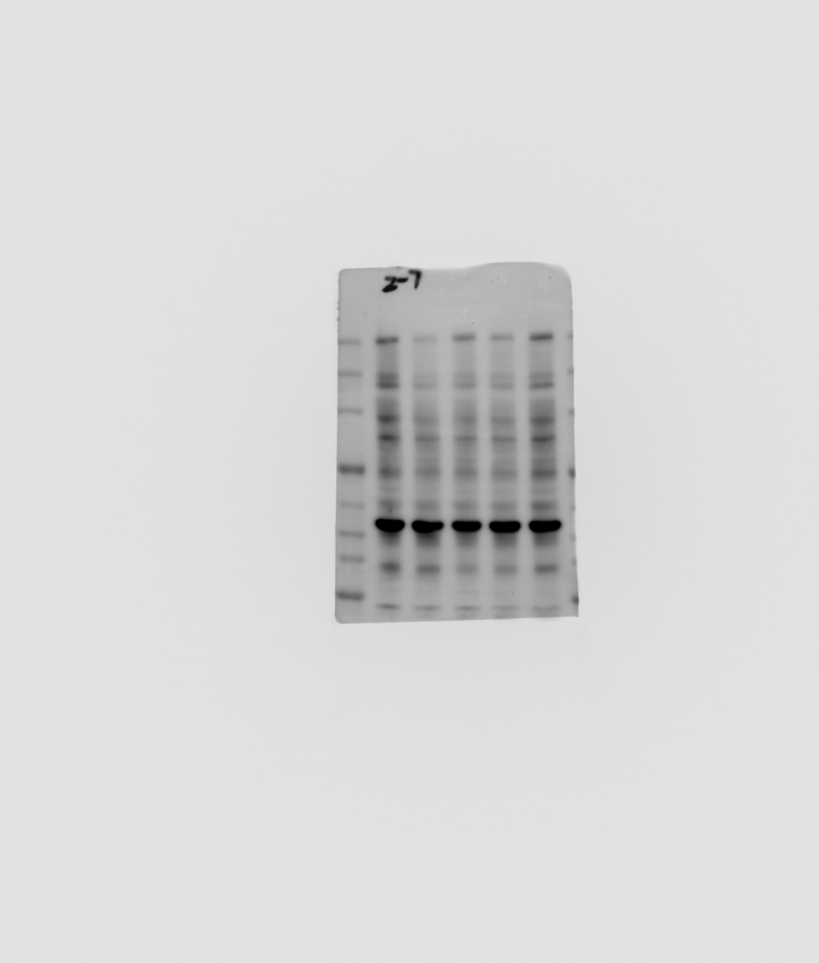


40KD


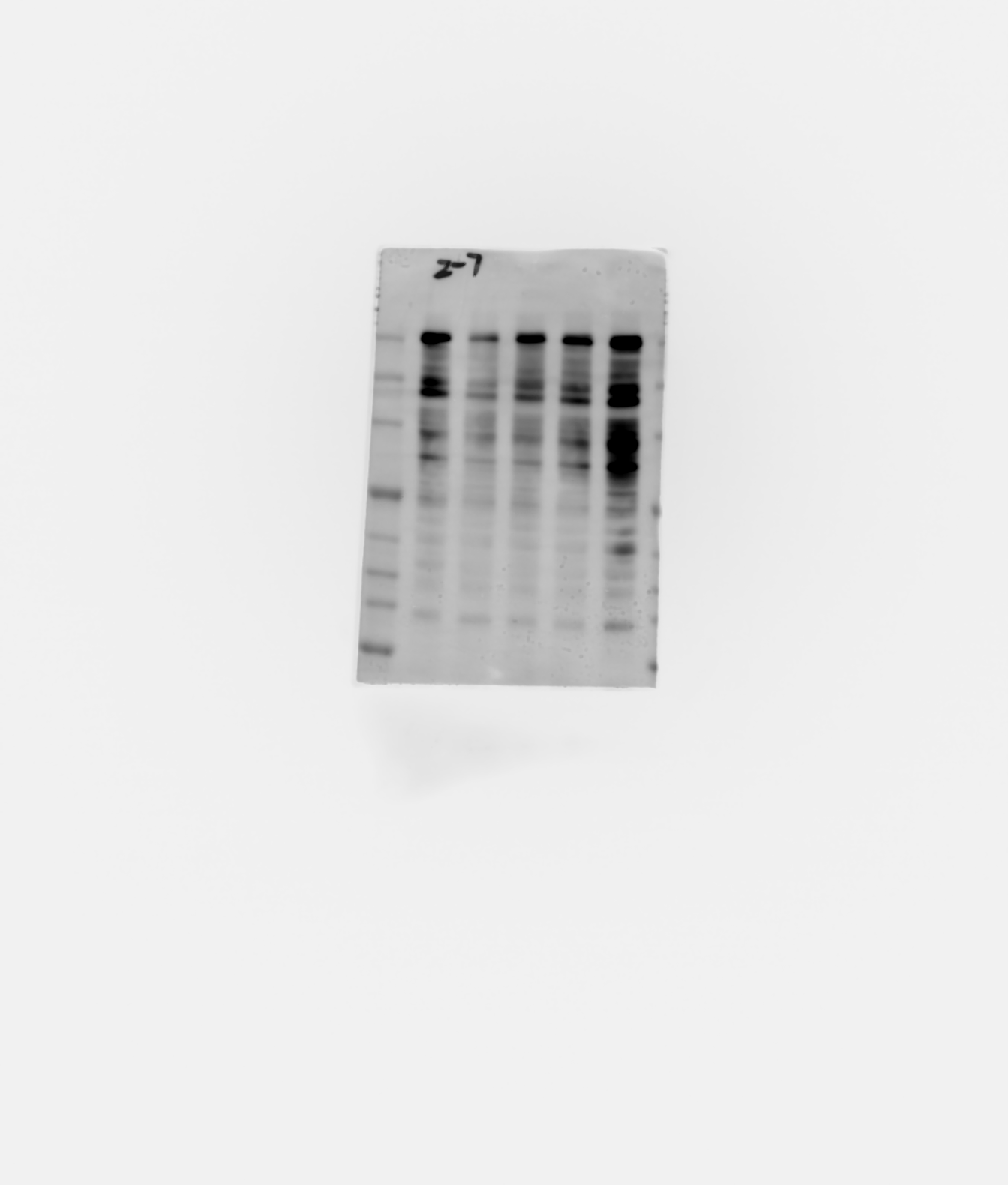


250KD

P38KD+LPS

AKK+LPS

p38KD

LPS

P38KD+AKK+LPS

LPS

P38KD+LPS

P38KD+AKK+LPS

AKK+LPS

p38KD

ZO-1 β-actin


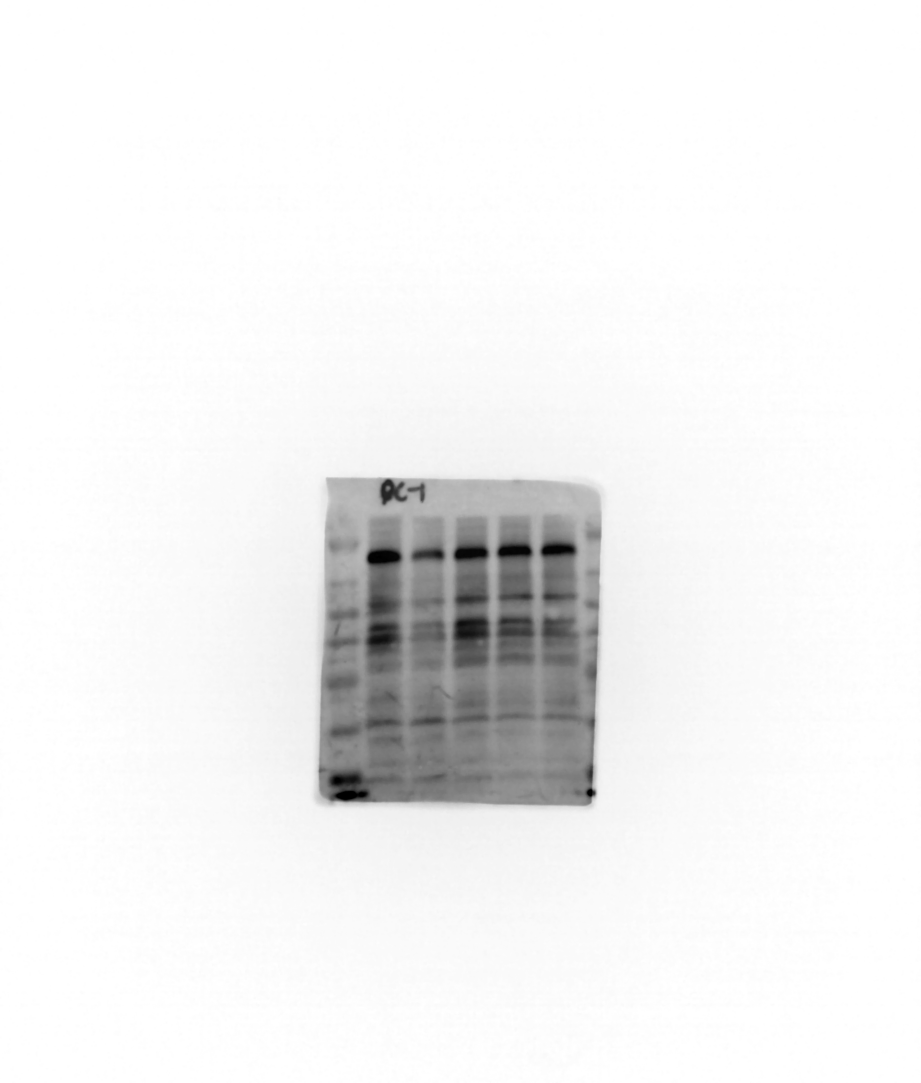


70KD


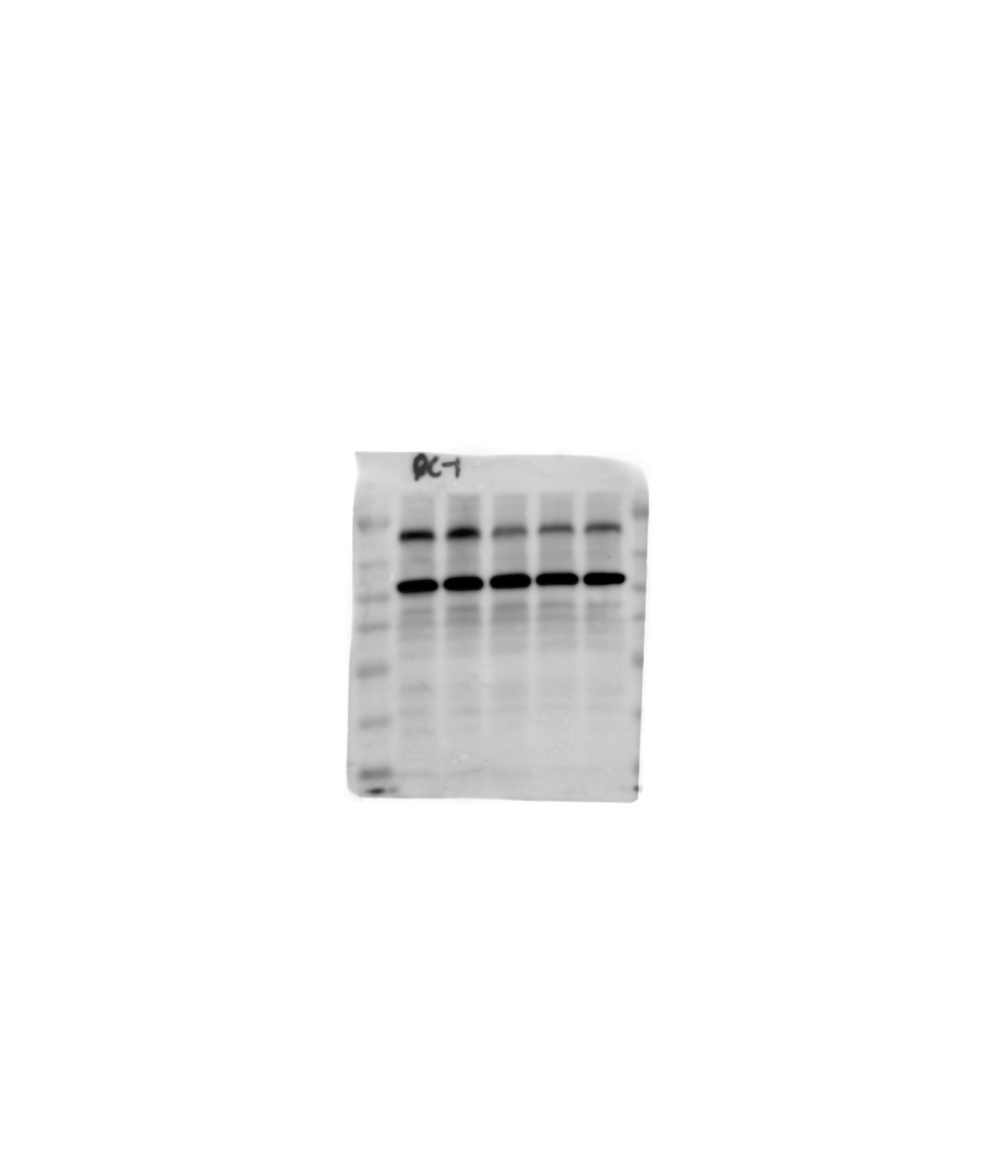


40KD

LPS

AKK+LPS

p38KD

P38KD+AKK+LPS

LPS

AKK+LPS

p38KD

P38KD+AKK+LPS

P38KD+LPS

P38KD+LPS

Occludin β-actin


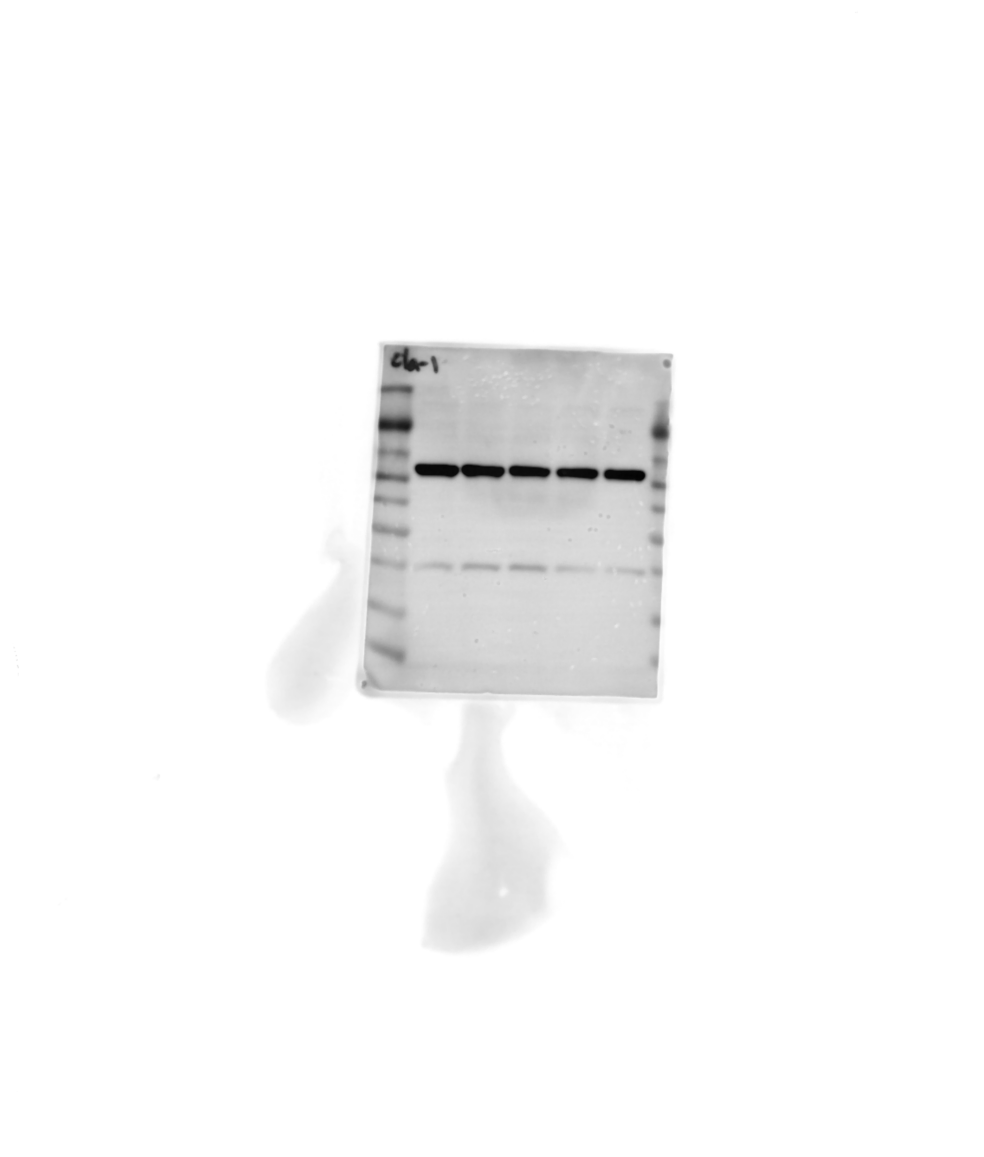


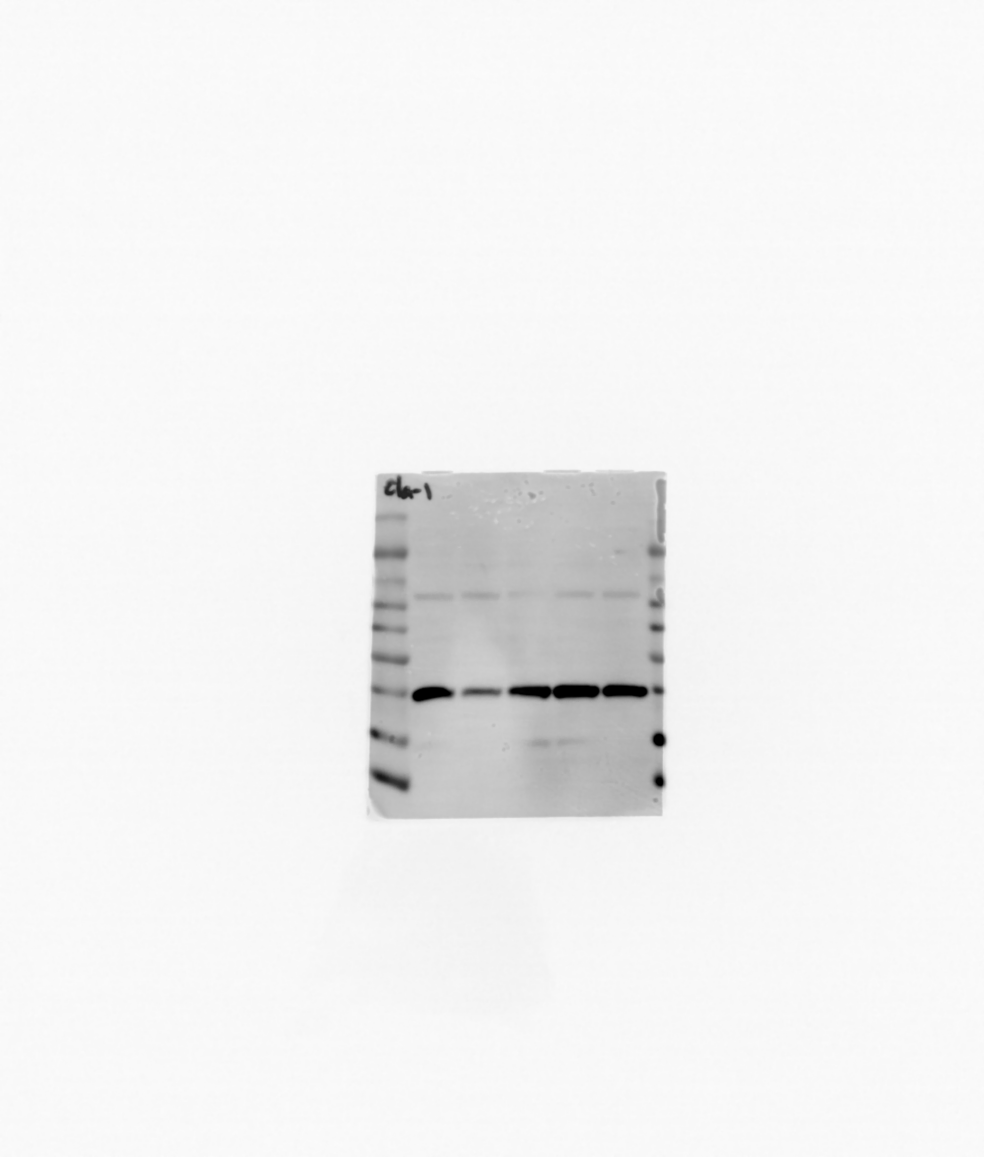


25KD

40KD

P38KD+LPS

AKK+LPS

p38KD

P38KD+AKK+LPS

LPS

P38KD+LPS

AKK+LPS

p38KD

P38KD+AKK+LPS

LPS

LPS

AKK+LPS

p38KD

P38KD+AKK+LPS

Claudin-1 β-actin

**
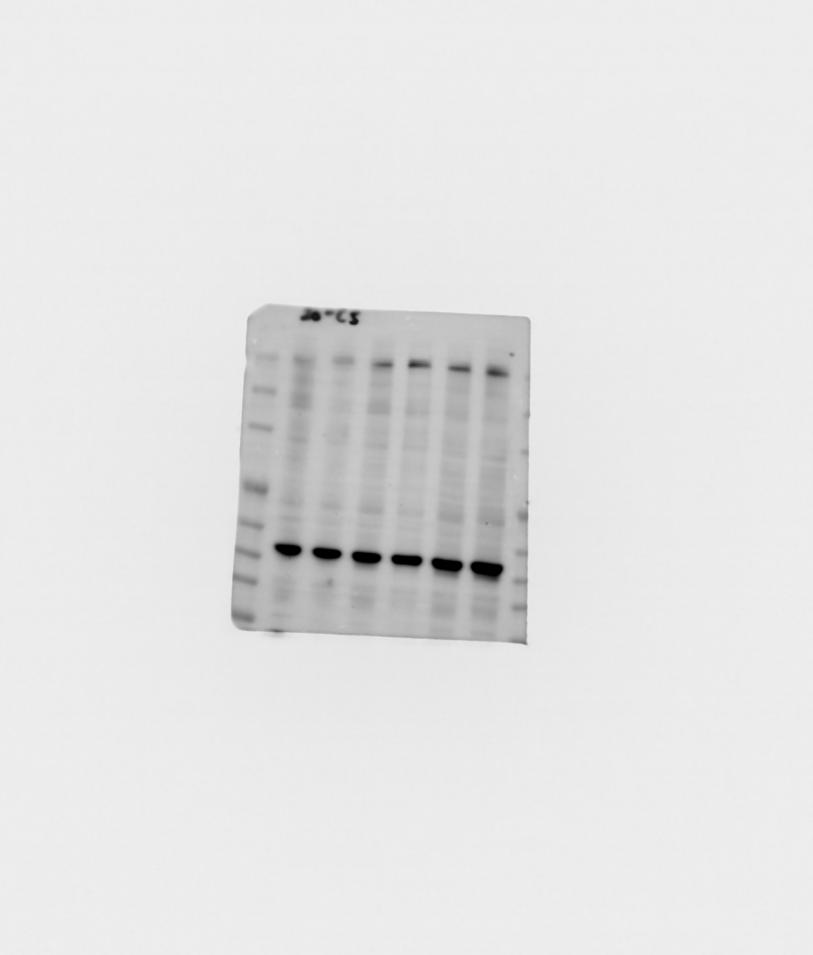

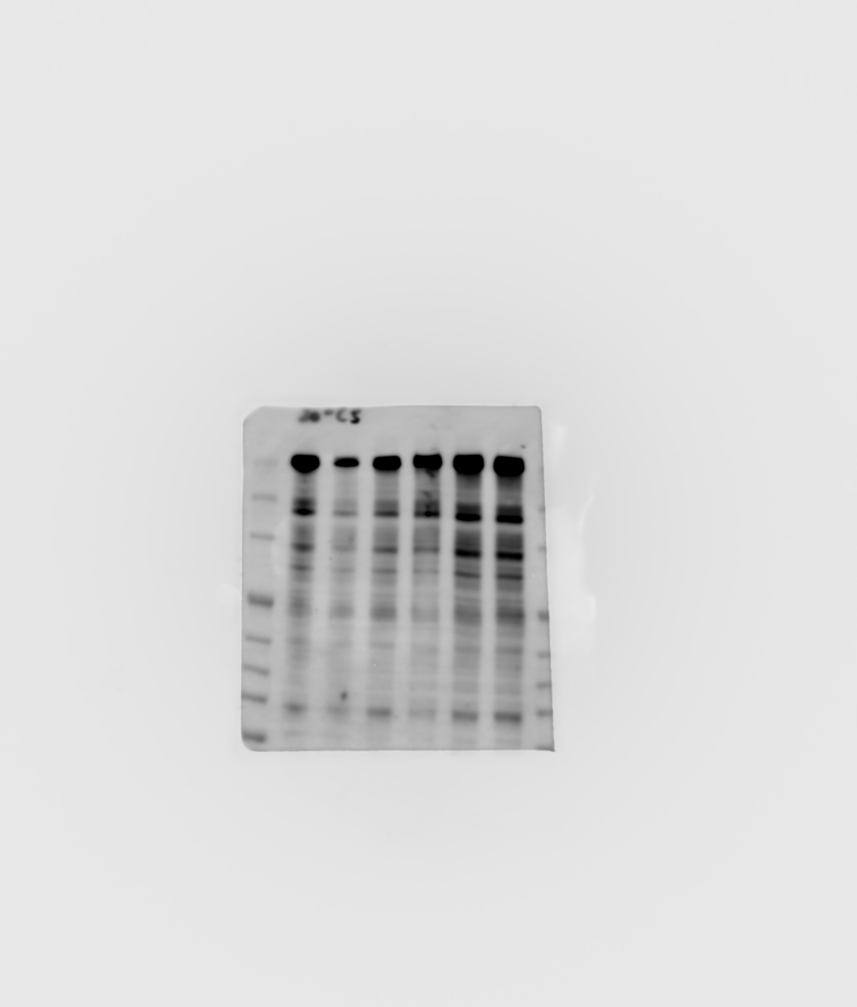
Figure 3**

250KD

40KD

NS+ABX/LPS

AKK(L)+ABX/LPS

AKK(M)+ABX/LPS

AKK(H)+ABX/LPS

SB203580+ABX/LPS

NC

NC

AKK(L)+ABX/LPS

NS+ABX/LPS

AKK(M)+ABX/LPS

AKK(H)+ABX/LPS

SB203580+ABX/LPS

ZO-1 β-actin


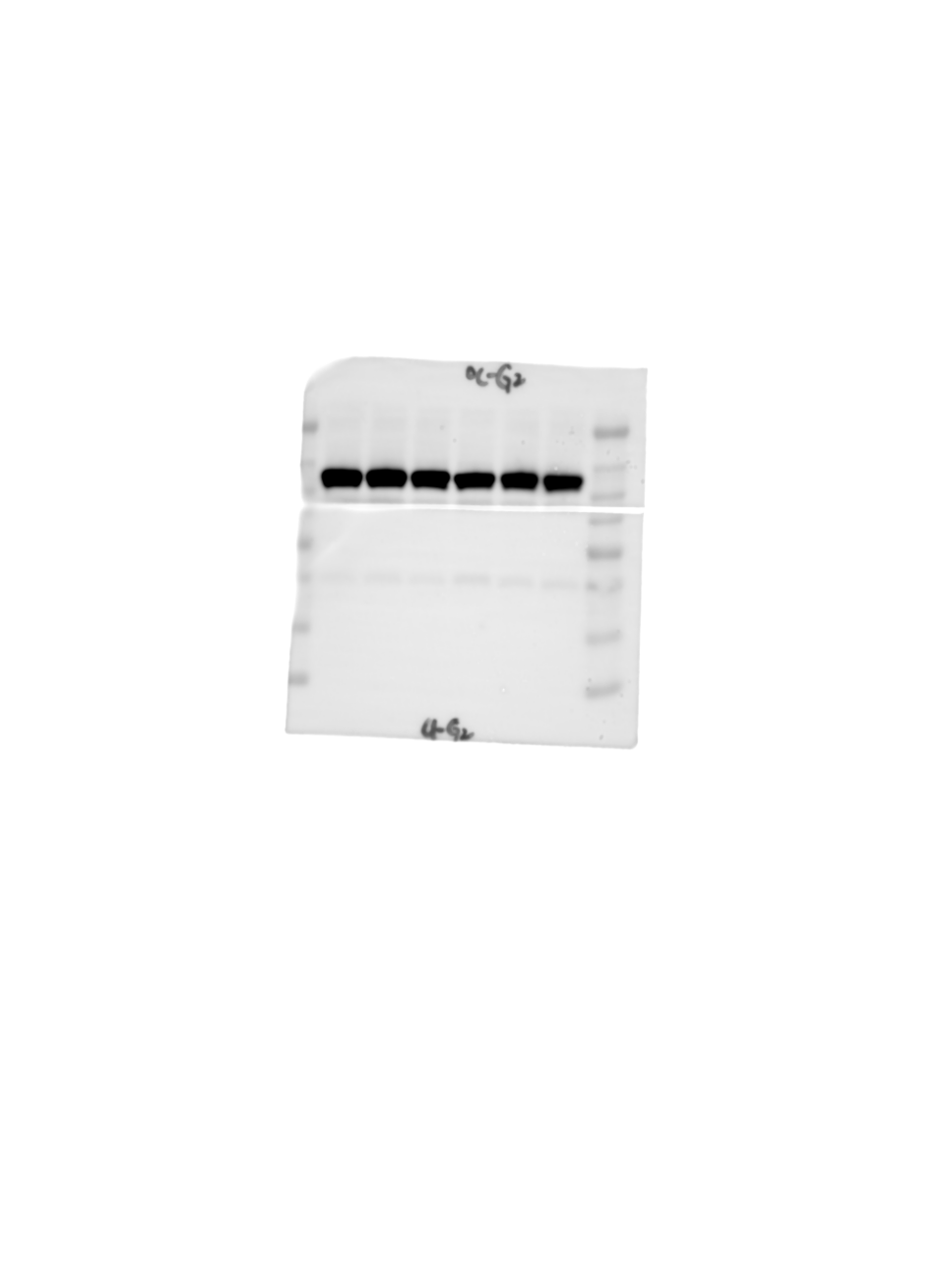


40KD


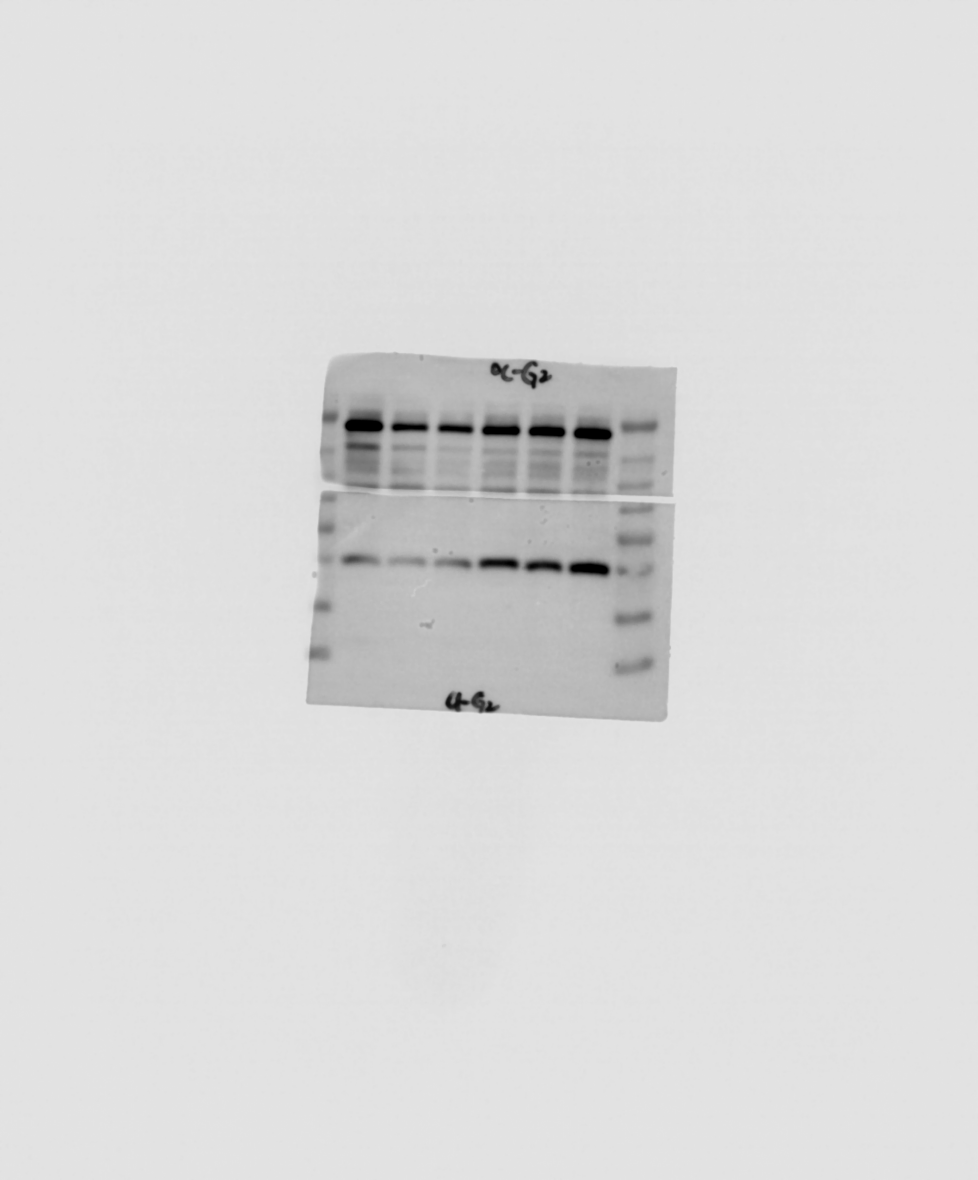


70KD

NS+ABX/LPS

AKK(L)+ABX/LPS

AKK(M)+ABX/LPS

AKK(H)+ABX/LPS

SB203580+ABX/LPS

NC

NS+ABX/LPS

AKK(L)+ABX/LPS

AKK(M)+ABX/LPS

AKK(H)+ABX/LPS

SB203580+ABX/LPS

NC

Occludin β-actin


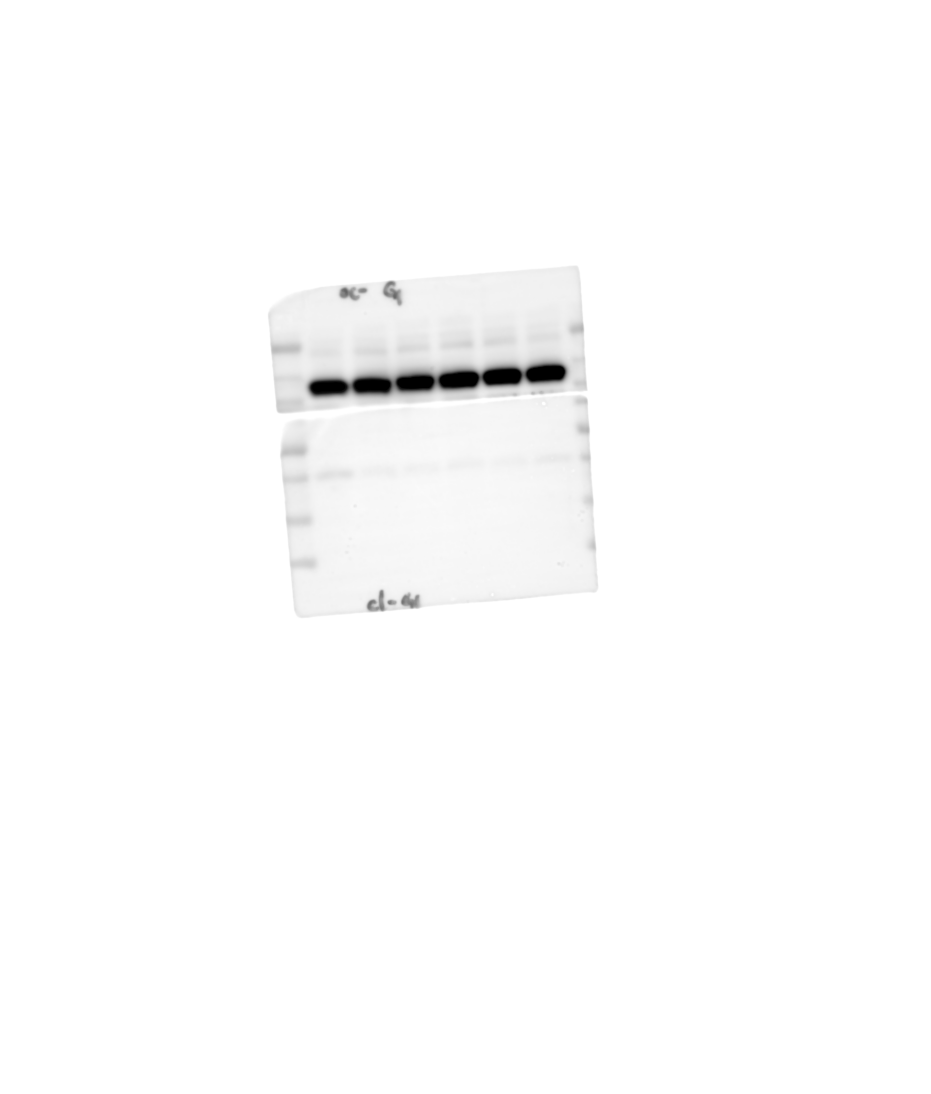


40KD


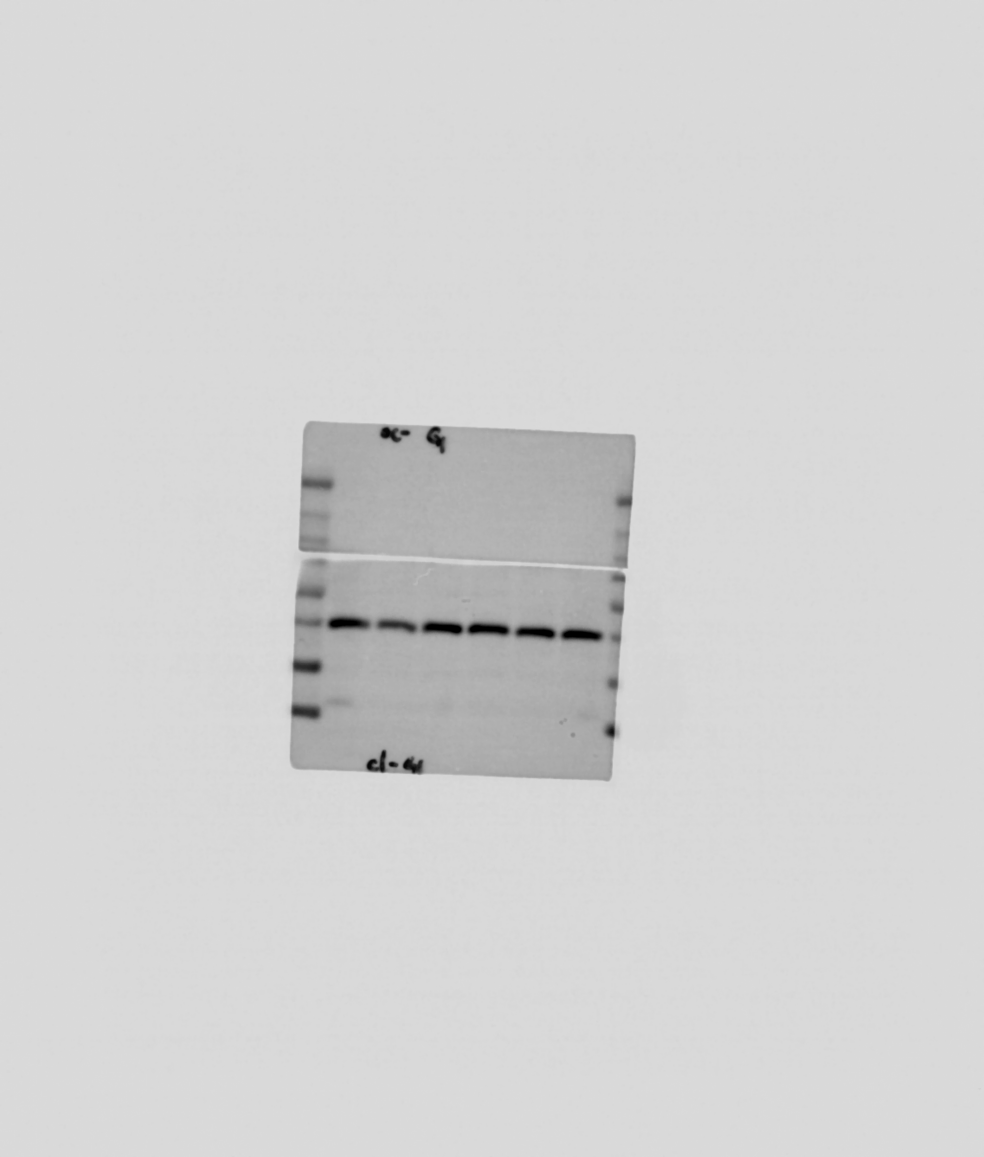


25KD

NS+ABX/LPS

AKK(L)+ABX/LPS

AKK(M)+ABX/LPS

AKK(H)+ABX/LPS

SB203580+ABX/LPS

NC

NS+ABX/LPS

AKK(L)+ABX/LPS

AKK(M)+ABX/LPS

AKK(H)+ABX/LPS

SB203580+ABX/LPS

NC

Claudin-1 β-actin

**Figure 5**


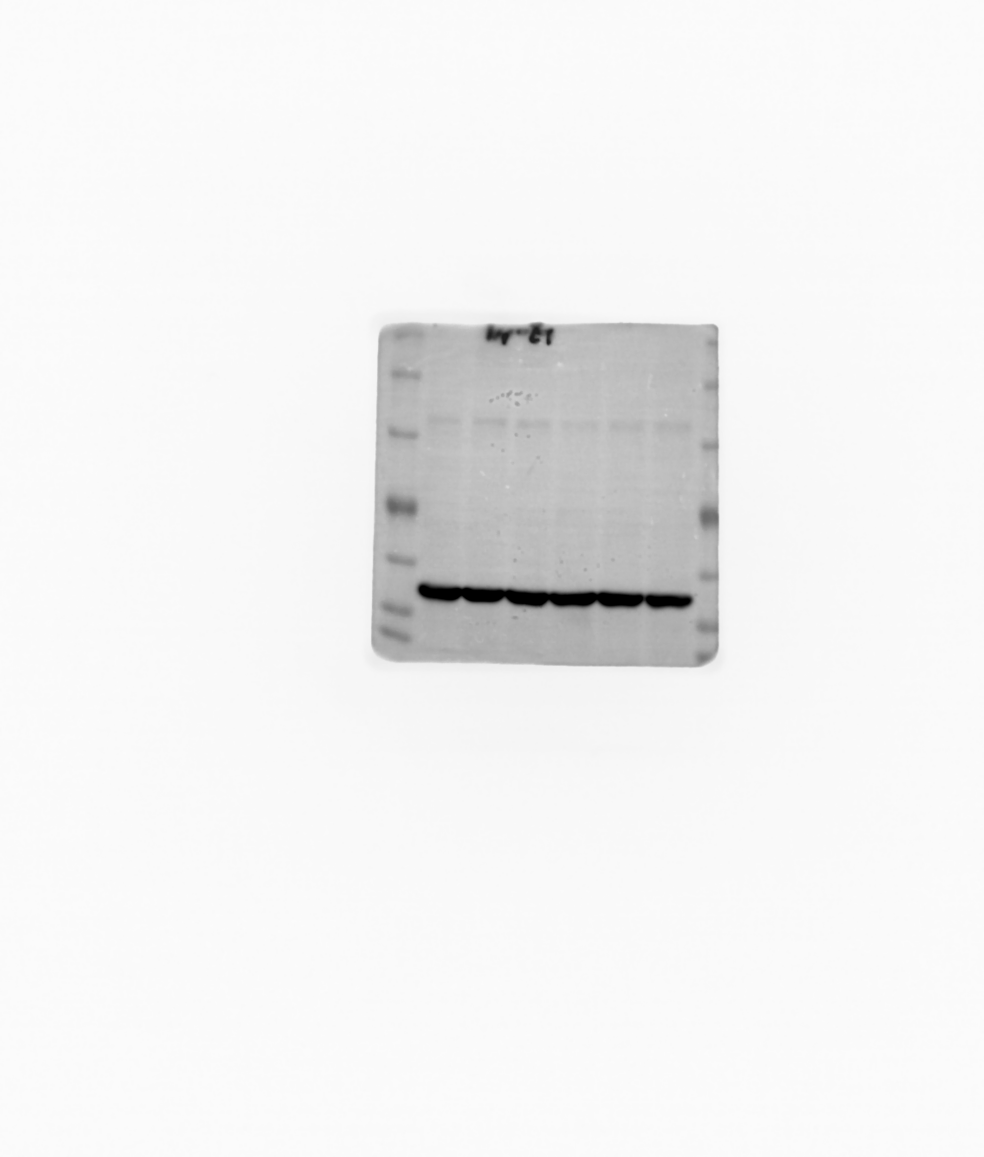


40KD


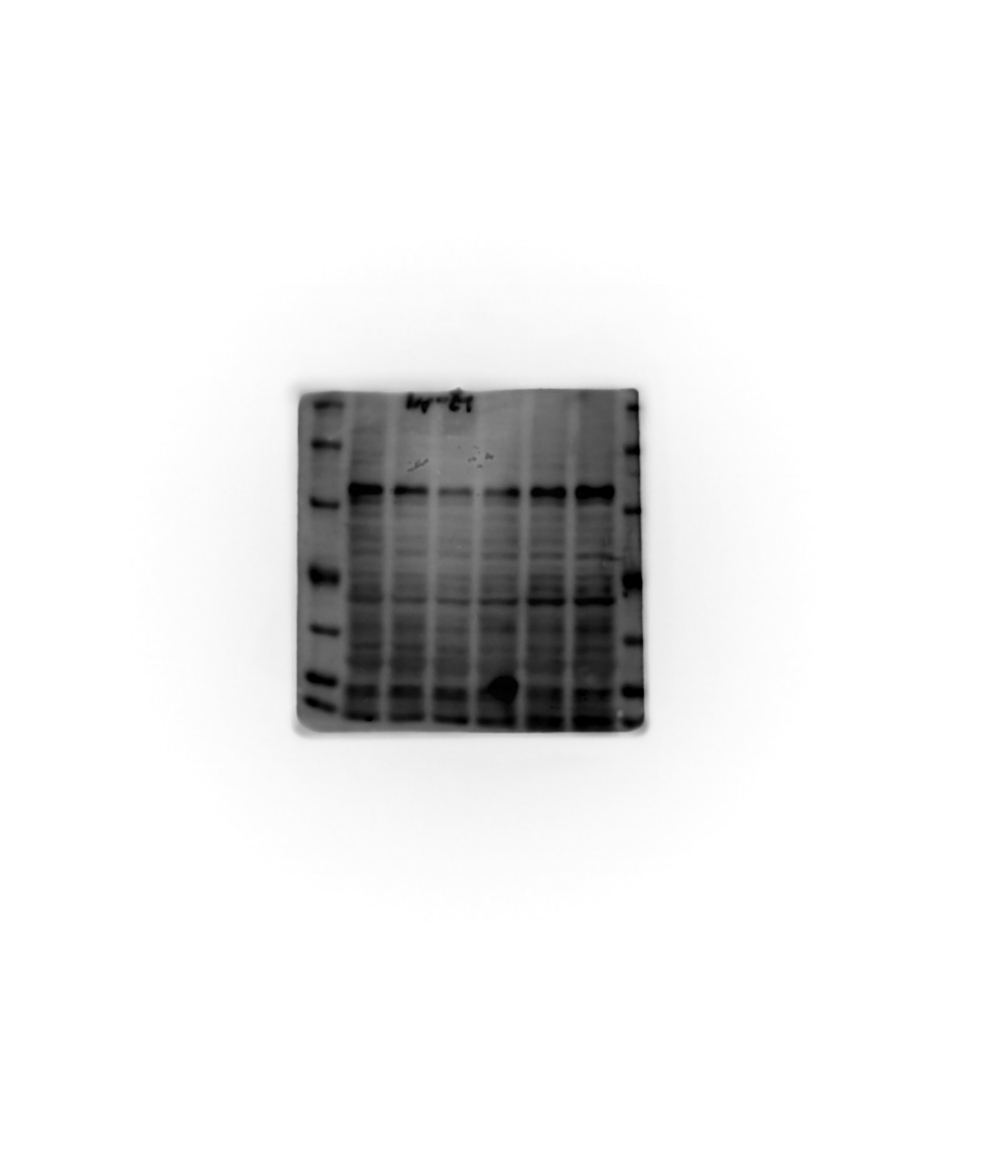


100KD

NS+ABX/LPS

AKK(L)+ABX/LPS

AKK(M)+ABX/LPS

AKK(H)+ABX/LPS

SB203580+ABX/LPS

NC

NS+ABX/LPS

AKK(L)+ABX/LPS

AKK(M)+ABX/LPS

AKK(H)+ABX/LPS

SB203580+ABX/LPS

NC

Nrf2 β-actin


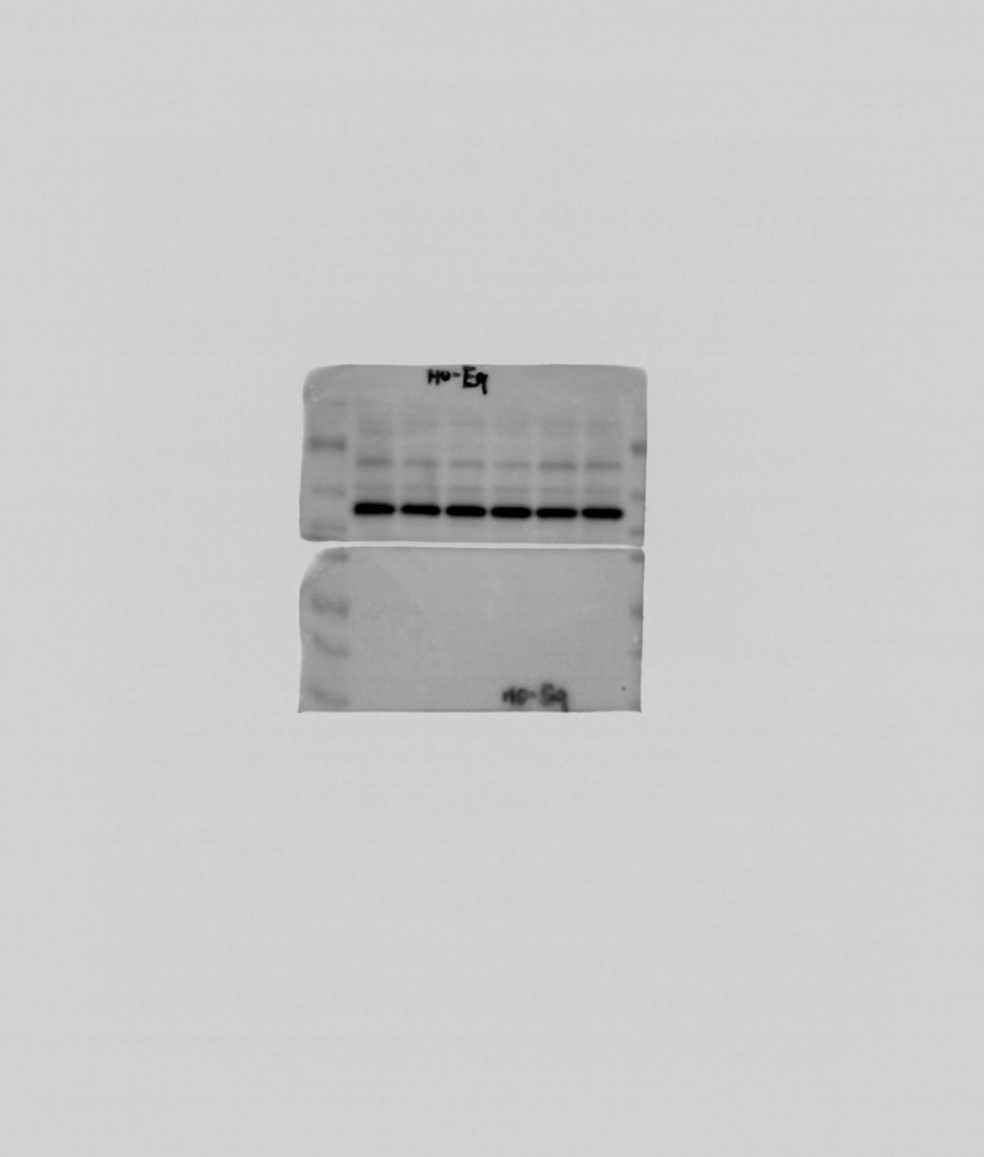


40KD


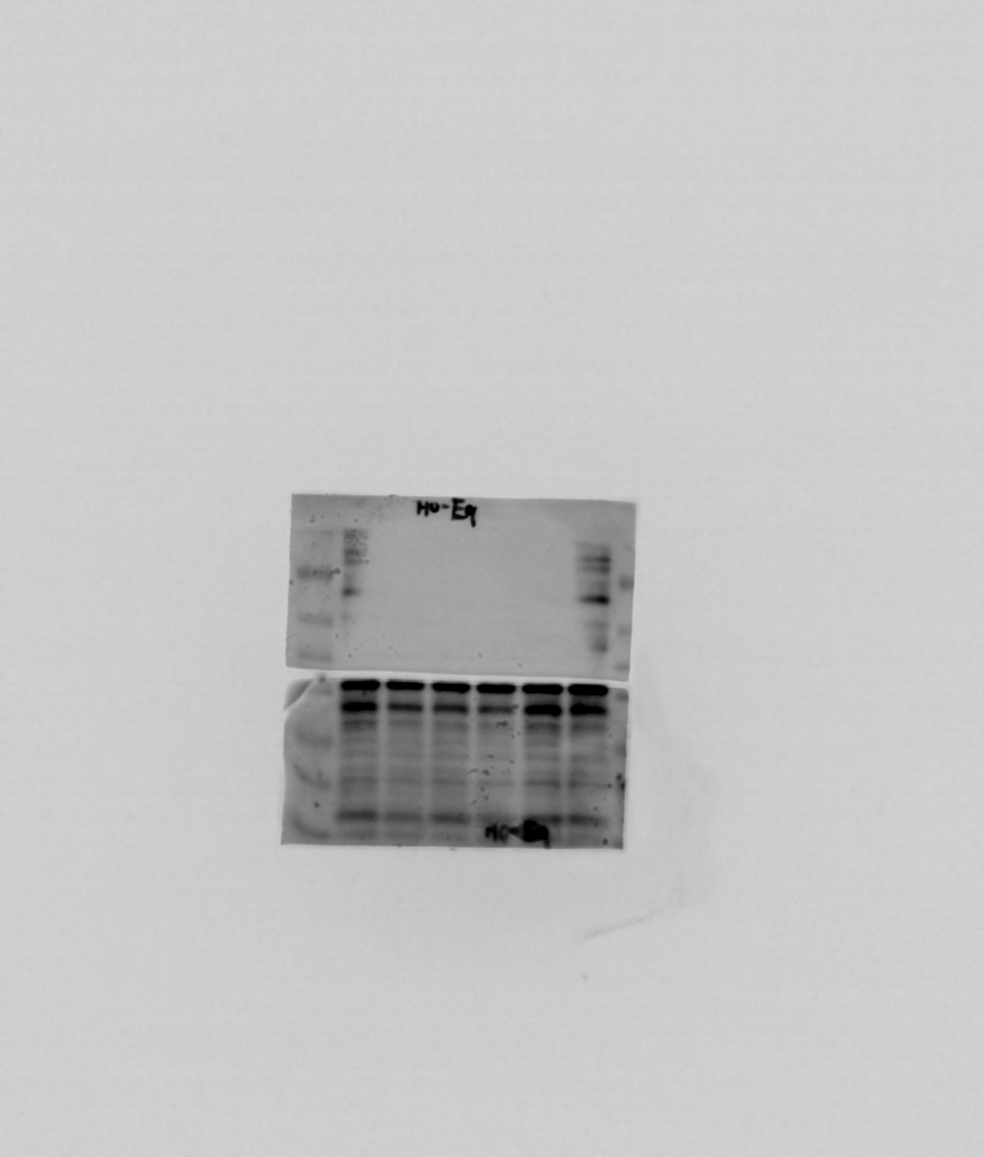


35KD

30KD

NS+ABX/LPS

AKK(L)+ABX/LPS

AKK(M)+ABX/LPS

AKK(H)+ABX/LPS

SB203580+ABX/LPS

NC

NS+ABX/LPS

AKK(L)+ABX/LPS

AKK(M)+ABX/LPS

AKK(H)+ABX/LPS

SB203580+ABX/LPS

NC

HO-1 β-actin


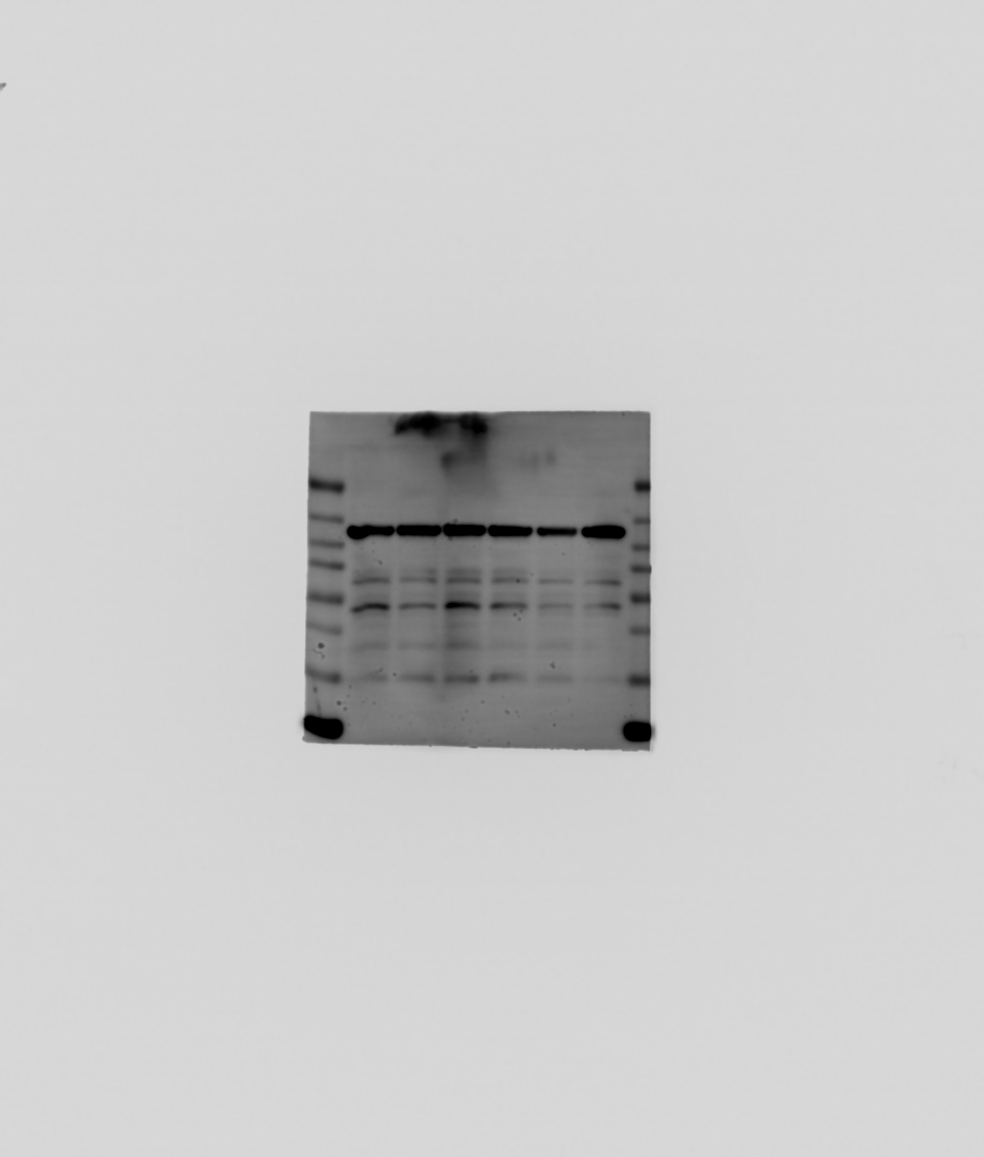


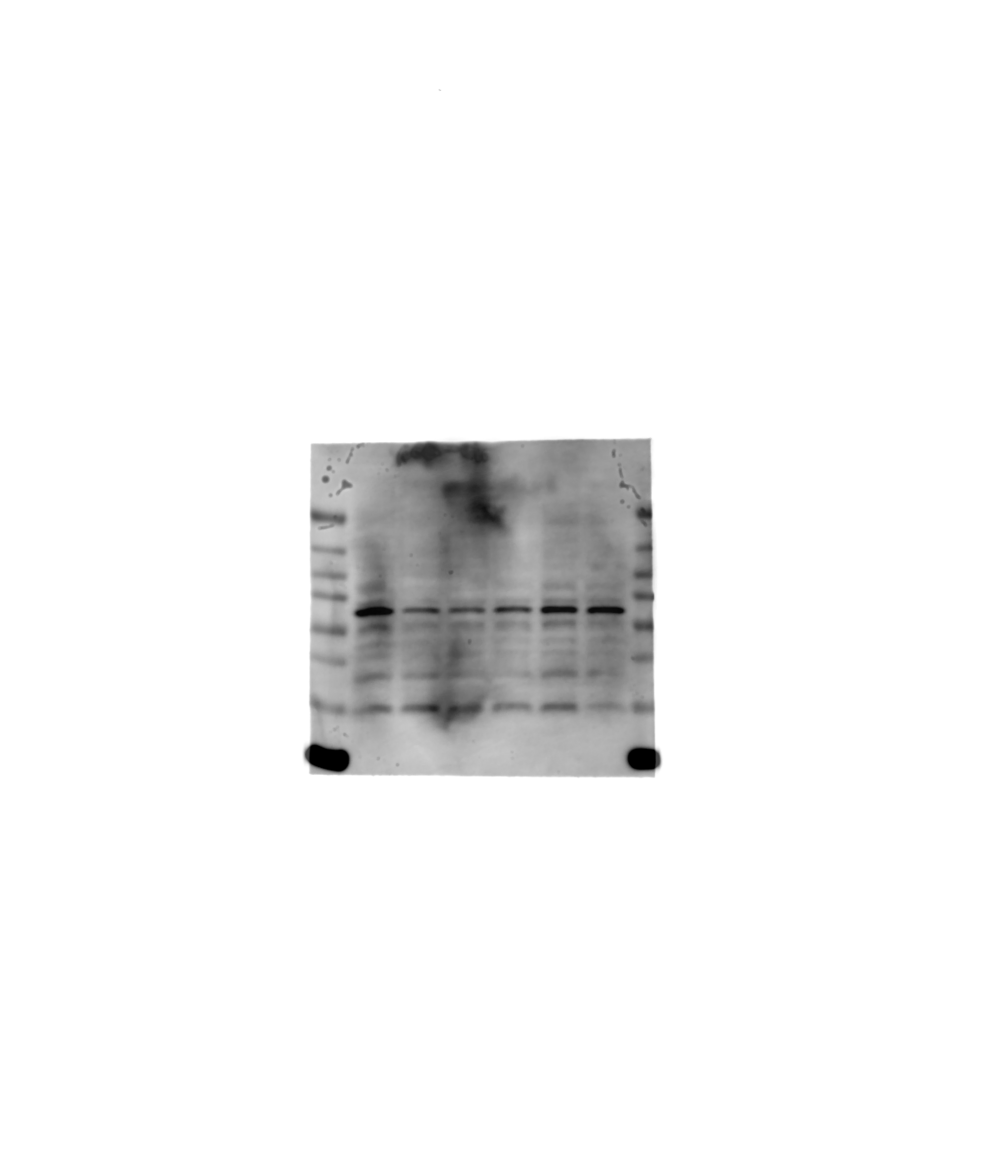


30KD

35KD

40KD

NS+ABX/LPS

AKK(L)+ABX/LPS

AKK(M)+ABX/LPS

AKK(H)+ABX/LPS

SB203580+ABX/LPS

NC

NS+ABX/LPS

AKK(L)+ABX/LPS

AKK(M)+ABX/LPS

AKK(H)+ABX/LPS

SB203580+ABX/LPS

NC

NQO-1 β-actin

**Figure 6**

**
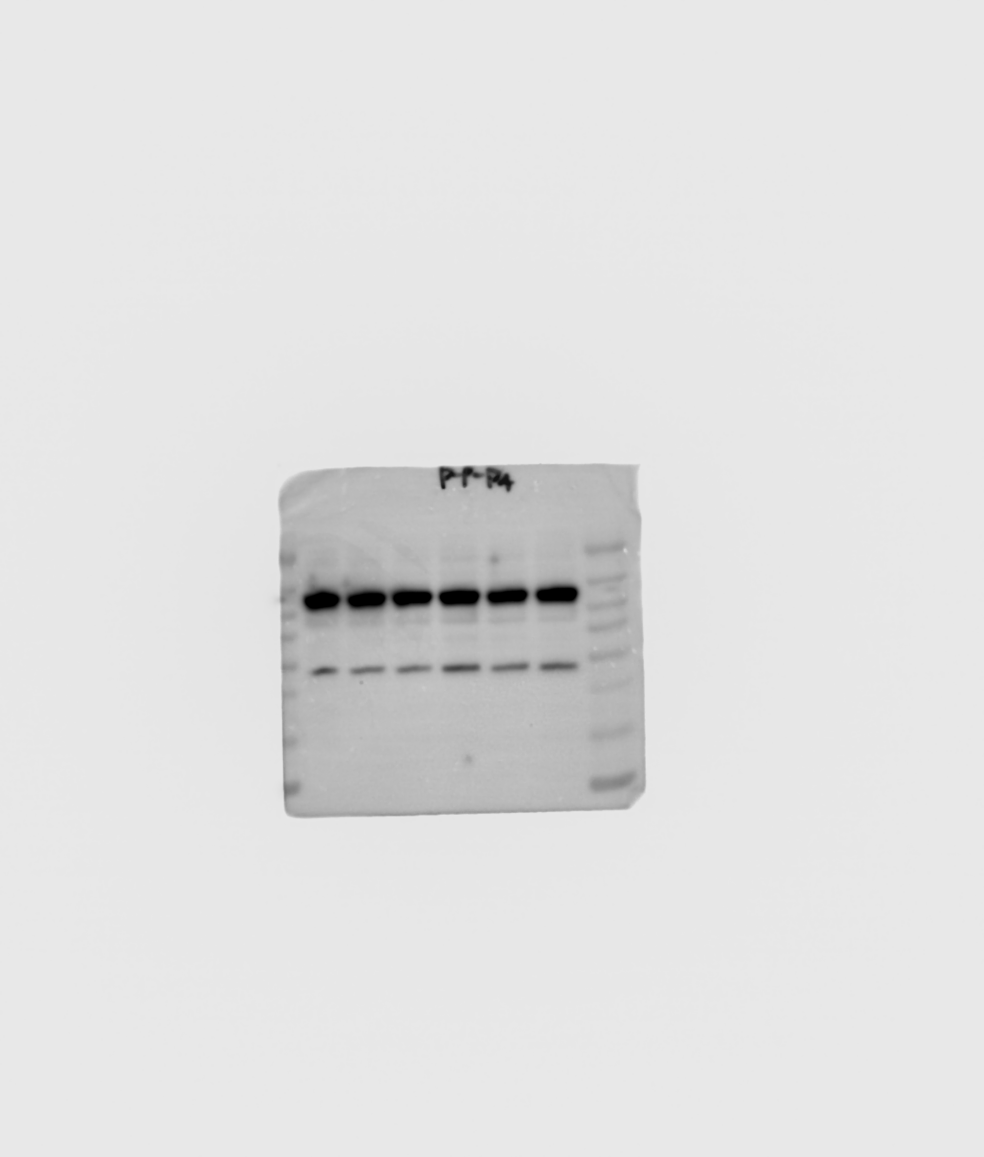

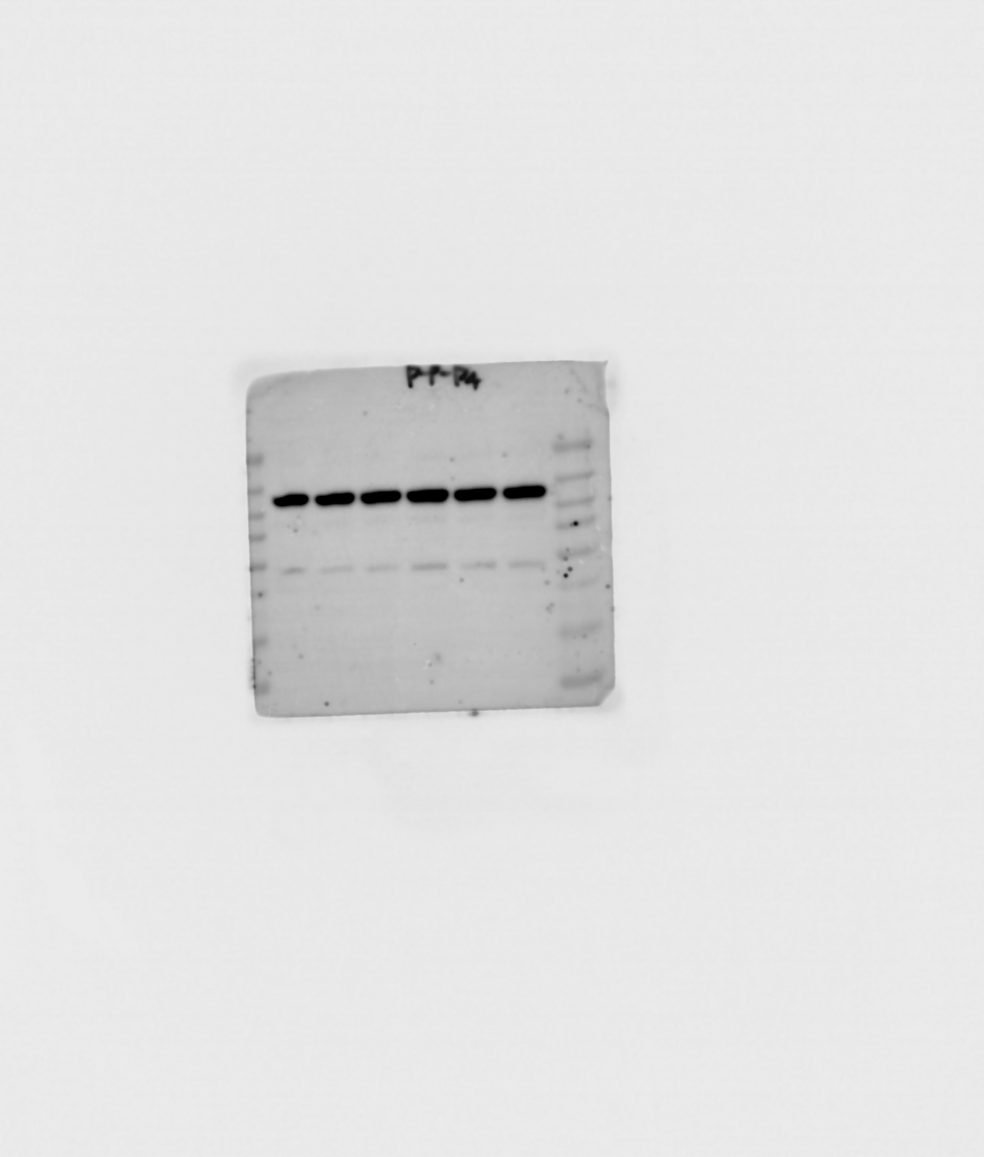

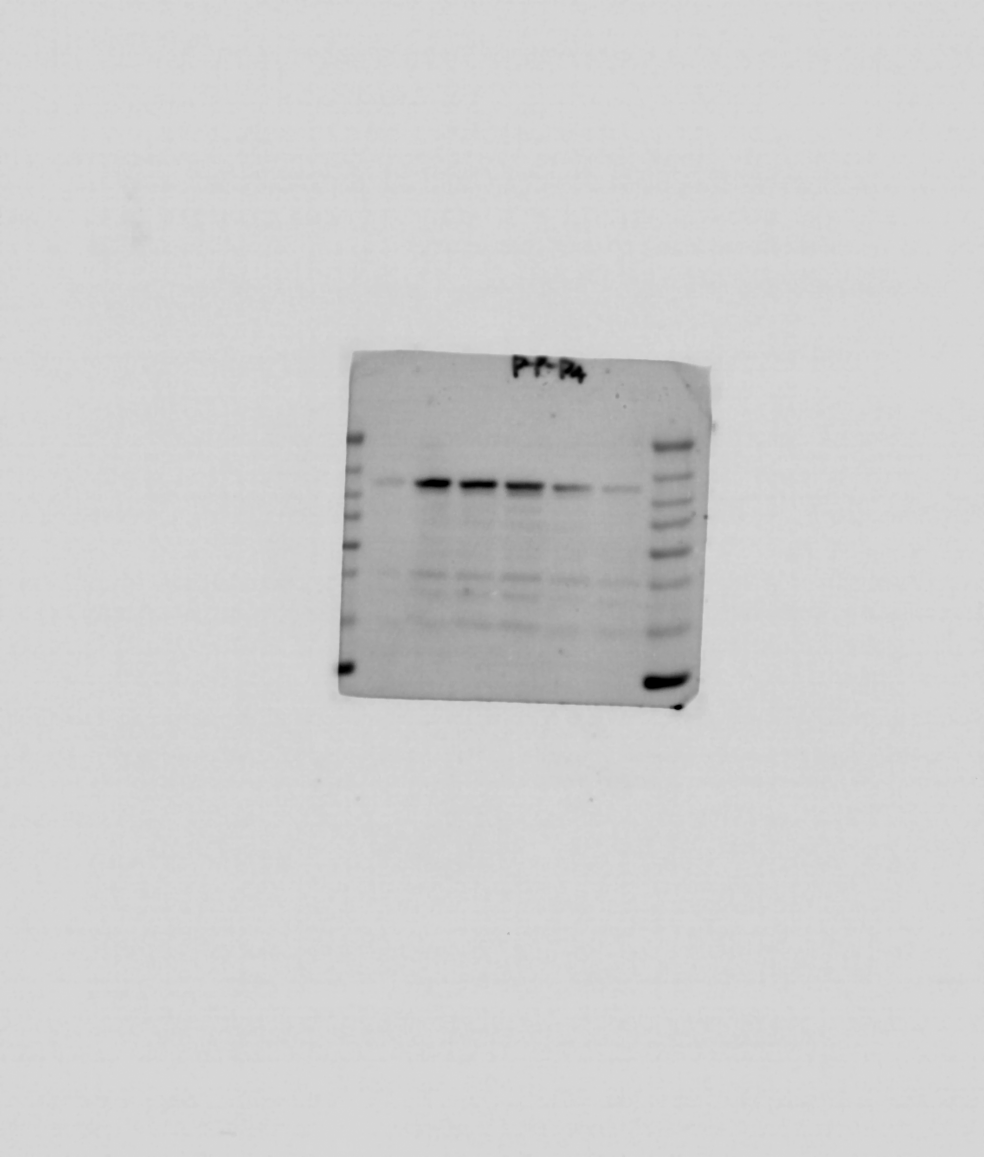
**

40KD

NS+ABX/LPS

AKK(L)+ABX/LPS

AKK(M)+ABX/LPS

AKK(H)+ABX/LPS

SB203580+ABX/LPS

NC

NS+ABX/LPS

AKK(L)+ABX/LPS

AKK(M)+ABX/LPS

AKK(H)+ABX/LPS

SB203580+ABX/LPS

NC

NS+ABX/LPS

AKK(L)+ABX/LPS

AKK(M)+ABX/LPS

AKK(H)+ABX/LPS

SB203580+ABX/LPS

NC

p-p38αMAPK p38αMAPK β-actin

**Figure 7**
