# Supplementary material for: Akkermansia muciniphila alleviates antibiotic- and LPS-induced oxidative stress via the p38α MAPK–Nrf2 signaling axis
Source: Front Microbiol. 2026 Feb 10;17:1753421. doi: 10.3389/fmicb.2026.1753421 (PMC12931279; doi:10.3389/fmicb.2026.1753421)
Supplement: Supplementary file 3 [file Supplementary_file_3.pdf]

FIRST AFFILIATED HOSPITAL of GUANGXI MEDICAL UNIVERSITY
ETHICAL REVIEW COMMITTEE Approval Notice

Approval Number: 2026-E0054

Title: Akkermansia muciniphila alleviates antibiotic- and LPS-induced oxidative stress via the p38α MAPK–Nrf2 signaling axis

Research Contents: Antibiotic abuse and subsequent infection induce dysregulation of the intestinal epithelial kinome, characterized by p38α hyperphosphorylation (encoded by MAPK14), serving as a common molecular trigger for barrier failure. However, readily druggable nodes to repair this dysregulation remain elusive. In an antibiotic-LPS co-exposure enteropathy model, we found that Akkermansia muciniphila (AKK) reactivates the "p38α MAPK–Nrf2" signaling pathway. Mechanistically, AKK specifically alleviates p38α subtype-mediated suppression of Nrf2, thereby synergistically enhancing the expression of antioxidant enzymes such as HO-1 and NQO1, reducing excessive reactive oxygen species (ROS) production, and restoring the integrity of epithelial tight junctions and mucus layers. Our work is the first to establish the "AKK–p38α MAPK–Nrf2" axis as a druggable kinase module for antibiotic-associated intestinal disease, providing an immediately translatable molecular foundation for developing oral, mechanism-defined, and precise microecological therapies.

Applicant: Qing-Wen Shan

Application Department: Department of Pediatrics, The First Affiliated Hospital of Guangxi Medical University

Date of Application: January 22, 2026

Date of Approval: January 23, 2026

Conclusion: This paper fully considered and protected the rights and interests of the study objects. It meets the criteria of Ethical Review Committee. The Medical Ethics Committee of First Affiliated Hospital of Guangxi Medical University has approved the protocol.

Signature:

(Vice) Director of Ethical Review Committee

First Affiliated Hospital of Guangxi Medical University

Date: January 23, 2026
